# Genomic Analyses Identify Novel Molecular Signatures Specific for the *Caenorhabditis* and other Nematode Taxa Providing Novel Means for Genetic and Biochemical Studies

**DOI:** 10.3390/genes10100739

**Published:** 2019-09-24

**Authors:** Bijendra Khadka, Tonuka Chatterjee, Bhagwati P. Gupta, Radhey S. Gupta

**Affiliations:** 1 Department of Biochemistry and Biomedical Sciences, McMaster University, Hamilton, Ontario L9H 6K5, Canada; khadkab@mcmaster.ca (B.K.); tinaburi02@hotmail.com (T.C.); 2 Department of Biology, McMaster University, Hamilton, Ontario L8N 3Z5, Canada; guptab@mcmaster.ca

**Keywords:** genome sequences, molecular markers (synapomorphies), phylogenetic trees, conserved signature indels, *Caenorhabditis elegans*, *Chromadorea*, structural analysis of *Caenorhabditis*/nematodes-specific indels, evolutionary relationships among nematodes

## Abstract

The phylum Nematoda encompasses numerous free-living as well as parasitic members, including the widely used animal model *Caenorhabditis elegans*, with significant impact on human health, agriculture, and environment. In view of the importance of nematodes, it is of much interest to identify novel molecular characteristics that are distinctive features of this phylum, or specific taxonomic groups/clades within it, thereby providing innovative means for diagnostics as well as genetic and biochemical studies. Using genome sequences for 52 available nematodes, a robust phylogenetic tree was constructed based on concatenated sequences of 17 conserved proteins. The branching of species in this tree provides important insights into the evolutionary relationships among the studied nematode species. In parallel, detailed comparative analyses on protein sequences from nematodes (*Caenorhabditis*) species reported here have identified 52 novel molecular signatures (or synapomorphies) consisting of conserved signature indels (CSIs) in different proteins, which are uniquely shared by the homologs from either all genome-sequenced *Caenorhabditis* species or a number of higher taxonomic clades of nematodes encompassing this genus. Of these molecular signatures, 39 CSIs in proteins involved in diverse functions are uniquely present in all *Caenorhabditis* species providing reliable means for distinguishing this group of nematodes in molecular terms. The remainder of the CSIs are specific for a number of higher clades of nematodes and offer important insights into the evolutionary relationships among these species. The structural locations of some of the nematodes-specific CSIs were also mapped in the structural models of the corresponding proteins. All of the studied CSIs are localized within the surface-exposed loops of the proteins suggesting that they may potentially be involved in mediating novel protein–protein or protein–ligand interactions, which are specific for these groups of nematodes. The identified CSIs, due to their exclusivity for the indicated groups, provide reliable means for the identification of species within these nematodes groups in molecular terms. Further, due to the predicted roles of these CSIs in cellular functions, they provide important tools for genetic and biochemical studies in *Caenorhabditis* and other nematodes.

## 1. Introduction

Animals of the phylum Nematoda represent a large and diverse group of eukaryotes present in various marine, freshwater, and terrestrial ecosystems [1,2,3]. Of the >1 million nematode species that are indicated to exist, approximately 25000 species are currently recognized [3,4,5]. Most nematodes are transparent and small organisms. However, some can grow to lengths of several meters [3,6]. There are two major types of nematodes in terms of their trophic ecology, i.e., those which are free-living and others which are parasitic [1,5,6,7]. Free-living nematodes are found in all types of environments and feed on bacteria, algae, or fungi [5,6,8]. Parasitic nematodes occupy and obtain nutrients from various host organisms including animals, plants, and insects [1,6,8,9,10,11].

Nematodes play significant roles in diverse environments and several parasitic species cause extensive damage to agricultural crops, harm livestock and are also a threat to humans [1,2,5]. For example, root-knot nematodes from the genus *Meloidogyne* damage soybean, potato, and sugar beet crops, resulting in large losses to the agricultural industry [2]. *Haemonchus contortus* and *Ascaris suum* are animal parasitic species known to infect sheep and pigs, respectively [6]. Moreover, some nematode species belonging to the genera *Trichinella* and *Trichuris* infect humans and can cause severe gastrointestinal problems, which, in some cases, can result in death [12,13]. However, some nematode species, specifically *Caenorhabditis elegans* (*C. elegans*), have found wide-spread usage as important model organisms for studies related to cellular development, aging and other genetic, biochemical, and cell biological studies [14,15,16,17,18,19,20]. Among the many advantages of *C. elegans* as a model system, it is a transparent microscopic organism with nervous system and all its neurons have been mapped. Further, it has a short life cycle and it is easy and inexpensive to maintain in a lab, and can be readily manipulated genetically [2,3,21,22]. More importantly, *C. elegans* contains a number of genes that are similar and homologous to the human disease genes, making it an ideal organism to study human diseases in an animal model [2,3,15,17,22,23,24,25,26]. The global concerns of animal and plant-parasitic nematodes, as well as the medical and agricultural applications of other free-living and parasitic nematodes, underscore the need to understand the evolutionary relationships as well as novel characteristics of different groups of nematodes.

Earlier studies on the classification of nematodes were based on morphological characteristics [1,5,6,7]. However, as most of the studied morphological traits were homoplasious (i.e., shared presence was not due to common ancestry), the resulting classification was misleading [1,6,27]. In recent years, phylogenetic studies employing predominantly 18S and 28S ribosomal RNA (rRNA) genes and mitochondrial DNA have been used to examine the evolutionary relationships among nematodes [1,2,4,5,6,21,28,29,30,31,32]. However, these studies are often unable to discriminate between species of higher-level nematode taxa [33,34]. Further, as the branching of species in phylogenetic trees is affected by large numbers of variables [35,36,37,38,39], additional more reliable means for distinguishing different main groups of nematodes are needed. Currently, very few reliable molecular characteristics are known that are specific for the genus *Caenorhabditis* or other nematodes, which could be used to confidently discriminate important groups of nematodes in molecular terms.

Genome sequences are currently available for 52 nematode species, providing good coverage of several important groups within the phylum Nematoda [40]. These sequences serve as a valuable resource for a more reliable understanding of the evolutionary relationships amongst the species [41] and for identifying novel molecular characteristics that are uniquely shared within specific groups/clades of nematodes. Additionally, the sequence data offers powerful means for genetic, biochemical studies, and other types of studies including identification of novel drug targets [9,11,30,42,43,44]. One important class of molecular markers whose discovery has been facilitated by genome sequence analyses is comprised of conserved signature indels (insertions/deletions) (CSIs) in gene/protein sequences that are uniquely shared by an evolutionarily related group of species [37,38,45,46]. The CSIs that are useful for evolutionary studies are generally of specific lengths, present at specific positions in particular genes/proteins, and they are flanked on both sides by conserved regions to ensure that they constitute reliable characteristics [37,47,48,49,50]. The CSIs in gene/protein sequences generally result from rare genetic changes and they have provided important means for demarcation of different groups of organisms in molecular terms [37,38,45,48,51]. Further, based upon their presence or absence in different species, important inferences regarding the evolutionary relationships among a given group of species can be derived [37,38,45].

In the present study, we have used the genome sequences of 52 nematode species to construct a phylogenetic tree for the nematodes based on concatenated sequences of 17 conserved proteins. This tree provides important insights into the evolutionary relationships amongst the nematodes, and a number of major groups/taxa within the phylum Nematoda are reliably resolved. More importantly, our comparative genomic analysis of the protein sequences of *Caenorhabditis* species has uncovered 52 molecular signatures comprising of CSIs in diverse proteins that are uniquely shared by either all sequenced *Caenorhabditis* species or by several higher taxa of nematodes encompassing this genus. Of these molecular markers, 39 CSIs in proteins involved in diverse functions are distinctive characteristics of homologs from all six genome-sequenced *Caenorhabditis* species. The described molecular markers, due to their exclusivity for the specific groups of nematodes, provide useful means for the development of novel diagnostics as well as for genetic and biochemical studies on this important group of organisms.

## 2. Materials and Methods

### 2.1. Construction of Phylogenetic Trees

To construct a phylogenetic tree for 52 genome sequenced nematode species, sequences of 17 conserved proteins involved in a variety of cellular functions, which were present in a single copy in these genomes were identified (Table S1). Sequences for four outgroup species viz. *Cryptosporidium muris, Plasmodium falciparum, Babesia sp. Xinjiang* and *Eimeria necatrix*, were used for the rooting of the tree. The phylogenetic tree construction was carried out using an internally developed pipeline described in our earlier work [52]. Briefly, the CD-HIT program was used [53] to identify protein families sharing a minimum of 50% in sequence identity and sequence length and which were found in at least 80% of the input genomes. The Clustal Omega [54] algorithm was used to generate multiple sequence alignment (MSA) of these protein families. The aligned protein families were trimmed with TrimAl [55] to remove poorly aligned regions [56] before concatenation to the other proteins. This concatenated sequence alignment consisting of 10764 aligned amino acids positions was used for phylogenetic analysis. An approximate maximum likelihood (ML) tree based on this sequence alignment was initially constructed in FastTree 2 [57] using the Whelan and Goldman model of protein sequence evolution [58]. The resulting tree was then used as input for RAxML [59], where the Le and Gascuel model of protein sequence evolution [60] in RAxML 8 to optimize individual branch lengths and to identify the optimal maximum-likelihood topology. Optimization of the robustness of the tree was completed by conducting SH tests [61] in RAxML 8 [59]. The sequence alignment created by the above program was also used to construct an ML tree based on 100 bootstrap replicates in MEGA6 [62] using Whelan and Goldman +Freq. model [58] and JTT matrix-based model [62]. 

### 2.2. Identification of Conserved Signature Indels (CSIs)

To identify potential CSIs specific for different groups within the phylum Nematoda, BLASTp searches were performed on >11800 proteins from *Caenorhabditis elegans* genome (from accession number NP_001033396.1 to NP_001343573.1) covering approximately 40% of the annotated proteins. Based on these blast searches, proteins for which high scoring homologs (E-values less than 1e^−20^) were present in multiple nematode species, as well as several non-nematode organisms, were identified and the sequences of these proteins from 15–25 species were retrieved. It was not essential that sequences from any Apicomplexa species (used to root the phylogenetic tree) be present among the sequences for non-nematode species. Multiple sequence alignments for the selected protein sequences were created using CLUSTAL_X 2.1 [63] and these alignments were examined manually to identify insertions or deletions (indels), which were flanked by at least four to five conserved amino acid residues on both sides within the neighboring 40–50 residues [37,47,64]. Indels which were not flanked by conserved regions were not further considered as they do not provide reliable molecular characteristics. Query sequences encompassing the indel and its flanking 40–50 amino acids were collected for all potential CSIs. Afterward, the query sequences underwent another BLASTp search carried out against the NCBI nr database. The resulting top 250–500 hits for all queries were examined to identify CSIs that are uniquely found in the nematode species as well as to evaluate the group specificities of these CSIs. Signature files for all useful CSIs, which were specifically found in the indicated nematode groups, were created using SIG_CREATE and SIG_STYLE programs described in our earlier work [47] that are available on the GLEANS (Gleans.net) server. The CSIs reported here, unless otherwise indicated, are specific for all members of the indicated groups whose homologs were detected by BLASTp searches.

### 2.3. Homology Modelling and Analysis of Protein Structures

Homology models of some proteins which contain the CSIs were created for the *C. elegans* homolog to map the locations of the CSIs in the proteins’ structures. The homology models of the *C. elegans* Rab44 protein, poly ADP-ribose glycohydrolase protein and tRNA guanine N methyltransferase proteins were created using the solved structures of the following template proteins PDB ID: 2p5s (human), PDB ID: 6hmm (human) [65] and PDB ID: 4jwg (from (*Schizosaccharomyces pombe*) [66], respectively. Homology modeling was performed using MODELLER v9.15 [67] and the top 500 models were ranked on the basis of their discrete optimized protein energy (DOPE) scores [68]. The stereo-chemical properties of the final models were assessed using three independent servers: ERRAT, PROSA, and Verify3D [69,70,71,72]. These applications utilize a dataset of refined structures to evaluate the statistical significance of the models’ conformation, location, environment of each amino acid sequence and overall structural stability. Selected models were then refined using ModRefiner [73]. These resultant models were then used to explore the structural changes associated with the insertion. The superimposition of the validated models with the template structures was carried out using PyMOL (http://www.pymol.org) to examine the structure and location of identified CSIs in the modeled protein structures. 

## 3. Results

### 3.1. Phylogenetic Analysis of Nematodes Based on Concatenated Sequences of Conserved Proteins

**Figure 1:**
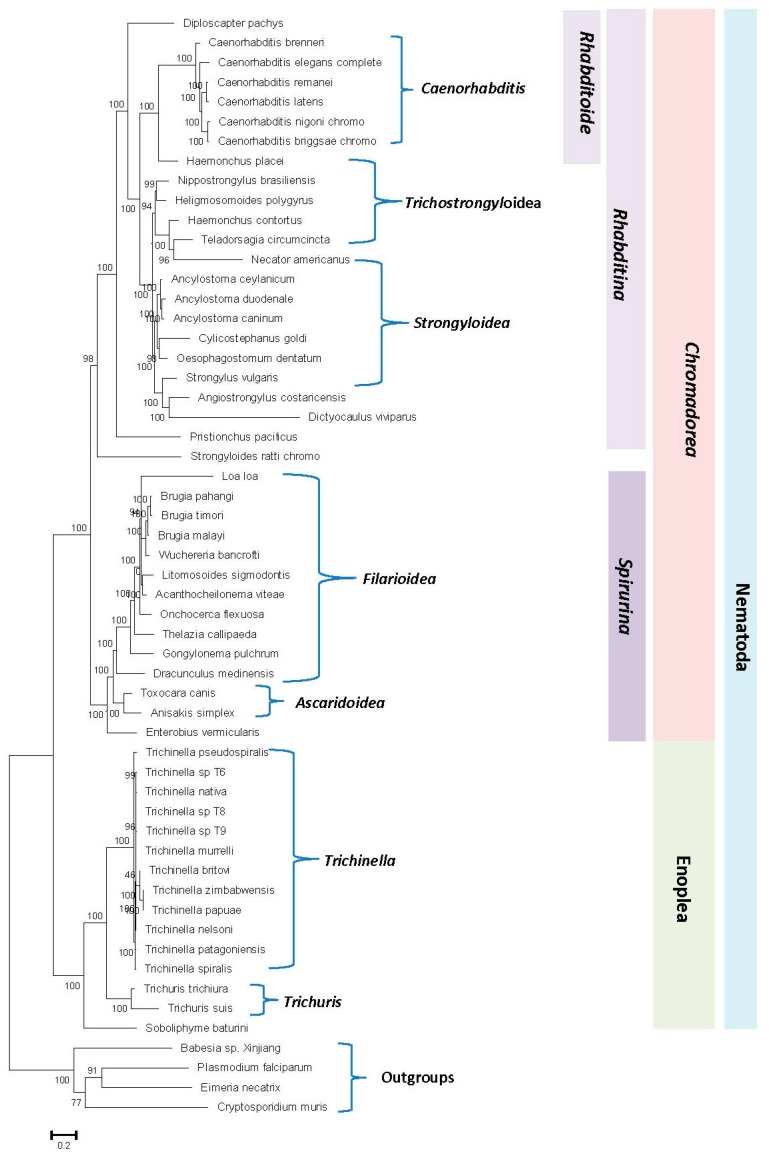
Maximum-likelihood tree for 52 genome-sequenced nematode species. The tree was constructed based on the concatenated alignment of 17 orthologous proteins present in a single copy in these genomes as described in the Methods. Bootstrap scores for each node are indicated at the branch points. The bar indicates 0.2 changes per position. The major nematode groups at different phylogenetic levels are labeled. The tree was rooted using the outgroup species shown.

Evolutionary relationships of the nematodes species in the past have been mainly studied based on gene sequences for 18S or 28S rRNA and mitochondrial proteins [2,4,6,34]. Genome sequences are now available for 52 nematodes species covering a number of major groups/taxa within this phylum. These sequences can be used to examine the phylogenetic relationships among nematode species based on concatenated sequences for multiple conserved proteins. Hence, a maximum-likelihood (ML) phylogenetic tree was constructed for the 52 genome-sequenced nematodes species based on concatenated sequences of 17 conserved proteins. The proteins employed in these analyses, listed in Table S1, are present in a single copy in the available nematodes genomes. The resulting bootstrapped tree, which was rooted using homologous sequences from representative Apicomplexa species, is shown in Figure 1. In this tree, members from the two main classes within the phylum Nematoda, i.e., *Chromadorea* and *Enoplea*, were clearly separated from each other. 

Within the class *Chromadorea*, the species from the two suborders *Rhabditina* and *Spirulina* were also separated from each other. Additionally, species from a number of nematode genera for which sequences were available from multiple species viz. *Caenorhabditis, Ancyclostoma, Trichinella, Trichuris,* and *Brugia*, also formed monophyletic clades supporting the close relationships of species within these genera. However, in the constructed tree, species from the superfamilies *Rhabiditoidea, Strongyloidea*, *Trichostrongyloidea, Filarioidea,* and *Ascarioidea* exhibited polyphyletic branching within each other. Thus, these families cannot be reliably demarcated on the basis of constructed phylogenetic tree and the interrelationships as well as grouping of species within these families remains unclear at present. The branching patterns, as well as the interrelationships among different nematode species observed in our tree, are similar to that reported recently by Smythe et al. [41] based on phylogenomic analysis using a conservative orthology inference strategy. We have also constructed ML trees based on our protein sequences using MEGA6 program employing two different amino acid substitution models, and the results obtained (Figure S1) are very similar to that seen in Figure 1. However, despite the noted limitations of the tree shown in Figure 1, it provides a good phylogenetic framework for understanding and analyzing the results obtained from comparative genomic analysis, which are discussed below.

### 3.2. Identification of Conserved Signature Indels Specific for Different Nematode Groups

While the phylogenetic tree shown in Figure 1 allows some inferences to be drawn regarding the evolutionary relationships amongst the nematode species, it is important to confirm these inferences using other independent approaches that are also capable of providing further insights into the evolutionary relationships among nematode species. As noted in the introduction, CSIs in protein sequences that are uniquely shared by a given group of organisms provide an important class of molecular markers that have been proven very useful for evolutionary/taxonomic studies [45,46,48,49,50,51]. Due to the rare and discrete nature of the genetic changes that give rise to CSIs, the presence or absence of CSIs in different lineages (or proteins) is generally not affected by the factors that can confound or limit the reliability of inferences from phylogenetic trees [41,45,47,48,50]. Hence, the CSIs provide powerful means for demarcating different groups of organisms in molecular terms and for understanding evolutionary relationships. Therefore, a major focus of the present study was to perform comprehensive genomic analysis of protein sequences from *Caenorhabditis* species to identify CSIs that are specific for this genus as well as other higher taxonomic groups/taxa of nematodes encompassing these organisms. The results of our analysis, reported below, have led to the identification of 52 novel molecular signatures in the form of CSIs that are uniquely shared by either all *Caenorhabditis* species or different nematodes groups belonging to this phylum. A brief description of the characteristics of the identified CSIs is provided below.

**Figure 2:**
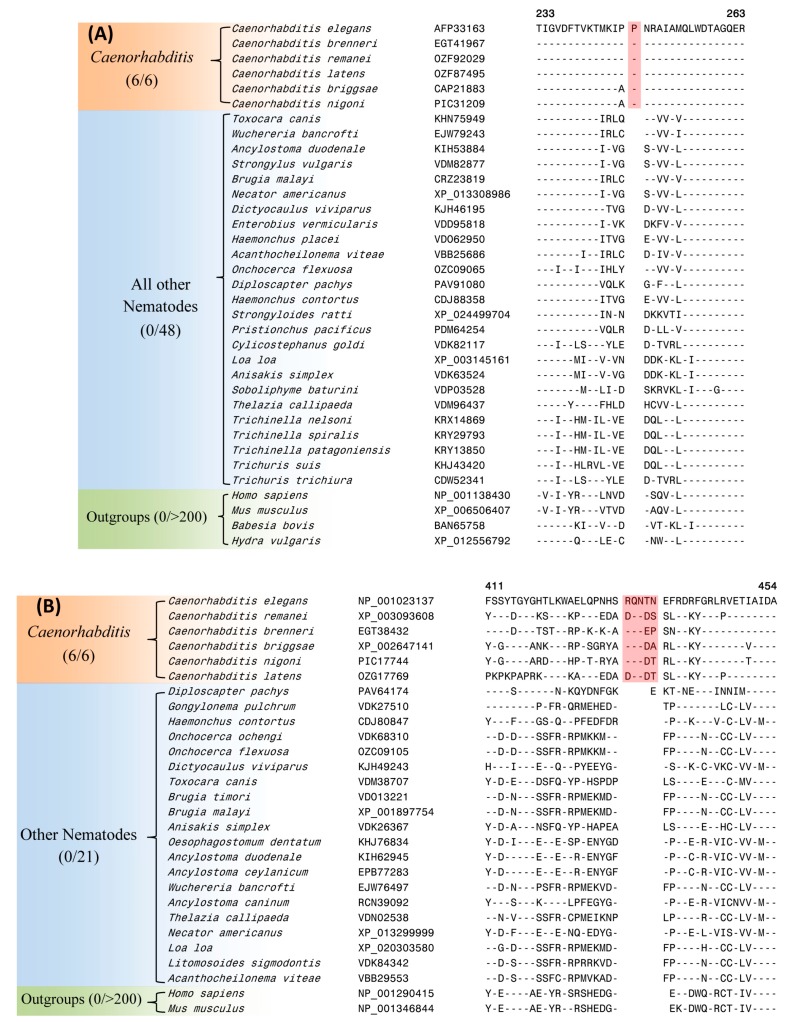
Partial sequence alignments of the proteins (**A**) Rab44 and (**B**) poly ADP-ribose glycohydrolase showing two CSIs (boxed) that are specific for the genus *Caenorhabditis*. Dashes (-) in these as well as all other alignments denote identity with the amino acid shown in the top sequence. Sequence information for only limited numbers of species is presented in this figure. More detailed alignments for these CSIs are shown in Figure S2. Sequence information for 37 additional CSIs, which are also specific for the genus *Caenorhabditis* is provided in Figures S3–S39 and a summary of these CSIs is provided in Table 1.

Of the identified CSIs, 39 CSIs within proteins involved in diverse cellular functions are specifically found in the protein homologs of *Caenorhabditis* species, which form a strongly supported monophyletic clade in our phylogenetic tree. Two examples of such CSIs, one consisting of a 1 amino acid (aa) insertion in Rab44 protein (*C. elegans* gene number 4R79.2) and another comprising a 5 aa insertion in a poly ADP-ribose glycohydrolase protein (PARG-1) are shown in Figure 2A,B, respectively. As seen from Figure 2, both these CSIs are present in conserved regions of the proteins and they are commonly shared by the homologs of all six *Caenorhabditis* species with available genome sequences, but not found in the homologs from other nematodes or non-nematode organisms. Of the two proteins harboring these CSIs, Rab44 is a GTPase of Rab family (Ras superfamily). Although 4R79.2 is yet to be genetically characterized (www.wormbase.org), members of the Rab family act as molecular switches in vesicle trafficking and are known to interact with several other molecules at different trafficking stage [74,75,76]. The protein PARG-1 is a member of poly ADP-ribose glycohydrolase (PARG) family. PARG is a primary enzyme responsible for hydrolyzing the poly(ADP-ribose) polymer synthesized by poly-(ADP-ribose) polymerases and is involved in a variety of nuclear processes such as DNA damage response, development, programmed cell death and aging [65,77]. 

In addition to the two CSIs shown in Figure 2, our study has identified 37 other CSIs in different proteins, which are also specifically found in the homologs from *Caenorhabditis* species. Sequence information for these other CSIs is provided in Figures S3–S39 and some of their characteristics are summarized in Table 1. For some proteins containing these CSIs (viz. an intermediate filament protein, Figure S39), two homologs are present in *Caenorhabditis* species and the described CSI was found in only one of the two homologs. In such cases, it is likely that the two sets of homologs originated from a gene duplication event in a common ancestor of *Caenorhabditis* and the genetic change leading to the observed CSI occurred at this stage in the ancestor of one of the homologs. Due to the exclusive presence of different CSIs listed in Table 1 in the protein homologs for *Caenorhabditis* species, the described CSIs provide reliable molecular markers for distinguishing the members of this genus. The genetic changes responsible for these CSIs are postulated to have occurred in a common ancestor of the genus *Caenorhabditis* during its divergence from other nematodes.

**Figure 3:**
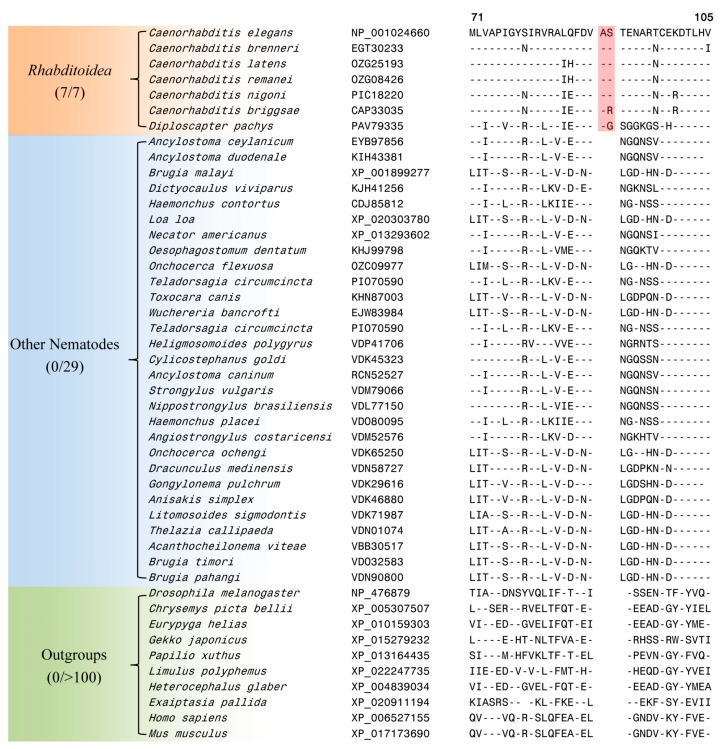
Partial sequence alignment of a conserved region from a protein annotated as abnormal cell migration protein 13 (MIG-13) containing a 2 aa insertion (boxed) which is specific for the family *Rhabditoidea*. This insertion is not present in the homologous proteins from other nematodes as well as other eukaryotic species. Sequence information for three additional CSIs, which are also specific for the family *Rhabditoidea* is provided in Figures S41–S43 and a summary of these CSIs is provided in Table 2. Other details are the same as in the legend to Figure 2.

Our work has also identified 4 CSIs, which, in addition to the *Caenorhabditis* species, are also commonly shared by the species *Diploscapter pachys*. The species from both these genera are part of the family *Rhabditoidea* [3]. One such CSI is a 2 aa insertion in a protein annotated as abnormal cell migration protein 13 (MIG-13), which is specifically found in the homologs from the family *Rhabditoidea* and it is not present in the homologous proteins from other nematodes or other species (see Figure 3). 

Although the exact function of the abnormal cell migration protein MIG-13 has not been elucidated, cell migration and morphogenesis are key events in tissue development and organogenesis [18,20]. MIG-13 is an evolutionarily conserved transmembrane protein that has been shown to play an important role in cell migration in the Q neuroblast lineage [78]. MIG-13 acts cell-autonomously to regulate the asymmetric distribution of the actin cytoskeleton in the leading edge of QR descendants [79,80]. Thus, the presence of a CSI in this protein, which is specific for the family *Rhabditoidea* is of much interest. Sequence information for the other three CSIs, which are also specific for the family *Rhabditoidea* is provided in Figures S41–S43 and information for them is summarized in Table 2. These CSIs provide reliable evidence supporting a grouping of *Diploscapter pachys* with the *Caenorhabditis* species and they can be used to distinguish members of the family *Rhabditoidea* from other nematodes in molecular terms. 

**Figure 4:**
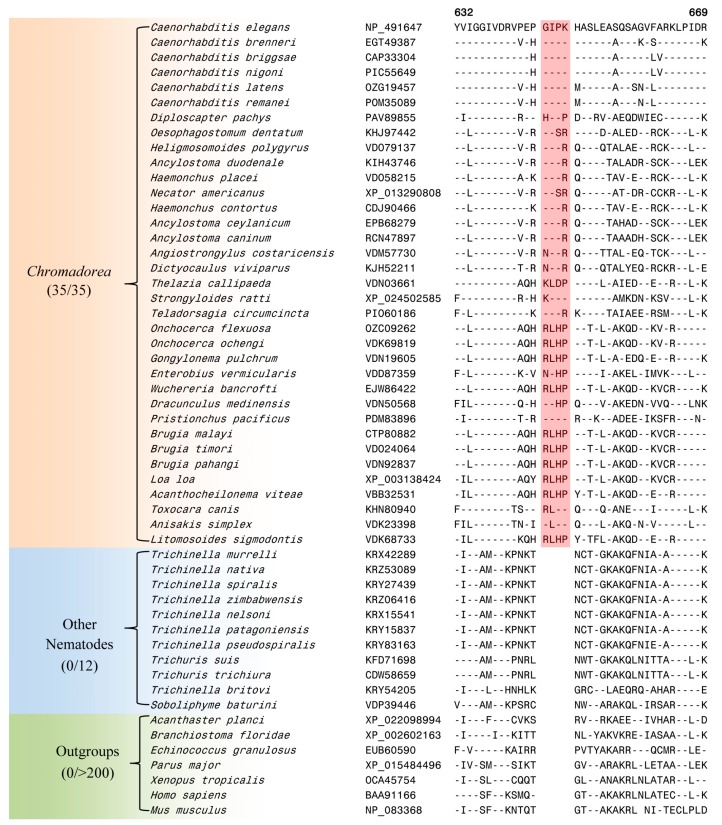
Excerpts from the sequence alignment of a conserved region of the protein tRNA (guanine-N(1)-)-methyltransferase protein containing a 4 aa CSI (boxed) which is specifically found in the homologs from the class *Chromadorea*. Sequence information for seven additional CSIs, which are also specific for the class *Chromadorea* is provided in Figures S44–S50 and a summary of these CSIs is provided in Table 2.

The genus *Caenorhabditis* is embedded within the class *Chromadorea*, which constitutes one of the two main classes within the phylum Nematoda [3,81]. Our analysis has identified eight CSIs in different proteins that are uniquely shared by the homologs from different *Chromadorea* species but absent in nematodes belonging to the class *Enoplea* as well as other organisms. Of these eight CSIs, six CSIs are commonly shared in most cases by all genome-sequenced *Chromadorea* species, whereas in two of them, the described CSIs lack in the species *Strongyloides ratti* (belonging to the suborder *Tylenchina*) [30], which in our phylogenetic tree branches are between the classes *Chromadorea* and *Enoplea.* One example of a CSI that is specific for the class *Chromadorea* is presented in Figure 4. 

In the CSI shown in Figure 4, which is specific for the class *Chromodorea*, a four aa insertion is present in a conserved region of tRNA (guanine-N(1)-)-methyltransferase, which is encoded by F46F11.10 gene in *C. elegans*. The human homolog of this protein plays an essential role in the methylation of specific guanine residues in tRNA molecules [82]. This CSI is uniquely shared by different *Chromadorea* species, but it is absent in other nematodes as well as different other organisms. Sequence information for the other seven CSIs, which are also specific for the class *Chromadorea* is presented in Figures S44–S50 and information for them is summarized in Table 2.

**Figure 5:**
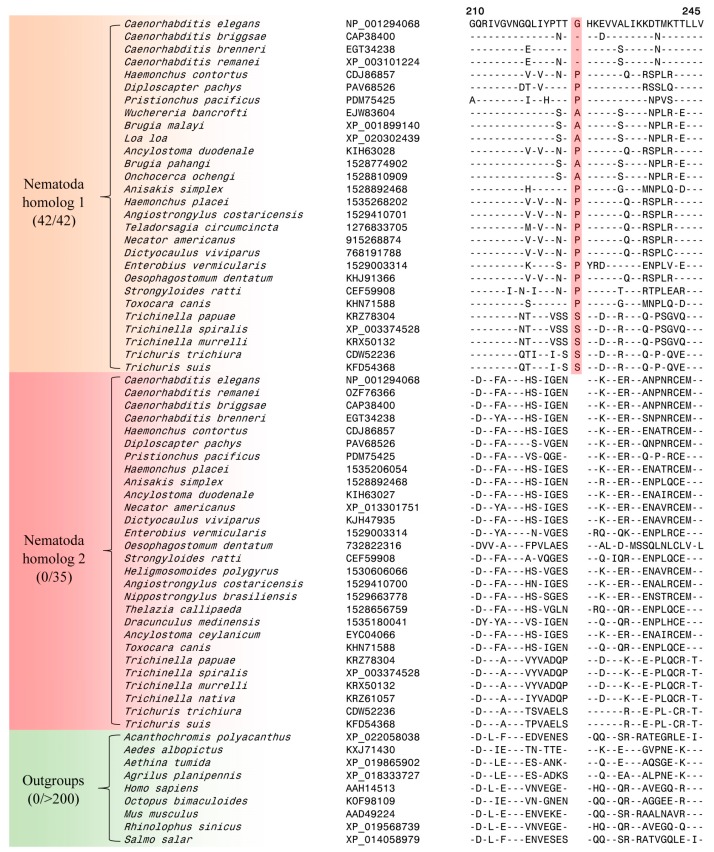
Partial sequence alignment from a conserved region of a Na(+)/H(+) exchange regulatory factor protein (NRFL-1) harboring a 1 aa insertion (boxed) which is specific for the phylum Nematoda. Most nematodes species contain two homologs of this protein and this CSI is specifically present in one of these two homologs. More detailed information regarding the species distribution of this CSI is provided in Figure S51.

Lastly, our analysis has also identified one CSI in a Na(+)/H(+) exchange regulatory factor protein NRFL-1 that appears to be specific for the phylum Nematoda. The *nrfl-1* gene is expressed in many cells and tissues including excretory cell, intestine, pharynx, and tail [83]. NRFL-1 binds to an amino acid transporter AAT-6 to help retain localization of AAT-6 on the intestinal luminal membrane in older worms. Partial sequence alignment of NRFL-1 from nematodes species as well as representative outgroups species are shown in Figure 5. Most nematodes species contain two homologs of *nrfl-1*. Of these two homologs, one contains a single aa insertion within a conserved region that is specifically found in all nematodes species (Figure 5). This insert is absent in the other protein homolog as well as in the homologous protein from different outgroup species. The absence of this insertion in the outgroup species indicates that this indel is an insert and the genetic change leading to it was introduced in a common ancestor of the phylum Nematoda. More detailed information regarding the species distribution of this CSI is provided in Figure S51. 

### 3.3. Localizations of the CSIs in Protein Structures

**Figure 6:**
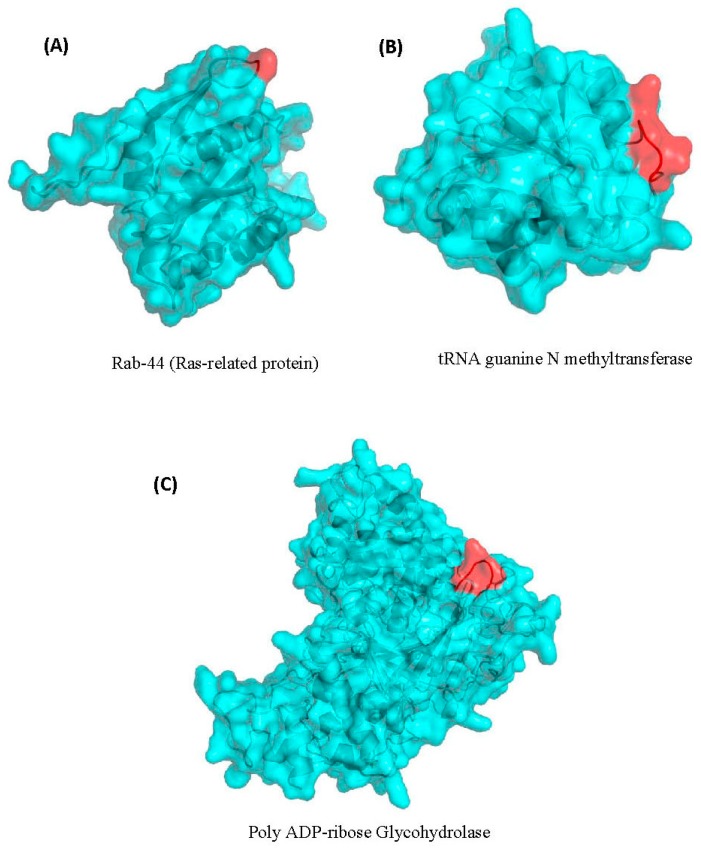
Homology models of the *C. elegans* proteins (**A**) Rab-44, (**B**) poly ADP-ribose glycohydrolase and (**C**) tRNA (guanine-N(1)-)-methyltransferase showing the locations of the CSIs in the structures of these proteins. The CSIs are shown in red color in these figures. As seen from the presented structural overlap, the CSIs in all three studied proteins are localized within the surface-exposed loops of these proteins. More details regarding modeling of these structures are provided in the Methods section.

Earlier work on CSIs in proteins shows that most of the studied CSIs in proteins are located on the surface exposed loops of proteins [84,85,86]. The surface-exposed loops in proteins are known to play important roles in mediating novel protein–protein or protein–ligands interaction [84,87,88]. In view of these earlier studies, we have also examined the locations of some of the nematodes-specific CSIs identified in the structures of the nematodes proteins. The mapping of the CSIs in protein structures was carried out for three different proteins. These proteins included Rab-44 (4R79.2) and poly ADP-ribose glycohydrolase (PARG-1), which contain one and five aa insertions, respectively, that are specific for the *Caenorhabditis* species (Figure 2), and a four aa insertion in the protein tRNA (guanine-N(1)-)-methyltransferase (F46F11.10) (Figure 4) that is specific for the class *Chormadorea*. The structural information for these proteins from *Caenorhabditis* or any other nematode species is presently lacking. However, the structures of their homologs from humans or other eukaryotic organisms exhibiting high sequence similarity to the *C. elegans* homologs are available [65,66] (see Materials and Methods). Using the available structures of these proteins as templates and by means of the homology modeling technique, the structures of the corresponding *C. elegans* proteins were constructed and validated as detailed in the Methods section. To visualize the locations of the identified CSIs in the structures of these proteins, structural overlaps of the modeled proteins containing the CSIs and the solved structures of the proteins lacking the CSIs were carried out. The results of these studies for the proteins Rab-44 (4R79.2), poly ADP-ribose glycohydrolase (PARG-1) and tRNA (guanine-N(1)-)-methyltransferase (F46F11.10) are presented in Figure 6A–C, respectively. The locations of the CSIs in the protein structures are shown in red color in this figure. As seen from the presented structural overlaps, the CSIs in all three studied proteins are localized within the surface-exposed loops of these proteins, which is in accordance with the results of earlier studies [84,85,86,87].

## 4. Discussion

Nematodes species are clinically, economically, and scientifically important organisms. In addition to their significance for human health and agricultural industry due to their animal and plant pathogenicity, they provide very useful model organisms for scientific research relevant to human [1,2,3,5,14,22,89]. Thus, it is of much importance to understand their evolutionary relationships and identify reliable molecular means capable of clearly distinguishing different important groups among nematodes. In this study, we have used available genome sequences of 52 diverse nematode species to examine their evolutionary relationships and have performed a comparative analysis on their protein sequences to identify novel molecular markers that are distinctive characteristics of the *Caenorhabditis* species as well as other groups of nematodes.

Phylogenetic trees based on concatenated sequences for multiple proteins are known to provide a more accurate depiction of the evolutionary relationships among a given group of species than trees based on a single gene/protein sequence [30,39,47,90,91]. Hence, a phylogenetic tree for the genome-sequenced nematodes species was constructed in this work based on concatenated sequences of 17 conserved proteins. The tree shows a clear separation of the two main classes, i.e., *Chromadorea* and *Enoplea*, within the nematodes [5]. Recently, Smythe et al. [41] have also reported phylogenomic analysis of 108 nematodes using a conservative orthology inference strategy. Their analyses also indicated that the class *Enoplea* formed a sister taxon to the rest of the Nematoda [41]. In the phylogenetic trees constructed in this work as well as by Smythe et al. [41], species from a number of nematode genera viz. *Caenorhabditis, Ancyclostoma, Trichinella, Trichuris,* and *Brugia*, formed distinct clades supporting their expected close and specific groupings. However, in both these trees, the species from the superfamilies *Strongyloidea*, *Trichostrongyloidea,* and *Metastrongyloidea* were found to cluster closely together and exhibited polyphyletic branching within each other. Thus, the clades corresponding to these superfamilies are reliably discerned presently and their interrelationships are also not resolved.

However, the main focus of the present work was on species from the genus *Caenorhabditis*, which formed a strongly supported monophyletic clade in the tree. Our comparative genomic analysis was aimed at identifying molecular markers that are commonly and uniquely shared by the members of this genus or other larger clades of nematodes which included *Caenorhabditis.* These studies have identified for the first time 52 novel molecular markers (or synapomorphies) consisting of conserved signature indels (CSIs) in proteins involved in various biological processes, which are uniquely shared by either all available *Caenorhabditis* species or other higher taxa of nematodes encompassing this genus, provide novel and important tools for studying these organisms. It should be mentioned that Mitreva and coworkers [43,92,93] have previously carried out extensive work examining the presence of indels in nematode proteins. Although their work has identified large numbers of indels in nematode proteins, unlike the CSIs that are the focus of this work, the indels identified by these authors are not specific for a phylogenetically coherent group (i.e., species related by common ancestry), and in most cases, they were also not present in conserved regions. Extensive earlier work shows that only the indels of fixed lengths, which are flanked on both sides by conserved regions and are uniquely found in a monophyletic group of organisms, provide reliable molecular characteristics that are useful for evolutionary studies and for the demarcation of different groups of organisms in molecular terms [37,46,47,48,49,50,94]. The other indels in protein sequences not meeting these criteria, although they provide valuable tools for genetic and biochemical studies [43,92], their utility for evolutionary studies is limited. 

**Figure 7:**
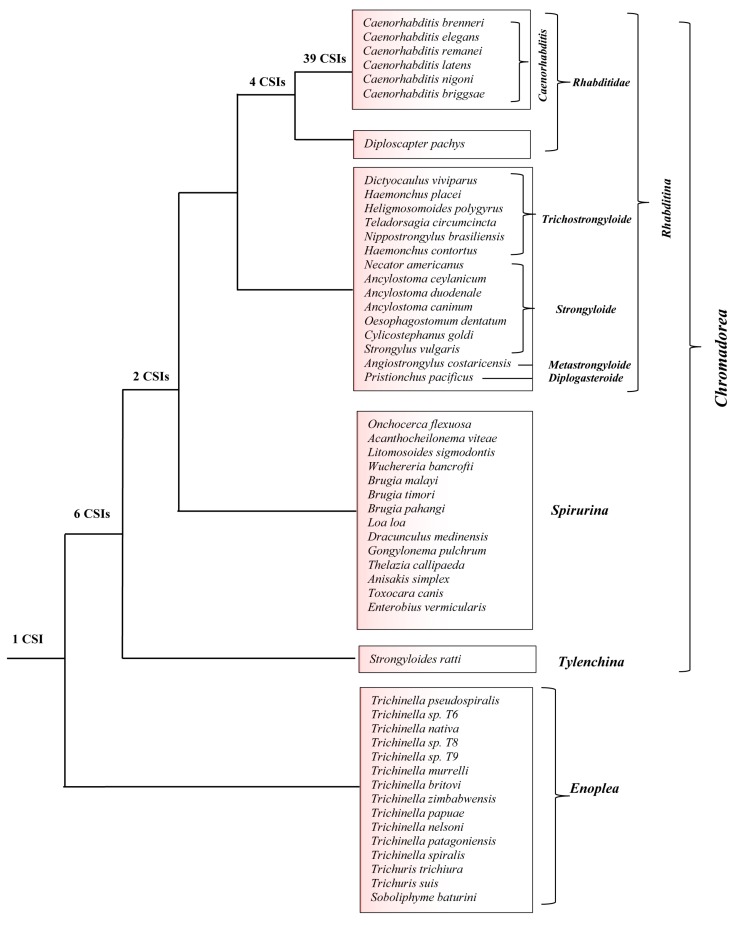
A conceptual diagram summarizing the species specificities of different nematodes-specific CSIs identified in this work and the evolutionary relationships inferred from them and the constructed phylogenetic tree. The numbers of CSIs that are specific for different clades or species-groupings are noted on the respective nodes.

A summary diagram showing the nematode groups’ specificities of different identified CSIs is presented in Figure 7. Of the 52 CSIs identified in this work, 39 CSIs in different proteins are uniquely shared by all members of the genus *Caenorhabditis*. Four CSIs are specific for the family *Rhabditoidea*, which, in addition to the genus *Caenorhabditis,* also includes the genome-sequenced species *Diploscapter pachys* whereas eight CSIs in unrelated proteins are distinguishing characteristics of the different species from the class *Chromadorea*. In addition, we have identified one CSI in an Na(+)/H(+) exchange regulatory factor, NRFL-1, that appears to be a common and unique characteristic of different species from the phylum Nematoda. Some molecular features specific for the phylum Nematoda have also been reported by Yin et al. [95]. However, our analysis did not identify any CSI that was specific for the *Strongyloidea* or *Trichostrongyloidea* superfamily, which also did not form well-resolved clades in our phylogenetic tree. Thus, the species distribution of the identified CSIs independently supports the different observed groupings of *Chromadorea* species in the phylogenetic tree. The specificities of the identified CSIs for different members of the indicated clades indicates that the genetic changes responsible for these CSIs initially occurred in the common ancestors of these groups and these genetic changes were then retained/inherited by various descendent species [47]. 

The identified CSIs, due to their exclusive presence in the indicated groups of nematodes, provide novel and useful means for the identification of both known as well as novel species from these groups in molecular terms and for genetic, biochemical and evolutionary relationships. Extensive earlier work on CSIs for other groups of organisms strongly indicates that these molecular characteristics exhibit a high degree of constancy and predictive ability to be found in other members of the indicated groups [37,45,47,96]. It is expected that of the 39 CSIs identified in the present work which are specific for *Caenorhabditis* species, a large number of them should also be found in other non-genome sequenced or novel *Caenorhabditis* species. All of the described CSIs are present within conserved regions of the genes/proteins. Thus, based on the conserved regions encompassing these CSIs, the presence/absence of these CSIs in other nematodes/*Caenorhabditis* species could be readily examined by means of different commonly used experimental techniques viz. PCR-based, q-PCR-based, as well as by in silico BLAST searches examining the presence of these CSIs in genomic sequence data. The CSIs-based approaches have been used previously for developing novel and highly specific diagnostic tests for a number of important bacterial pathogens [97,98].

The CSIs identified in this work are present in diverse proteins (see Table 1 and Table 2) that are involved in important/essential functions in *C. elegans* that are likely to be conserved in other nematodes as well. Although the cellular functions of these CSIs are currently not known, earlier work on CSIs in other organisms has shown that these conserved molecular characteristics play important and often essential functions in the organisms where they are found [84,99]. Most of the studied CSIs in protein sequences are located in the surface loops of proteins, which are known to play important roles in mediating novel protein–protein or protein–ligand interactions that are essential or important for the CSI-containing organisms [84,87,99]. In the present work, using homology modeling technique and structural overlaps of the CSIs-containing and CSIs-lacking proteins, we have mapped the locations of the CSIs in three proteins viz. Rab-44 (4R79.2), poly ADP-ribose glycohydrolase (PARG-1) and tRNA (guanine-N(1)-)-methyltransferase (F46F11.10) that contain CSIs specific for the *Caenorhabditis* or *Chormadorea* species (Figure 2 and Figure 4). In all three cases, the CSIs in these proteins in the modeled structures from *C. elegans* were localized in the surface-exposed regions of the proteins (Figure 6). 

As noted in the introduction, *C. elegans* is an important model organism for studying developmental process, for aging research, and for examining the cellular functions of different genes/proteins in eukaryotic organisms [2,3,22,26]. Further, as many genes in *C. elegans* (*Caenorhabditis* species) are homologous to human proteins, it has also used as a model for studying the role of homologous genes/proteins involved in human diseases [14,15,17,23,24,100]. One important advantage of *C. elegans* is that, in addition to its ease of growth, transparency, and well-studied developmental pathways, it can also be readily manipulated genetically. Thus, it should be possible to investigate in this system the functional significance of the CSIs that are specific for nematode groups [18,19,23,89,100,101,102,103]. Earlier work on CSIs has shown that these genetic characteristics are functionally important and often play essential roles in the organisms for which they are specific [84,94,99]. Additionally, the conserved indels in protein sequences also provide potential drug targets [92,104]. In view of these considerations, further studies on understanding the functional significance of the CSIs which are specific for the *Caenorhabditis*/nematodes species should be of much interest and these could lead to the discovery of novel functional aspects of these important organisms.

## Figures and Tables

**Table 1:** Characteristics of the CSIs specific for the Genus *Caenorhabditis.*

Protein Name	*C. elegans* Gene Name	Accession No.	Figure No.	Indel Size	Indel Position
Rab44	4R79.2	AFP33163	Figure 2A, Figure S2A	1 aa ins	233–263
Poly ADP-ribose Glycohydrolase	parg-1	NP_001255324	Figure 2B, Figure S2B	5 aa ins	411–454
Poly (ADP-ribose) polymerase 2	parp-2	NP_001022057	Figure S3	2 aa del	389–420
DnaJ-domain containing chaperone protein	dnj-16	OZF80352	Figure S4	1 aa del	186–207
Cyclin-dependent kinase 12	cdk-12	NP_001254914	Figure S5	1 aa del	456–487
CRAL-TRIO domain-containing Sec14 protein	T23G5.2	NP_001040875	Figure S6	2 aa ins	448–487
Mammalian ZAK kinase homolog	zak-1	NP_001254942	Figure S7	1 aa ins	80–109
Probable 3',5'-cyclic phosphodiesterase	pde-2	NP_001022706	Figure S8	2 aa ins	448–495
Nuclear Hormone Receptor	nhr-68	NP_001256335	Figure S9	1 aa del	1–35
SMA2- like	sma-1	NP_001256383	Figure S10	2 aa ins	1353–1393
Glutathione transferase omega-1 *	C02D5.4	NP_001254962	Figure S11	1 aa ins	65–103
Probable 26S proteasome regulatory subunit	rpn-6.2	NP_001254973	Figure S12	1 aa ins	46–90
Serine/ Threonine protein phosphatase 2A Regulatory Subunit	pptr-2	NP_001256283	Figure S13	1 aa ins	92–130
Failed axon connections-like protein *	F53G12.9	NP_001293265	Figure S14	1 aa ins	176–211
NADH dehydrogenase [ubiquinone] 1 alpha subcomplex assembly factor 2	Y116A8C.30	XP_002632399	Figure S15	13 aa ins	62–97
Disorganized muscle protein 1	Cbn-dim-1	EGT45899	Figure S16	1 aa del	135–170
ETS (E26 transformation-specific) class transcription factor	ets-9	NP_001024482	Figure S17	1 aa ins	54–78
Glycine-rich domain-containing protein	F32B5.7	EGT38541	Figure S18	1 aa ins	430–466
Heat shock protein 70	F11F1.1	NP_001255199	Figure S19	2 aa del	364–399
Heat shock protein 70	F11F1.1	NP_001255199	Figure S20	1 aa del	437–481
Abnormal cell migration protein 13	mig-13	NP_001024661	Figure S21	1 aa del	123–151
Regulatory-associated protein of mTOR-like protein	daf-15	XP_003089575	Figure S22	1 aa ins	143–175
Abnormal cell migration protein 13	mig-13	NP_001024661	Figure S23	3 aa del	141–170
Abnormal cell migration protein 13	mig-13	NP_001024660	Figure S24	1 aa del	220–251
Plexin	plx-1	NP_500018	Figure S25	1 aa ins	1460–1497
Piwi-like protein *	ergo-1	NP_503362	Figure S26	1 aa ins	1020–1070
Stomatin *	sto-1	NP_001123124	Figure S27	1 aa del	70–99
Ral guanine nucleotide dissociation stimulator	rgl-1	NP_001123140	Figure S28	1 aa del	257–290
Transglutaminase/ protease homolog	ltd-1	NP_001309573	Figure S29	1 aa del	261–290
Vacuolar protein sorting-associated protein 41 homolog	vps-41	NP_001033544	Figure S30	1 aa ins	209–242
Serine/arginine-rich splicing factor	rsp-1	NP_001317731	Figure S31	1 aa del	13–36
Serine/ Threonine-protein phosphatase PP1	Cni-W03D8.2	PIC40784	Figure S32	1 aa ins	159–191
NEPrilysin metallopeptidase *	nep-20	NP_001317749	Figure S33	1 aa del	761–804
DNA PRImase homolog	pri-2	NP_001251923	Figure S34	1 aa ins	224–262
Probable maleylacetoacetate isomerase	Y105E8A.21	NP_001252372	Figure S35	3 aa del	56–91
Glutathione S-transferase *	C25H3.7	NP_001254102	Figure S36	1 aa ins	39–61
CTD nuclear envelope phosphatase 1 homolog	cnep-1	NP_001254124	Figure S37	1 aa ins	32–52
Kelch-domain protein	F53E4.1	NP_506895	Figure S38	6 aa ins	206–248
Intermediate filament protein *	ifc-2	NP_741705	Figure S39	2 aa del	946-983

* Two isoforms of this protein are present in *Caenorhabditis* species.

**Table 2:** Characteristics of the CSIs specific for the nematode suborder *Rhabditoidea* and class *Chromadorea.*

Protein Name	*C. elegans* Gene Name	Accession No.	Figure (Fig. Sup) No.	Indel Size	Indel Position	Specificity
Cleavage Factor I_m_ homolog	cfim-2	NP_001255355	Figure S40	2 aa ins	87–130	*Rhabditoidea*
Methyl-CpG-binding protein	mbd-2	NP_001021012	Figure S41	2 aa ins	158–200	
Abnormal cell migration protein 13	mig-13	NP_001024660	Figure 3 Figure S42	2 aa ins	71–105	
PAX3- and PAX7 binding protein 1	F43G9.12	NP_001250840	Figure S43	1 aa del	126–164	
tRNA (guanine-N(1)-)-methyltransferase	F46F11.10	NP_491647	Figure 4	4 aa ins	632–669	*Chromadorea*
Palmitoyltransferase ^a^	spe-10	KHJ83757	Figure S44	1 aa del	234–270	
Palmitoyltransferase	spe-10	KHJ83757	Figure S45	2 aa del	255–282	
Battenin	cln-3.3	EGT30700	Figure S46	3 aa ins	162–194	
ETS (E26 transformation-specific) class transcription factor	ets-5	KJH47557	Figure S47	1 aa ins	122–155	
Heterogeneous nuclear ribonucleoprotein A1 *	H28G03.1	KJH46562	Figure S48	1 aa ins	93–122	
Heterogeneous nuclear ribonucleoprotein A1 *	H28G03.1	XP_013302959	Figure S49	5 aa del	139–171	
Regulator of G-protein signaling 7 ^a^	Cbn-rgs-7	EGT30339	Figure S50	1 aa ins	221–252	
Na(+)/H(+) Exchange Regulatory Factor *	nrfl-1	NP_001294068	Figure 5 Figure S51	1 aa ins	210–245	Nematoda

* Two isoforms of this protein are present in *Rhabitida* species. ^a^ These CSIs are not found in *Strongyloides ratti*, which branches deeply in comparison to the other *Chromadorea species*.

## Supplementary Materials

The following are available online at https://www.mdpi.com/2073-4425/10/10/739/s1, Table S1: Name and accession numbers of proteins used for phylogenetic analysis, Figure S1: Maximum-likelihood trees for genome-sequenced nematode species based on concatenated sequences of 17 conserved proteins. The trees were constructed in MEGA6 using (A) Whelan and Goldman + Freq. and (B) JTT matrix-based models, Figure S2: Partial sequence alignments of (A) Rab-44 protein containing a 1 aa CSI and (B) poly ADP-ribose glycohydrolase protein containing a 5 aa insertion, which re specific for the genus *Caenorhabditis*, Figure S3: Partial sequence alignments of poly (ADP-ribose) polymerase 2 protein with 2 aa deletion which is specific for the *Caenorhabditis* genus, Figure S4: Partial sequence alignments of DnaJ-domain containing chaperone protein consisting of a 1 aa deletion which is specific for the *Caenorhabditis* genus, Figure S5: Partial sequence alignments of Cyclin dependent kinase 12 protein consisting of a 1 aa deletion that is specific for the *Caenorhabditis* genus, Figure S6: Partial sequence alignments of CRAL-TRIO domaincontaining Sec14 protein consisting of a 2 aa CSI which is specific for the *Caenorhabditis* genus, Figure S7: Partial sequence alignments of mammalian ZAK kinase homolog protein with 1 aa CSI which is specific for the *Caenorhabditis* genus, Figure S8: Partial sequence alignments of probable 3′,5′-cyclic phosphodiesterase pde-2 protein with a 2 aa CSI which is specific for the *Caenorhabditis* genus, Figure S9: Partial sequence alignments of nuclear hormone receptor protein with 1 aa deletion which is specific for the *Caenorhabditis* genus, Figure S10: Partial sequence alignments of SMAII-like (spore membrane assembly protein 2-like) protein with 2 aa CSI which is specific for the *Caenorhabditis* genus, Figure S11: Partial sequence alignments of glutathione transferase omega-1 protein with a 1 aa CSI which is specific for the *Caenorhabditis* genus, Figure S12: Partial sequence alignments of 26S proteasome regulatory protein subunit with a CSI consisting of a 1 aa CSI which is specific for the *Caenorhabditis* genus, Figure S13: Partial sequence alignments of serine/threonine protein phosphatase 2A regulatory protein subunit with a 1 aa CSI which is specific for the *Caenorhabditis* genus, Figure S14: Partial sequence alignments of failed axon connections like protein with a 1 aa CSI which is specific for the *Caenorhabditis* genus, Figure S15: Partial sequence alignments of NADH dehydrogenase 1 alpha sub-complex assembly factor 2 protein with a 13 aa CSI which is specific for the *Caenorhabditis* genus, Figure S16: Partial sequence alignments of disorganized muscle protein with a 1 aa deletion that is specific for the *Caenorhabditis* genus, Figure S17: Partial sequence alignments of ETS (E26 transformation-specific) class transcription factor protein with a 1 aa CSI which is specific for the *Caenorhabditis* genus, Figure S18: Partial sequence alignments of glycine-rich domain containing protein with a 1 aa CSI which is specific for the *Caenorhabditis* genus, Figure S19: Partial sequence alignments of a feat shock protein 70 protein consisting of a 2 aa deletion that is specific for the *Caenorhabditis* genus, Figure S20: Partial sequence alignments of a heat shock protein 70 protein consisting of a 1 aa deletion which is specific for the *Caenorhabditis* genus, Figure S21: Partial sequence alignments of abnormal cell migration protein 13 protein with a 1 aa deletion which is specific for the *Caenorhabditis* genus, Figure S22: Partial sequence alignments of regulatory associated protein of mTOR-like protein consisting of a 1 aa CSI that is specific for the *Caenorhabditis* genus, Figure S23: Partial sequence alignments of abnormal cell migration protein 13 protein consisting of a 3 aa deletion which is specific for the *Caenorhabditis* genus, Figure S24: Partial sequence alignments of abnormal cell migration protein 13 protein consisting of a 1 aa deletion which is specific for the *Caenorhabditis* genus, Figure S25: Partial sequence alignments of Plexin protein with a 1 aa CSI that is specific for the *Caenorhabditis* genus, Figure S26: Partial sequence alignments of a Piwi-like protein protein consisting of a 1 aa CSI that is specific for the *Caenorhabditis* genus, Figure S27: Partial sequence alignments of stomatin protein with a CSI consisting of a 1 aa deletion that is specific for the *Caenorhabditis* genus, Figure S28: Partial sequence alignments of Ral guanine nucleotide dissociation stimulator protein consisting of a 1 aa deletion that is specific for the *Caenorhabditis* genus, Figure S29: Partial sequence alignments of transglutaminase/protease homolog protein consisting of a 1 aa deletion that is specific for the *Caenorhabditis* genus, Figure S30: Partial sequence alignments of vacuolar protein sorting associated protein 41 homolog protein consisting of a 1 aa CSI which is specific for the *Caenorhabditis* genus, Figure S31: Partial sequence alignments of serine/arginine-rich splicing factor protein consisting of a 1 aa deletion that is specific for the *Caenorhabditis* genus, Figure S32: Partial sequence alignments of serine/threonineprotein phosphatase protein consisting of a 1 aa CSI that is specific for the *Caenorhabditis* genus, Figure S33: Partial sequence alignments of NEPrilysin metallopeptidase family protein consisting of a 1 aa deletion which is specific for the *Caenorhabditis* genus, Figure S34: Partial sequence alignments of DNA PRImase homolog protein consisting of a 1 aa CSI which is specific for the *Caenorhabditis* genus, Figure S35: Partial sequence alignments of a probable maleylacetoacetate isomerase protein with a 3 aa deletion that is specific for the *Caenorhabditis* genus, Figure S36: Partial sequence alignments of glutathione S-transferase protein consisting of a 1 aa CSI which is specific for the *Caenorhabditis* genus, Figure S37: Partial sequence alignments of CTD nuclear envelope phosphatase 1 homolog protein with a 1 aa CSI which is specific for the *Caenorhabditis* genus, Figure S38: Partial sequence alignments of Kelch-domain protein with a 6 aa CSI that is specific for the *Caenorhabditis* genus, Figure S39: Partial sequence alignments of intermediate filament protein consisting of a 2 aa deletion that is specific for the *Caenorhabditis* genus, Figure S40: Partial sequence alignments of cleavage factor IM (CFIm) homolog protein consisting of a 2 aa CSI which is specific for the *Rhabditoidea* suborder, Figure S41: Partial sequence alignments of Methyl-CpG-binding protein consisting of a 2 aa CSI which is specific for the *Rhabditoidea* suborder, Figure S42: Partial sequence alignments of abnormal cell migration protein 13 consisting of a 1 aa CSI which is specific for the *Rhabditoidea* suborder, Figure S43: Partial sequence alignments of PAX3- and PAX7 binding protein 1 protein with a 1 aa deletion that is specific for the *Rhabditoidea* suborder, Figure S44: Partial sequence alignments of palmitoyltransferase protein consisting of a 1 aa deletion that is specific for the *Chromadorea* class, Figure S45: Partial sequence alignments of palmitoyltransferase protein with a 2 aa deletion that is specific for the *Chromadorea* class, Figure S46: Partial sequence alignments of a battenin protein with a 3 aa CSI that is specific for the *Chromadorea* class, Figure S47: Partial sequence alignments of ETS (E26 transformation-specific) class transcription factor protein consisting of a 1 aa CSI that is specific for the *Chromadorea* class, Figure S48: Partial sequence alignments of heterogeneous nuclear ribonucleoprotein A1 protein with a 1 aa CSI that is specific for the *Chromadorea* class, Figure S49: Partial sequence alignments of heterogeneous nuclear ribonucleoprotein A1 protein consisting of a 5 aa deletion which is specific for the *Chromadorea* class, Figure S50: Partial sequence alignments of regulator of Gprotein signaling 7 protein consisting of a 1 aa CSI which is specific for the *Chromadorea* class, Figure S51: Partial sequence alignments of Na(+)/H(+) exchange regulatory factor protein with a 1 aa CSI which is specific for the entire Nematoda phylum.
