# Supplementary material for: Genomic Analyses Identify Novel Molecular Signatures Specific for the Caenorhabditis and other Nematode Taxa Providing Novel Means for Genetic and Biochemical Studies
Source: Genes (Basel). 2019 Sep 24;10(10):739. doi: 10.3390/genes10100739 (PMC6826867; doi:10.3390/genes10100739)
Supplement: Supplementary file 1 [file genes-10-00739-s001.pdf]

Table S1

Name and Accession Numbers of Proteins Used for Phylogenetic Analysis

Protein Name	Accession Number	Protein Length
Protein CBG25184	CAS00939	1573 aa
CBR-GDI-1 protein	XP_002632610	444
CBR-SRI-18 protein	XP_002637071	331
CBR-PQN-68 protein	XP_002637004	150
CBR-PRS-1 protein	XP_002643184	579
Hypothetical protein CBG16343	XP_002643609	519
CBR-EPS-8 protein	XP_002632592	918
Hypothetical protein CBG03002	XP_002631206	361
CBR-ENOL-1 protein	XP_002631372	434
CBR-GTA-1 protein	XP_002633716	483
Hypothetical protein CBG04561	XP_002637778	326
CBR-LFE-2 protein	XP_002639970	393
CBR-OBR-1 protein	XP_002641357	762
Hypothetical protein CBG18198	XP_002642224	314
CBR-VPS-41 protein	XP_002644672	898
CBR-RPL-13 protein	XP_002646297	207
CBR-UNC-73 protein	XP_002646360	1618

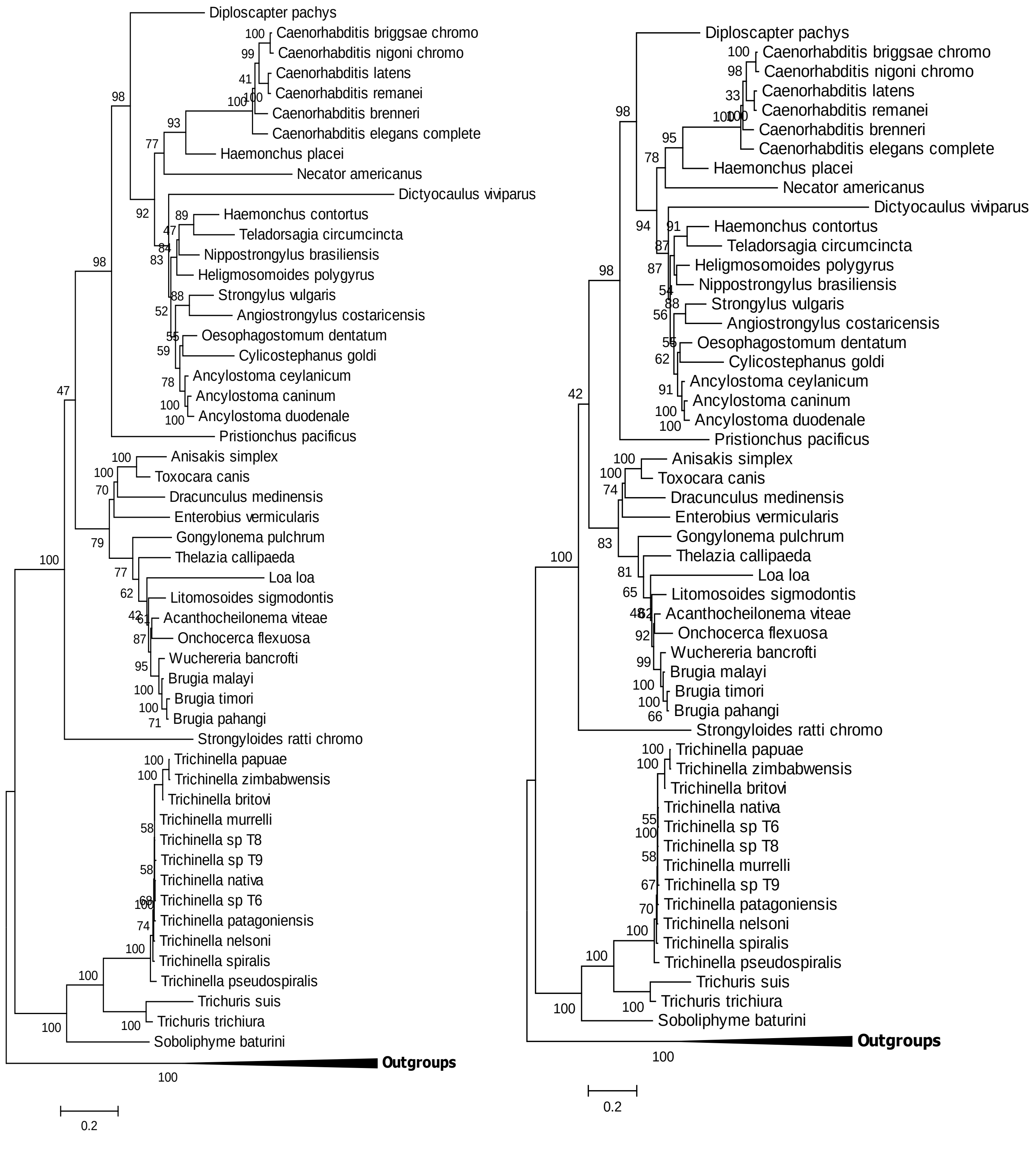

Figure S1. Maximum Likelihood phylogenetic trees of the genome sequenced nematode species based on concatenated sequences of 17 conserved proteins. The trees were constructed in MEGA6 using (A) Whelan and Goldman + Freq. and (B) JTT matrix-based models. Trees are based on 100 bootstrap replicates and rooted using sequences for Apicomplexa species as in Figure 1.

Group	Species	Accession	233		263
Caenorhabditis (6/6)	*Caenorhabditis elegans*	AFP33163	TIGVDFTVKTMKIP	P	NRAIAMQLWDTAGQER
	Caenorhabditis brenneri	EGT41967	--------------	-	----------------
	Caenorhabditis remanei	OZF92029	--------------	-	----------------
	Caenorhabditis latens	OZF87495	--------------	-	----------------
	Caenorhabditis briggsae	CAP21883	-------------A	-	----------------
	Caenorhabditis nigoni	PIC31209	-------------A	-	----------------
Other Nematodes (0/48)	*Toxocara canis*	KHN75949	----------IRLQ		--VV-V----------
	Nippostrongylus brasiliensis	VDL82832	------MI--V-VD		-DK-KL-I--------
	Gongylonema pulchrum	VDN36140	------MI--V-VN		DDK-KL-I--------
	Oesophagostomum dentatum	KHJ84531	----------I-VG		--VV-L----------
	Wuchereria bancrofti	EJW79243	----------IRLC		--VV-I----------
	Ancylostoma duodenale	KIH53884	----------I-VG		S-VV-L----------
	Brugia pahangi	VDN91210	----------IRLC		--VV-V----------
	Strongylus vulgaris	VDM82877	----------I-VG		S-VV-L----------
	Brugia malayi	CRZ23819	----------IRLC		--VV-V----------
	Brugia timori	VDO25812	----------IRLC		--VV-V----------
	Necator americanus	XP_013308986	----------I-VG		S-VV-L----------
	Dictyocaulus viviparus	KJH46195	-----------TVG		D-VV-L----------
	Enterobius vermicularis	VDD95818	----------I-VK		DKFV-V----------
	Ancylostoma caninum	RCN39681	----------I-VG		S-VV-L----------
	Dracunculus medinensis	VDN57676	---------NIRLG		--TV-V----------
	Heligmosomoides polygyrus	VDO22485	----------ITVG		D-VV-L----------
	Ancylostoma ceylanicum	EYC11435	----------I-VG		S-IV-L----------
	Angiostrongylus costaricensis	VDM58536	----------ICVG		--VV-L----------
	Heterodera cruciferae	AMD33341	----------LLVR		E-MV-L----------
	Haemonchus placei	VDO62950	----------ITVG		E-VV-L----------
	Acanthocheilonema viteae	VBB25686	-------I--IRLC		D-IV-V----------
	Onchocerca flexuosa	OZC09065	---I--I---IHLY		--VV-V----------
	Diploscapter pachys	PAV91080	----------VQLK		G-F--L----------
	Haemonchus contortus	CDJ88358	----------ITVG		E-VV-L----------
	Onchocerca ochengi	VDM94142	------I---IRLY		D-VV-V----------
	Strongyloides ratti	XP_024499704	----------IN-N		DKKVTI----------
	Pristionchus pacificus	PDM64254	----------VQLR		D-LL-V----------
	Cylicostephanus goldi	VDK82117	---I--LS---YLE		D-TVRL----------
	Loa loa	XP_003145161	------MI--V-VN		DDK-KL-I--------
	Anisakis simplex	VDK63524	------MI--V-VG		DDK-KL-I--------
	Soboliphyme baturini	VDP03528	-------M--LI-D		SKRVKL-I---G----
	Litomosoides sigmodontis	VDK79798	------MI--V-VN		EDK-KL-I--------
	Teladorsagia circumcincta	PIO59286	------KIR-VD-D		GQRVKL-I--------
	Thelazia callipaeda	VDM96437	-----Y----FHLD		HCVV-L----------
	Trichinella nelsoni	KRX14869	---I--HM-IL-VE		DQL--L----------
	Trichinella nativa	KRZ52102	---I--HM-IL-VE		DQL--L----------
	Trichinella sp. T6	KRX81729	---I--HM-IL-VE		DQL--L----------
	Trichinella pseudospiralis	KRY72075	---I--HM-IL-VE		DQL--L----------
	Trichinella spiralis	KRY29793	---I--HM-IL-VE		DQL--L----------
	Trichinella papuae	KRZ80298	---I--HM-IL-VE		DQL--L----------
	Trichinella britovi	KRY53573	---I--HM-IL-VE		DQL--L----------
	Trichinella zimbabwensis	KRZ14659	---I--HM-IL-VE		DQL--L----------
	Trichinella patagoniensis	KRY13850	---I--HM-IL-VE		DQL--L----------
	Trichuris suis	KHJ43420	---I--HLRVL-VE		DQL--L----------
	Trichuris trichiura	CDW52341	---I--LS---YLE		D-TVRL----------
Outgroups (0/>100)	*Homo sapiens*	NP_001138430	-V-I-YR---LNVD		-SQV-L----------
	Mus musculus	XP_006506407	-V-I-YR---VTVD		-AQV-L----------
	Babesia bovis	BAN65758	------KI--V--D		-VT-KL-I--------
	Hydra vulgaris	XP_012556792	------Q---LE-C		-NW--L----------

Supplementary Figure 2A. Partial sequence alignments of the protein Rab-44 with a CSI consisting of a 1 aa insertion (shown in black box) that is specific for the *Caenorhabditis* genus and not present in any other nematode species. *T. canis, G. pulchrum* and *N. brasiliensis* contains second homologs which contain this CSI.

			411		454
Caenorhabditis (6/6)	*Caenorhabditis elegans*	NP_001023137	FSSYTGYGHTLKWAELQPNHS	RQNTN	EFRDRFGRLRVETIAIDA
	Caenorhabditis remanei	XP_003093608	Y---D---KS---KP---EDA	D--DS	SL--KY---P--------
	Caenorhabditis brenneri	EGT38432	----D---TST--RP-K-K-A	---EP	SN--KY------------
	Caenorhabditis briggsae	XP_002647141	Y-G----ANK---RP-SGRYA	---DA	RL--KY-------V----
	Caenorhabditis nigoni	PIC17744	Y-G----ARD---HP-T-RYA	---DT	RL--KY-------T----
	Caenorhabditis latens	OZG17769	PKPKPAPRK----KA---EDA	D--DT	SL--KY---P--------
Other Nematodes (0/21)	*Diploscapter pachys*	PAV64174	----S------N-KQYDNFGK	E	KT-NE---INNIM-----
	Gongylonema pulchrum	VDK27510	--------P-FR-QRMEHED-		TP-------LC-LV----
	Haemonchus contortus	CDJ80847	Y---F---GS-Q--PFEDFDR		-P--K---V-C-LV-M--
	Onchocerca ochengi	VDK68310	--D-D---SSFR-RPMKKM--		FP----N--CC-LV----
	Onchocerca flexuosa	OZC09105	--D-D---SSFR-RPMKKM--		FP----N--CC-LV----
	Dictyocaulus viviparus	KJH49243	H---I---E--Q--PYEEYG-		-S--K-C-VKC-VV-M--
	Toxocara canis	VDM38707	Y-D-E---DSFQ-YP-HSPDP		LS----E---C-MV----
	Brugia timori	VDO13221	--D-N---SSFR-RPMEKMD-		FP----N--CC-LV----
	Brugia malayi	XP_001897754	--D-N---SSFR-RPMEKMD-		FP----N--CC-LV----
	Anisakis simplex	VDK26367	Y-D-A---NSFQ-YP-HAPEA		LS----E--HC-LV----
	Brugia pahangi	VDN90162	--D-N---SSFR-RPMEKMD-		FP----N--CC-LV----
	Oesophagostomum dentatum	KHJ76834	Y-D-I---E--E-SP-ENYGD		-P--E-R-VIC-VV-M--
	Ancylostoma duodenale	KIH62945	Y-D-----E--E--R-ENYGF		-P--C-R-VIC-VV-M--
	Ancylostoma ceylanicum	EPB77283	Y-D-----E--E--R-ENYGF		-P--C-R-VIC-VV-M--
	Wuchereria bancrofti	EJW76497	--D-N---PSFR-RPMEKVD-		FP----N--CC-LV----
	Ancylostoma caninum	RCN39092	Y---S---K----LPFEGYG-		-P--E-R-VICNVV-M--
	Thelazia callipaeda	VDN02538	--N-V---SSFR-CPMEIKNP		LP----R--CC-LV-M--
	Necator americanus	XP_013299999	Y-D-F---E--E-NQ-EDYG-		-P--E-L-VIS-VV-M--
	Loa loa	XP_020303580	--G-D---SSFR-RPMEKMD-		FP----H--CC-LV----
	Litomosoides sigmodontis	VDK84342	--D-S---SSFR-RPRRKVD-		FP----N--CC-LV----
	Acanthocheilonema viteae	VBB29553	--D-S---SSFC-RPMVKAD-		FP----N--CC-LV----
Outgroups (0/>200)	*Homo sapiens*	NP_001290415	Y-E----AE-YR-SRSHEDG-		E--DWQ-RCT-IV----
	Mus musculus	NP_001346844	Y-E----AE-YR--RSHEDG-		EK-DWQ-RCT-IV----
	Pocillopora damicornis	RMX52245	Y-D----A--FR--GNHSDRT		T--SW--RHSKIV----
	Exaiptasia pallida	XP_020892460	Y-D----A--F---GNHDDQT		P--SW--RHCQVV----
	Stylophora pistillata	PFX34124	Y-D----A--FR--GNHSDRT		T--SW--RHSKIV----
	Acropora millepora	XP_029187629	--D-E--A--FR--GNHVDQV		T--SW--RCCSIV----
	Seriola dumerili	XP_022625279	--V-S-F-DSFE--GPHQDRL		D--EW---KRQIL----
	Cyprinodon variegatus	XP_015238624	Y-K----SQ-Y---GSHQDTT		P--DW--RCT-IV----
	Seriola lalandi dorsalis	XP_023268207	--V-S-F-DSF---GPHRD-L		D--GW---KRQIL----
	Danaus plexippus plexippus	OWR43927	Y-T-S----SFS-GSNYNDIT		P--SS--K-TAVL----
	Notothenia coriiceps	XP_010772468	--N-S-F-D-FQ-KGPHEERL		N--EWA--QRQIV----
	Xenopus tropicalis	XP_017950760	Y-E----SE-Y---RVHEDE-		P--EWQ-RTT-IV----
	Bicyclus anynana	XP_023940579	--T------SFQ--GDFADET		PY-SS--R-CAVL----
	Perca flavescens	XP_028456875	--L-S---E-FE--GPYEDRL		D--EWA--KRQIL----
	Blyttiomyces helicus	RKO90865	--D-E---D-F--KGPHEDLA		P--ALC--KT-I--V--
	Vanessa tameamea	XP_026483100	Y-K-S----SFQ--GEHVDAT		P--SS--R-CAVL----
	Myotis lucifugus	XP_023617070	Y-E----AE-YR--RSHEDGN		E--DWQ-RST-IV----
	Myotis brandtii	XP_014386202	Y-E----AE-YR--RSHEDGN		E--DWQ-RST-IV----
	Mizuhopecten yessoensis	XP_021376375	--D-D--AR-FR-K-NYEDKT		P--NW---CTDVV----
	Acanthochromis polyacanthus	XP_022064719	--C-S-F-DSFE--GPHEE-L		K--AWA--KRQIL----
	Orbicella faveolata	XP_020608915	Y-D-I--A--FR--GDHVDET		T--SW--RHSKIV----
	Xenopus laevis	NP_001089602	Y-E----SE-Y---CVHEDE-		P--EWQ-RTT-IV----

Supplementary Figure 2B. Partial sequence alignments of a Poly ADP-ribose Glycohydrolase protein with a CSI consisting of a 5 aa insertion (shown in black box) that is specific for the *Caenorhabditis* genus and not present in any other nematode species.

Group	Species	Accession	389		420
Caenorhabditis (5/5)	Caenorhabditis elegans	NP_001022057	RVTNVFSILMNGL		QFPVGDRCGLMFGNGVYFA
	Caenorhabditis briggsae	XP_002629728	-------------		-M-E---------------
	Caenorhabditis nigoni	PIC43332	-------------		-M-E---------------
	Caenorhabditis brenneri	EGT38253	-------------		-M-E---------------
	Caenorhabditis remanei	XP_003097265	-------------		-M-E---Y-----------
Other Nematodes (0/26)	Loa loa	XP_003143505	-L--W----SQ--	RI	AP-EAPTT-Y---K-I---
	Onchocerca ochengi	VDN00454	-L--WY---SQ--	RI	AP-EAPVS-Y---K-I---
	Wuchereria bancrofti	VDM21799	-L--WY---SQ--	RI	AP-EAPAT-Y---K-I---
	Pristionchus pacificus	PDM64980	-LS-F-G--SQ--	RI	AP-EAPST-Y---K-----
	Diploscapter pachys	PAV91078	-LA-W----TQ--	RI	AP-EAPMT-Y---K-I---
	Enterobius vermicularis	VDD85971	-I--WYG--SQ--	RV	AP-EAPST-F---K-I---
	Anisakis simplex	VDK59352	-L--WYG--SQ--	RI	AP-EAPST-Y---K-----
	Heligmosomoides polygyrus	VDO67729	--S-FYN--SH--	RI	AP-EVPLT-F---K-----
	Trichoplax adhaerens	XP_002109067	----FVG--SQ--	RI	AP-EAPVT-Y---K-----
	Soboliphyme baturini	VDP00762	-LV-WY---CS--	KI	AP-EAPST-Y---K-I---
	Brugia pahangi	VDN89517	-L--WY---SQ--	RI	APLEAPKT-Y---K-I---
	Brugia timori	VDO24680	-L--WY---SQ--	RI	APLEAPKT-Y---K-I---
	Brugia malayi	XP_001893054	-L--WY---SQ--	RI	APLEAPKT-Y---K-I---
	Schistosoma margrebowiei	VDO49046	-L--WVG--GR--	QI	AP-EAPSS-Y---K-----
	Schistosoma mattheei	VDP63272	-L--WVG--GR--	QI	AP-EAPSS-Y---K-----
	Nippostrongylus brasiliensis	VDL70778	--S-FYN--SH--	RI	AP-EVPLT-Y---K-----
	Trichuris suis	KHJ46115	-NS-YAG--CQ--	RI	AP-EAPVT-Y---K-I---
	Trichinella nelsoni	KRX19541	-LS-FVG-ISQ--	RI	AP-ESLTS-H---K-I---
	Trichinella patagoniensis	KRY16608	-LS-FVG-ISQ--	RI	AP-ESLTS-H---K-I---
	Trichinella murrelli	KRX42668	-LS-FVG-ISQ--	RI	AP-ESLTS-H---K-I---
	Trichinella spiralis	KRY39132	-LS-FVG-ISQ--	RI	AP-ESLTS-H---K-I---
	Trichinella sp. T8	KRZ84918	-LS-FVG-ISQ--	RI	AP-ESLTS-H---K-I---
	Trichinella britovi	KRY54119	-LS-FVG-ISQ--	RI	AP-ESLTS-H---K-I---
	Trichinella sp. T6	KRX77647	-LS-FVG-ISQ--	RI	AP-ESLTS-H---K-I---
	Trichinella nativa	OUC43219	-LS-FVG-ISQ--	RI	AP-ESLTS-H---K-I---
	Trichinella zimbabwensis	KRZ17851	-LS-FVG-ISQ--	RI	AP-ESLIS-H---K-I---
Outgroups (0/>100)	Homo sapiens	1UK0_A	T--FAG--SQ--	RI	AP-EAPVT-Y---K-I---
	Mus musculus	AAF61293	T--FAG--SQ--	RI	AP-EAPVT-Y---K-I---

Supplementary Figure 3. Partial sequence alignments of a Poly (ADP-ribose) polymerase 2 protein with a CSI consisting of a 2 aa deletion (shown in black box) that is specific for the *Caenorhabditis* genus and not present in any other nematode species. The sequence alignment of the species *Caenorhabditis latens* was not observed in the BLASTP search.

Group	Species	Accession	186 – 207
Caenorhabditis (6/ 6)	*Caenorhabditis remanei*	OZF80352	GVAIICKSS [ ] SSKFKLVLFDKEG
	Caenorhabditis nigoni	PIC39710	--------- [ ] -------------
	Caenorhabditis brenneri	EGT45655	--------- [ ] -------------
	Caenorhabditis latens	OZG18293	--------- [ ] -------------
	Caenorhabditis elegans	NP_001254891	--------- [ ] -------------
	Caenorhabditis briggsae	XP_002642541	--------- [ ] -------------
Other Nematodes (0/ 40)	*Diploscapter pachys*	PAV60060	-ISV--R-T S F---------RD-
	Teladorsagia circumcincta	PIO53971	-IC-V---A S -------------
	Dictyocaulus viviparus	KJH48665	-IC-V---P S -------------
	Angiostrongylus costaricensis	VDM62769	-IC-V---P S -------------
	Brugia timori	VDO44364	-LMV--R-A Q M------------
	Wuchereria bancrofti	EJW80584	-LMV--R-A Q M------------
	Brugia malayi	XP_001900908	-LMV--R-A Q M------------
	Pristionchus pacificus	PDM62666	--S-V---P S M---------RD-
	Anisakis simplex	VDK63706	-LM-----A Q M------------
	Dracunculus medinensis	VDN52079	-LM-M---P Q M------------
	Soboliphyme baturini	VDO93050	-IVLV-R-H S E----II------
	Onchocerca flexuosa	OZC07009	-LM---R-M Q M------------
	Onchocerca ochengi	VDK87080	-LM---R-M Q M------------
	Acanthocheilonema viteae	VBB26716	-LM---R-M Q M------------
	Enterobius vermicularis	VDD88357	-IM-----I Q M------------
	Loa loa	XP_020303998	-LMV--R-M Q M------------
	Litomosoides sigmodontis	VDK80743	-LMV--R-M Q M------------
	Thelazia callipaeda	VDN08491	-LM---R-F Q M------F-----
	Strongyloides ratti	XP_024507239	-IIVRVS-A S M-----II---D-
	Oesophagostomum dentatum	KHJ79345	-IC-V---- S -------------
	Haemonchus placei	VDO41032	-IC-V---- S -------------
	Haemonchus contortus	CDJ87271	-IC-V---- S -------------
	Heligmosomoides polygyrus	VDO90444	-IC-V---- S -------------
	Necator americanus	XP_013300303	-IC-V---- S -------------
	Nippostrongylus brasiliensis	VDL82817	-IC-V---- S -------------
	Gongylonema pulchrum	VDN22375	-LM---R-M Q M-------EMSKF
	Toxocara canis	VDM47619	-LM---R-A Q M----------D-
	Trichuris suis	KFD58118	-II-S-R-- S M---------RD-
	Trichinella nelsoni	KRX21170	-II-S-R-T D M-----I------
	Trichinella nativa	OUC45935	-II-S-R-T D M-----I------
	Trichinella pseudospiralis	KRY85173	-II-S-R-T D M-----I------
	Trichinella papuae	95459948	-II-S-R-T D M-----I------
	Trichinella zimbabwensis	KRZ11165	-II-S-R-T D M-----I------
	Trichinella patagoniensis	KRY22341	-II-S-R-T D M-----I------
	Trichinella sp. T6	KRX75581	-II-S-R-T D M-----I------
	Trichinella sp. T8	KRZ86851	-II-S-R-T D M-----I------
	Trichinella britovi	KRY49360	-II-S-R-T D M-----I------
	Trichinella spiralis	KRY33941	-II-S-R-T D M-----I------
	Trichinella murrelli	KRX41356	-II-S-R-T D M-----I------
	Trichuris trichiura	CDW57381	-II-S-R-- S M---------RD-

Supplementary Figure 4. Partial sequence alignments of a DnaJ-domain containing chaperone protein with a CSI consisting of a 1 aa deletion (shown in black box) that is specific for the *Caenorhabditis* genus and not present in any other nematode species. Homologous sequences for this protein were not observed for non-nematode organisms in BLASTP search.

			456 487
Caenorhabditis (6/6)	*Caenorhabditis elegans*	NP_001254914	GELKIADLGLARLW [ ] EKESRLYTNRVITLWYRP
	Caenorhabditis remanei	POM37359	-------------- [ ] Q-----------------
	Caenorhabditis latens	OZG10685	-------------- [ ] Q-----------------
	Caenorhabditis brenneri	EGT52238	-------------- [ ] Q-----------------
	Caenorhabditis nigoni	PIC37815	-------------- [ ] Q--------N--------
	Caenorhabditis briggsae	XP_002642141	-------------- [ ] Q--------N--------
Other Nematodes (0/43)	*Diploscapter pachys*	PAV78157	-------F----RF Y ADQQ-P------------
	Strongyloides ratti	XP_024506005	--I-L--F----FY N --QK--------------
	Necator americanus	XP_013295973	-------F----YY S SDQE--------------
	Haemonchus contortus	CDJ82163	-------F----YY S SDQE--------------
	Oesophagostomum dentatum	KHJ92341	-------F----YY S SDQE--------------
	Toxocara canis	KHN86651	--I-L--F-----Y D -EQD-P------------
	Nippostrongylus brasiliensis	VDL74284	-------F----YY S SDQE--------------
	Loa loa	XP_003140552	----L-------FY D -DQD-P------------
	Enterobius vermicularis	VDD93079	--I-L--F-----Y D -DQN-P------------
	Teladorsagia circumcincta	PIO63993	-------F----YY S SDQE--------------
	Strongylus vulgaris	VDM71588	-------F----YY S SDQE--------------
	Haemonchus placei	VDO22610	-------F----YY S SDQE--------------
	Dictyocaulus viviparus	KJH46797	--I----F----YY S SDQE--------------
	Brugia malayi	XP_001900704	----L--F----FY D -DQD-P------------
	Brugia pahangi	VDN89696	----L--F----FY D -DQD-P------------
	Litomosoides sigmodontis	VDK80595	----L--F----FY D -DQD-P------------
	Angiostrongylus costaricensis	VDM58212	--I----F----YY S SDQE--------------
	Onchocerca flexuosa	VDP12968	----L--F----FY D -DQD-P------------
	Dracunculus medinensis	VDN54878	--I-L--F-----Y D -ELD-P------------
	Acanthocheilonema viteae	VBB30047	----L--F----FY D -DQD-P------------
	Wuchereria bancrofti	EJW84954	----L--F----FY D -DQD-P------------
	Ancylostoma duodenale	KIH59324	-------F----YY S SDQE--------------
	Ancylostoma caninum	RCN31189	-------F----YY S SDQE--------------
	Thelazia callipaeda	VDN02201	----L--F----FY N -DQD-P------------
	Anisakis simplex	VDK58077	--I-L--F-----Y D -DQD-P------------
	Gongylonema pulchrum	VDN21708	----L--F----FY D -EQD-P------------
	Brugia timori	VDO13135	----L--F----FY D -DQD-P------------
	Trichuris suis	KFD49469	-DI-L--------Y Q RGQQ-P---K--------
	Cylicostephanus goldi	VDK59804	--I----F----YY S SDQE--------------
	Trichuris trichiura	CDW56806	-DI-L--------Y Q RGQQ-P---K--------
	Soboliphyme baturini	VDP09343	--I-L--F-----Y Q HDKE-P---K--------
	Trichinella pseudospiralis	KRX94481	--I-L--F-----Y Q RDKV-P---K--------
	Trichinella patagoniensis	KRY21320	--I-L--F-----Y Q RDKV-P---K--------
	Trichinella nelsoni	KRX27294	--I-L--F-----Y Q RDKV-P---K--------
	Trichinella sp. T6	KRX76053	--I-L--F-----Y Q RDKV-P---K--------
	Trichinella sp. T8	KRZ86089	--I-L--F-----Y Q RDKV-P---K--------
	Trichinella sp. T9	KRX58564	--I-L--F-----Y Q RDKV-P---K--------
	Trichinella spiralis	KRY35454	--I-L--F-----Y Q RDKV-P---K--------
	Trichinella murrelli	KRX38536	--I-L--F-----Y Q RDKV-P---K--------
	Trichinella britovi	KRY55180	--I-L--F-----Y Q RDKV-P---K--------
	Trichinella nativa	KRZ58235	--I-L--F-----Y Q RDKV-P---K--------
	Trichinella zimbabwensis	KRZ16106	--I-L--F-----Y Q RDKV-P---K--------
	Trichinella papuae	KRZ69043	--I-L--F-----Y Q RDKV-P---K--------
Outgroups (0/>100)	*Homo sapiens*	AAD54514	-QI-L--F-----Y S SE---P---K--------
	Mus musculus	BAC29077	-QI-L--F-----Y S SE---P---K--------

Supplementary Figure 5. Partial sequence alignments of a Cyclin dependent kinase 12 protein with a CSI consisting of a 1 aa deletion (shown in black box) that is specific for the *Caenorhabditis* genus and not present in any other nematode species.

			448		487
Caenorhabditis (6/6)	*Caenorhabditis elegans*	NP_001040875	SPFIDEKTRKKFMVSGGS	GG	DLKEELRKHIEEKFIPDFL
	Caenorhabditis briggsae	XP_002641795	------------------	--	----------D--------
	Caenorhabditis nigoni	PIC39092	------------------	--	----------D--------
	Caenorhabditis brenneri	EGT33558	------------------	--	-------------Y-----
	Caenorhabditis remanei	POM40570	------------------	--	----------D--------
	Caenorhabditis latens	OZG18173	------------------	--	----------D--------
Other Nematodes (0/42)	*Oesophagostomum dentatum*	KHJ77459	-----DN--N---IN--E		LV---IS-Y-D-QY-----
	Brugia timori	VDO35878	------N------INA-E		PVIS----Y---QY--E--
	Ancylostoma caninum	RCN37277	-----DN--N---IN--E		LV---IS-Y-DDQY-----
	Necator americanus	XP_013291223	-----DN--N---IN--E		TV---IS-Y-DDQY-----
	Strongylus vulgaris	VDM75523	-----DN--N---IN--E		LV---IS-Y-D-QY-----
	Brugia malayi	XP_001899466	------N------INA-E		PVIS----Y---QY--E--
	Wuchereria bancrofti	EJW86413	------N------INA-E		PVIS----Y---QY--E--
	Loa loa	XP_020306009	------N------INA-E		PVIS----Y---QY--E--
	Brugia pahangi	VDN83279	------N------INA-E		PVIS----Y---QY--E--
	Onchocerca ochengi	VDK61168	------N------INA-E		SVIS----Y--QQY--E--
	Ancylostoma ceylanicum	EYC32960	-----DN--N---IN--E		LV---IS-Y-DDQY-----
	Heligmosomoides polygyrus	VDP04674	------N--N---INS-E		VV-D-IS-YVD-QY-----
	Nippostrongylus brasiliensis	VDL74672	------N--N---INS-E		VV-D-IS-YVD-QY-----
	Litomosoides sigmodontis	VDK72923	------N------INA-E		PVIS--K-Y---QY--E--
	Acanthocheilonema viteae	VBB31712	------N------INA-E		PVIC----Y---QY--E--
	Teladorsagia circumcincta	PIO77325	------N--N---INS-E		IV-D-IS-Y-DDQY-----
	Onchocerca flexuosa	OZC09224	------N------INA-E		PVIS----Y--QQY--E--
	Haemonchus placei	VDO25093	-----DN--N---INS-E		LV-D-IS-Y-D-QY-----
	Haemonchus contortus	CDJ85549	-----DN--N---INS-E		LV-D-IS-Y-D-QY-----
	Dracunculus medinensis	VDN57909	----N-N-------NASE		S-PA--A-Y-D--YV----
	Thelazia callipaeda	VDN04664	------N------INS-Q		PVVN--K-Y-S-E---A--
	Enterobius vermicularis	VDD87376	------N------INA-E		SVIS--K-YMD-QY--E--
	Anisakis simplex	VDK47223	------N------INSSE		SVFN--S-Y-D-QYL----
	Cylicostephanus goldi	VDK62164	-----DN--S--KIN--D		FV-G-IS-Y-D-QY-----
	Angiostrongylus costaricensis	VDM52935	------N--N---INS-E		IS-G-IS-Y-DDQY-----
	Dictyocaulus viviparus	KJH42177	------T--N---INS-E		LV-G-IS-YVDDQY-----
	Strongyloides ratti	XP_024498558	----N-N-------NSNE		HIIDD-K-Y-D-NYL-T--
	Toxocara canis	KHN86630	------N------INS-E		AVLV--S-Y-DQQYL-E--
	Trichuris suis	KFD57671	----N---AN---IY---		-YANC-CQYM-Q-W-----
	Trichinella sp. T9	KRX61898	----N-R-A----IYS-N		-YVDC-KHYMD-EW-----
	Trichinella nativa	KRZ51817	----N-R-A----IYS-N		-YVDC-KHYMD-EW-----
	Trichinella sp. T6	KRX77867	----N-R-A----IYS-N		-YVDC-KHYMD-EW-----
	Trichinella spiralis	KRY33248	----N-R-A----IYS-N		-YVDC-KHYMD-EW-----
	Trichinella zimbabwensis	KRZ14091	----N-R-A----IYS-N		-YVDC-KHYMD-EW-----
	Trichinella papuae	KRZ78546	----N-R-A----IYS-N		-YVDC-KHYMD-EW-----
	Trichinella britovi	KRY45589	----N-R-A----IYS-N		-YVDC-KHYMD-EW-----
	Trichinella patagoniensis	KRY15637	----N-R-A----IYS-N		-YVDC-KHYMD-EW-----
	Trichinella sp. T8	KRZ85865	----N-R-A----IYS-N		-YVDC-KHYMD-EW-----
	Trichinella murrelli	KRX35808	----N-R-A----IYS-N		-YVDC-KHYMD-EW-----
	Trichinella nelsoni	KRX21447	----N-R-A----IYS-N		-YVDC-KHYMD-EW-----
	Trichinella pseudospiralis	KRY73116	----N-R-A----IYS-N		-YVDC-KHYMD-EW--E–
	Trichuris trichiura	CDW51999	----N---AN---IY---		-YANC-CQYM-Q-W-----
Outgroups (0/>100))	*Homo sapiens*	BAG56746	-----DN--R--LIYA-N		-YQGPGGLLDY-DKEI---
	Mus musculus	EDL34613	-----DN--R--LIYA-N		-YQGPGGLLDY-DKEI---

Supplementary Figure 6. Partial sequence alignments of an CRAL-TRIO domain-containing Sec14 protein with a CSI consisting of a 2 aa insertion (shown in black box) that is specific for the *Caenorhabditis* genus and not present in any other nematode species.

Group	Species	Accession	80		109
Caenorhabditis (6/6)	*Caenorhabditis elegans*	NP_001254942	HKNIIQFYGICKAT	G	NDFFIVTEYAEKGSL
	Caenorhabditis remanei	XP_003113095	--------------	-	--Y------------
	Caenorhabditis latens	OZG12911	--------------	-	--Y------------
	Caenorhabditis brenneri	EGT52079	--------------	-	-----------R---
	Caenorhabditis briggsae	CAP34484	--------------	-	--Y--------R---
	Caenorhabditis nigoni	PIC38130	--------------	-	--Y--------R---
Other Nematodes (0/17)	*Diploscapter pachys*	PAV57043	-------H----TG		T--Y-I--F-PN---
	Ancylostoma duodenale	KIH62964	-R---H------SM		S------D---N---
	Ancylostoma caninum	RCN31590	-R---H------SM		S------D---N---
	Oesophagostomum dentatum	KHJ88987	-R---H---V--TM		S------D---N---
	Ancylostoma ceylanicum	EPB70831	-R---H------SL		S------D---N---
	Anisakis simplex	VDK48365	-R-------VS-TD		P-------F--G---
	Enterobius vermicularis	VDD89857	-R-----F-VSHTN		P-------F--N---
	Toxocara canis	KHN72927	-R-----F-VSQTN		P-------F-DG---
	Strongyloides ratti	XP_024505381	-R---K---VSL-K		P-YY----F-AN---
	Onchocerca ochengi	VDK65711	----LRYF-AEVHG		---WLI--FHDN---
	Onchocerca flexuosa	OZC05979	----LRYF-SEVHG		---WLI--FHDN---
	Gongylonema pulchrum	VDN19314	-E-V--LF-VVTES		EPVM-----M-N---
	Brugia pahangi	VDN94600	-P---K---VATLK		DPIM--M-F-SG---
	Loa loa	XP_003139070	-P---K---VATLK		DPIM--M-F-SG---
	Acanthocheilonema viteae	VBB30169	-P---K---VATLK		DPIM--M-F-SG---
	Brugia timori	VDO38867	----LRYL-AEVHG		---WLI--FHDN---
	Brugia malayi	CDQ03802	----LRYL-AEVHG		---WLI--FHDN---
Outgroups (0/>100)	*Homo sapiens*	NP_057737	-R-------VILEP		PNYG--- ---SL-
	Mus musculus	BAC32371	-R-------VILEP		PNYG--- ---SL-

Supplementary Figure 7. Partial sequence alignments of a Mammalian ZAK kinase homolog protein with a CSI consisting of a 1 aa insertion (shown in black box) that is specific for the *Caenorhabditis* genus and not present in any other nematode species.

Group	Species	Accession	448		495
Caenorhabditis (6/6)	*Caenorhabditis elegans*	NP_001022706	DHRGTTNSFQMQS	LQ	KTPLSVLYSTEGSVLE
	Caenorhabditis nigoni	PIC13845	-------------	--	-----I----------
	Caenorhabditis briggsae	XP_002642442	-------------	--	-----I----------
	Caenorhabditis brenneri	EGT56754	------------A	--	----------------
	Caenorhabditis remanei	POM39899	-------------	--	---------V------
	Caenorhabditis latens	OZF89246	-------------	--	---------V------
Other Nematodes (0/40)	*Diploscapter pachys*	PAV86338	----------L--		----AQ---S------
	Nippostrongylus brasiliensis	VDL76464	-------A--L--		R---AL---S------
	Dictyocaulus viviparus	KJH43544	-------A--L--		----AQ---S------
	Ancylostoma caninum	RCN25685	-------A--L--		----AQ---S------
	Haemonchus placei	VDO28866	-------A--L--		----AQ---S------
	Ancylostoma duodenale	KIH62265	-------A--L--		----AQ---S------
	Ancylostoma ceylanicum	EPB72032	-------A--L--		----AQ---S------
	Haemonchus contortus	CDJ82671	-------A--L--		----AQ---S------
	Anisakis simplex	VDK54203	-------A--L--		----AQ---S------
	Necator americanus	XP_013299661	-------A--L--		----AQ---S------
	Toxocara canis	KHN78341	-------A--L--		----AQ---S------
	Brugia malayi	CRZ26330	-------A--V--		R---AQ---S------
	Heligmosomoides polygyrus	VDP09895	-------A--L--		----AQ---S------
	Oesophagostomum dentatum	KHJ90379	-------T--L--		R---AQ---S------
	Brugia pahangi	VDN89372	-------A--V--		R---AQ---S------
	Loa loa	XP_020303296	-------A--V--		R---AQ---S------
	Wuchereria bancrofti	EJW83303	-------A--V--		R---AQ---S------
	Litomosoides sigmodontis	VDK68074	-------A--V--		R---AQ---S------
	Thelazia callipaeda	VDN06480	-------A--V--		R---AQ---S------
	Onchocerca ochengi	VDK64108	-------A--V--		R---AQ---S------
	Onchocerca flexuosa	OZC09660	-------A--V--		R---AQ---S------
	Strongyloides ratti	XP_024507051	-------A--L--		----AQ---S------
	Brugia timori	VDO21918	-------A--V--		R---AQ---S------
	Acanthocheilonema viteae	VBB26691	-------A--V--		R---AQ---S------
	Pristionchus pacificus	PDM84936	----------K-A		R---AQ---S------
	Trichinella spiralis	XP_003378646	----------V--		--A-AQ---S------
	Trichinella nativa	OUC49818	----------V--		--A-AQ---S------
	Trichinella papuae	KRZ78135	----------V--		--A-AQ---S------
	Trichinella murrelli	KRX50328	----------V--		--A-AQ---S------
	Trichinella britovi	KRY57231	----------V--		--A-AQ---S------
	Trichinella sp. T8	KRZ92979	----------V--		--A-AQ---S------
	Trichinella pseudospiralis	KRZ31736	----------V--		--A-AQ---S------
	Trichinella nelsoni	KRX28020	----------V--		--A-AQ---S------
	Trichuris trichiura	CDW57862	----------V--		--A-AQ---S------
	Trichinella patagoniensis	KRY19114	----------V--		--A-AQ---S------
	Trichinella sp. T6	KRX82861	----------V--		--A-AQ---S------
	Trichinella sp. T9	KRX64000	----------V--		--A-AQ---S------
	Trichinella zimbabwensis	KRZ13685	----------V--		--A-AQ---S------
	Trichuris suis	KFD50868	----------V--		--A-AQ---S------
Outgroups (0/>100)	*Homo sapiens*	4HTX_A	-----N----VA-		-SV-AA ---S----M
	Mus musculus	AAH06845	-----N----VA-		-SV-AA ---S----M

Supplementary Figure 8. Partial sequence alignments of a probable 3',5'-cyclic phosphodiesterase pde-2 protein with a CSI consisting of a 2 aa insertion (shown in black box) that is specific for the *Caenorhabditis* genus and not present in any other nematode species.

Group	Species	Accession	7	CSI	35
Caenorhabditis (7/7)	*Caenorhabditis elegans*	NP_001256335	EFAAFKAITFLNPDA		DISLESKHAINEER
	Caenorhabditis remanei	OZF78699	---------------		-V---A--E--T--
	Caenorhabditis latens	OZG09782	---------------		-V---A--E--T--
	Caenorhabditis nigoni	PIC26490	---------------		-V---A--E--L--
	Caenorhabditis briggsae	XP_002636614	---------------		-V---A--E--L--
	Caenorhabditis brenneri	EGT48686	---------------		-V--DA-A------
Other Nematodes (0/16)	*Diploscapter pachys*	PAV80213	--------F------	D	-VDTS--Q-LS-G-
	Necator americanus	XP_013293410	--------F------	D	---SA--NMLS-G-
	Ancylostoma ceylanicum	EPB78040	------T-F------	D	-V-AA--PMLS-G-
	Teladorsagia circumcincta	PIO66922	------T-F------	D	-V-AA--PKLS-G-
	Ancylostoma caninum	RCN37032	------T-F------	D	-V-AA--PMLS-G-
	Haemonchus placei	VDO26611	------T-F------	D	-V-AI--PKLS-G-
	Dictyocaulus viviparus	KJH43863	---T---AL-F----	L	-L-SQA-PD-Y---
	Angiostrongylus costaricensis	VDM58595	---T---AL-F----	L	-L-SQA-PDVY---
	Pristionchus pacificus	PDM77897	--S-----F------	D	-V-AVC-QILSDG-
	Heligmosomoides polygyrus	VDO79280	------T-F------	D	-VNAA--AKLS-G-
	Haemonchus contortus	CDJ92296	---T---AL-F----	L	-L-SQA-PDVY---
	Nippostrongylus brasiliensis	VDL82945	------T-F------	D	-VNAN--PKLS-G-
	Anisakis simplex	VDK50975	--S-----F------	D	-L-PIV-E--SDC-
	Strongylus vulgaris	VDM85120	------T-F------	D	-VTSA--QTLS-G-
	Toxocara canis	KHN81931	--------F------	D	-V-VSA-E--SDC-
	Ancylostoma duodenale	KIH69436	---T---AL-F----	L	-L-NQA-PDVY---

Supplementary Figure 9. Partial sequence alignments of a Nuclear Hormone Receptor protein with a CSI consisting of a 1 aa deletion (shown in black box) that is specific for the *Caenorhabditis* genus and not present in any other nematode species. Homologous sequences for this protein were not observed for many non-nematode organisms in the BLASTP search.

Group	Species	Accession	1353		1393
Caenorhabditis (6/6)	*Caenorhabditis elegans*	NP_001256383	RREADEVVGWMYEKAKL	VM	SGDDSALAPSAIPHRLLKHEAF
	Caenorhabditis nigoni	PIC26477	-------M---H-----	-A	----A-----------------
	Caenorhabditis briggsae	CAP39687	-------M---H-----	-A	----A-----------------
	Caenorhabditis remanei	XP_003114094	-------M---H-----	-A	----T--T--------------
	Caenorhabditis latens	OZG15081	-------M---H-----	-A	----T--T--------------
	Caenorhabditis brenneri	EGT60238	-------M---H-----	-A	----AT--A-------------
Other Nematodes (0/27)	*Ancylostoma ceylanicum*	EYB93290	K-D-E-MIS-IH--K--		AN---HRDLTS-ANK-------
	Ancylostoma duodenale	KIH45166	K-D-E-MIS-I---K--		AN---HRDLTS-ANK-------
	Brugia malayi	CDP98888	--NIQ-LSQ-IA--R-I		AN---YRDTAS-TMK----K--
	Dictyocaulus viviparus	KJH48207	K-D-E-MIA-IHD-K--		AN---HKDL-S-ANK-------
	Diploscapter pachys	PAV55545	K-DG--MI--IA--K-T		AC---HRDLTS-ANK--R----
	Haemonchus contortus	CDJ92175	K-D-E-MIT-IH--K--		AN---HRDLTS-ANK-------
	Loa loa	XP_020307521	--NIQ-LSQ-IA--E-I		AN---YRDAAS-TMK----K--
	Necator americanus	XP_013296416	K-D-E-MIS-IH--K--		AN---HRDLTS-ANK-------
	Oesophagostomum dentatum	KHJ79216	--D-E-MIA-IHD-K--		AN---HRDLTS-ANK-------
	Onchocerca flexuosa	OZC12060	--NIQ-LTQ-IA--KRI		AN-G-YRDAAS-TMK----K--
	Strongyloides ratti	CEF62413	--D-F-LNC--V--KGI		L-EEDLSD---LRRH-QR--K-
	Wuchereria bancrofti	EJW88400	--NIQ-LSQ-IA--R-I		AN---YRDTAS-TMK----K--
	Toxocara canis	KHN72701	--NIQ-LSQ-IA--K-I		AN--AHKDAASLAMK----S--
	Trichinella britovi	KRY52189	---VE-LMQ-IG--MRI		VSS--YHSGLNLQK-IK-----
	Trichinella murrelli	KRX45023	---VE-LMQ-IG--MRI		VSS--YHSGLNLQK-IK-----
	Trichinella nativa	KRZ55341	---VE-LMQ-IG--MRI		VSS--YHSGLNLQK-IK-----
	Trichinella nelsoni	KRX16494	---VE-LMQ-IG--MRI		VSS--YHSGLNLQK-IK-----
	Trichinella papuae	KRZ76031	---VE-LMQ-IG--MRI		VSS--YHSGLNLQR-IK-----
	Trichinella patagoniensis	KRY15104	---VE-LMQ-IG--MRI		VSS--YHSGLNLQK-IK-----
	Trichinella pseudospiralis	KRX93022	---VE-LMQ-IG--MRI		VSS--YHSGLNFQR-IK-----
	Trichinella sp. T9	KRX58782	---VE-LMQ-IG--MRI		VSS--YHSGLNLQK-IK-----
	Trichinella sp. T8	KRZ97225	---VE-LMQ-IG--MRI		VSS--YHSGLNLQK-IK-----
	Trichinella sp. T6	KRX80779	---VE-LMQ-IG--MRI		VSS--YHSGLNLQK-IK-----
	Trichinella spiralis	KRY35759	---VE-LMQ-IG--MRI		VSS--YHSGLNLQK-IK-----
	Trichinella zimbabwensis	KRZ10195	---VE-LMQ-IG--MRI		VSS--YHSGLNLQR-IK-----
	Trichuris suis	KFD52473	K-N---LLV-VG--MRI		AV---YHSGLNLQR--K-----
	Trichuris trichiura	CDW52048	K-N---LLV-VG--MRI		AV---YHSGLNLQR--K-----
Outgroups (0/>100)	*Homo sapiens*	EAW87810	F-DS--LKS-VN--M-T		AT-EAYKD--NLQGKVQ--Q--
	Mus musculus	XP_006497875	F-DS--LKS-VN--M-T		AT-EAYKD--NLQGKVQ--Q--

Supplementary Figure 10. Partial sequence alignments of a SMAII-like (spore membrane assembly protein 2-like) aprotein with a CSI consisting of a 2 aa insertion (shown in black box) that is specific for the *Caenorhabditis* genus and not present in any other nematode species.

Group	Species	Accession	65		103
Caenorhabditis homologs 1 (0/6)	*Caenorhabditis elegans*	NP_001254962	WYFSKHYKGQVPTLEHDEG	K	KHVIESAVIPEYLDDIYPE
	Caenorhabditis brenneri	EGT52105	------------A------	-	---------------LF--
	Caenorhabditis remanei	POM37290	------------A------	-	-------H-------LF--
	Caenorhabditis latens	OZG10203	------------A------	-	-------H-------LF--
	Caenorhabditis briggsae	XP_002642151	------------A--L---	-	-------H-------LF--
	Caenorhabditis nigoni	PIC37794	------------A--L---	-	-------H-------LF–
Caenorhabditis homologs 2 (0/6)	*Caenorhabditis elegans*	NP_498728	--WT---Q-KA-AV--N -		-V----GF-------AF--
	Caenorhabditis brenneri	EGT56590	-------Q-KA-AV--- -		-V----GF-------AF--
	Caenorhabditis remanei	OZF78070	-------Q-KA-AI--N -		-I----GY-------AF--
	Caenorhabditis latens	OZG17648	-------Q-KA-AI--N -		-I----GY-------AF--
	Caenorhabditis nigoni	PIC40033	-FY----Q-KA-AI--- -		-I----GF-------AF--
	Caenorhabditis briggsae	XP_002642703	-FY----Q-KA-AI--- -		-I----GF-------AF--
Other Nematodes (0/22)	*Diploscapter pachys*	1240346389	---K---Q-K---F--- -		-V----LM-------AF-N
	Pristionchus pacificus	1253266495	---N--PQ-K---F-K- -		QI----GI------G----
	Anisakis simplex	1528886049	---K--PQ-NL------ -		-II---T--VQ---ELF--
	Toxocara canis	1535084310	---K--PE-K--A--YN -		NI-V---I--Q----LL--
	Loa loa	312091393	-FL---PE-T--V---- -		-L-SD-R--I-----AF--
	Strongyloides ratti	1376147712	--L--SPT----LF--- -		-YIT--N--L------FSA
	Brugia timori	1535290525	-FL---PE-T--L---- -		-L-PD-R--I-----AF--
	Brugia malayi	170591346	-FL---PE-T--L---- -		-L-PD-R--I-----AF--
	Wuchereria bancrofti	1528677019	-FL---PE-T--L---- -		-L-PD-R--I-----AF--
	Onchocerca ochengi	1528803377	-FY--NPE-V--V---N -		-L-NG-QM-I-----V---
	Ancylostoma ceylanicum	597840312	FL----PE-K--V---K -		QNI-D--L-S----W-Q-H
	Litomosoides sigmodontis	1528641557	-FLK-SPL-R--A--MN -		VTIY--S--A----EVF--
	Acanthocheilonema viteae	1511235889	-FLN-SPL-R--A--MN -		VTIY--S--A----E-F--
	Onchocerca flexuosa	1233063254	-FLN-SPL-R--AF-MN -		IIIY--S--A----E-F--
	Teladorsagia circumcincta	1276828720	--LA-SPL-S--A--IN -		-VIW--K-LA----EVF-T
	Haemonchus placei	1535225151	--LA-SPL-S--A--IN -		-VIW--K-LA----EVF-M
	Heligmosomoides polygyrus	1530586556	--LA-SPL-S--A--IN -		-VIW--K-LA----EVF-S
	Ancylostoma duodenale	748384251	--LA-SPL-S--A--IN -		RVIW--K-LA----EVF-S
	Haemonchus contortus	560138214	--LA-SPL-S--A--IN -		-VIW--K-LA----EVF-M
	Angiostrongylus costaricensis	1529400037	--LA-SPL-S--A--IN -		NVIF--K-LA----EVF-S
	Ancylostoma caninum	1432393302	--LA-SPL-S--A--IN -		RVIW--K-LA----EVF-S
	Necator americanus	915240016	--LA-SPL-S--A--IN -		NVIW--K-LA----EMF-S
Outgroups (0/>100)	*Homo sapiens*	NP_001177932	-F-K-NPF-L--V--NSQ-		QLIY---ITC----EA--G
	Mus musculus	NP_080895	--YT--PF--I-V--NSQC		QL-Y--VIAC-----V--G

Supplementary Figure 11. Partial sequence alignments of a Glutathione transferase omega-1 protein with a CSI consisting of a 1 aa insertion (shown in black box) that is specific for the *Caenorhabditis* genus and not present in any other nematode species. There are two homologs of this protein in the *Caenorhabditis* species, one of which contains the CSI.

Group	Species	Accession	46		90
Caenorhabditis (5/6)	*Caenorhabditis elegans*	NP_001254973	LCKIMLNETEQLAGLLAAKEIVAYQKSP	R	IIAIRSMADAFRKRSL
	Caenorhabditis briggsae	XP_002642446	-----------IPAM-ST--VLP-NT--	-	-V---A----------
	Caenorhabditis nigoni	PIC13841	-----------IPAM-ST--VLP-NTN-	-	-V---A----------
	Caenorhabditis remanei	POM39937	------------PS---T--FLP-HSNL	-	-----A----------
	Caenorhabditis brenneri	EGT56767	--------VD--PAIM-T-AV-PHLTDL	K	-V--L-----------
	Caenorhabditis latens	OZG01391	--V--DVPDEVNS--S-KLALKYSGSDL		E-MKAI-A-AQ----
Other Nematodes (0/31)	*Diploscapter pachys*	PAV73536	----LTDGAAAVPVM-ST--AA--SGPS		-S-VKQL-K--EE---
	Oesophagostomum dentatum	KHJ81246	-------DADSIPL--ST-VLQPVRGRE		-D-MKC--E--KE---
	Ancylostoma caninum	RCN32955	--------A-SIPL--ST-VLQPVRGRE		VD-MKC--E--KE---
	Ancylostoma duodenale	KIH66516	--------A-SIPL--ST-VLQPVRGRE		VD-MKC--E--KE---
	Ancylostoma ceylanicum	EPB72600	--------A-SIPL--ST-VLQPVRGRE		VD-MK---E--KE---
	Strongylus vulgaris	VDM73537	----ADSIPL---T-VLQPV-GRE		VD-MKC--E--KE---
	Dictyocaulus viviparus	KJH43501	-----M--A-SVPF--ST-VAQSIRGRD		VD-MKC--E--KE---
	Necator americanus	XP_013303392	--------ADSIPL--SN-VLQ-VHGRD		VD-MK---E--KE---
	Haemonchus contortus	CDJ89185	--------ADTIPL--ST-T-QSIRGRE		-E-MK---E--TE---
	Haemonchus placei	VDO28354	--------ADTIPL--ST-T-QSIRGRE		-E-MK---E--TE---
	Toxocara canis	KHN88339	-A------A-EVPLI-SG-IA-K-KGRD		VE-M--V-A--QQ---
	Anisakis simplex	VDK27391	-A---M--A-EVPSI-SG-LALK-KGRD		VE-MQ-V-Q--QQ---
	Soboliphyme baturini	VDO92414	-------LPDDVQS--S--IALK-VGRE		VD-MKAV---SK----
	Wuchereria bancrofti	EJW84880	-A------EREISL--SS-LAAK-SGRD		LD-MKDI-S--EN-C-
	Brugia pahangi	VDN91962	-A------EREIGL--SS-LAAK-SGRD		LD-MKDI-S--EN-C-
	Brugia malayi	CDP99050	-A------EREIGL--SS-LAAK-SGRD		LD-MKDI-S--EN-C-
	Loa loa	XP_020301826	-A------ETEVGS--SS-LATK-SGRD		LD-M-DI-T--EN---
	Acanthocheilonema viteae	VBB27213	-T------EIEVQS--SS-QAMK-SGRD		LD--K-I-A--DN---
	Onchocerca flexuosa	VDP14909	-A------ELEVHSI-SS-LAAK-SGRD		LD---TI-E-LEN---
	Onchocerca ochengi	VDK79902	VA------EVEVRSI-SS-LAAK-SGRD		LD---TI-E-LEN---
	Litomosoides sigmodontis	VDK81777	IA------ETEVPT--SG-QTIKHSSRD		LD-MKRI-E--HN---
	Strongyloides ratti	XP_024507529	---V--DSPDDVKS--SQ-LALK-SGRD		LD-M-AV-N-AK----
	Trichinella pseudospiralis	KRZ32121	-------LPDEVHI-VQG-LGMKHAGRQ		LE-MKAV-T-SKE---
	Trichinella zimbabwensis	KRZ06531	-------LPDEVHI-VQG-LGMKHAGRQ		LE-MKAV-T-SKE---
	Trichinella spiralis	XP_003375869	-------LPDDVHI-VQG-LGMKHAGRQ		LE-MKAV-T-SKE---
	Trichinella sp. T6	KRX70901	-------LPDDVHI-VQG-LGMKHAGRQ		LE-MKAV-T-SKE---
	Trichinella britovi	KRY56382	-------LPDDVHI-VQG-LGMKHAGRQ		LE-MKAV-T-SKE---
	Trichinella murrelli	KRX35477	-------LPDDVHI-VQG-LGMKHAGRQ		LE-MKAV-T-SKE---
	Trichinella nelsoni	KRX19233	-------LPDDVHI-VQG-LGMKHAGRQ		LE-MKAV-T-SKE---
	Trichuris trichiura	CDW54525	-------RP-DVSII-SG-LATK-VGRE		VD-MKAV-H-SKE---
	Trichuris suis	KFD62841	-------RP-DVSII-SG-LATK-VGRE		VD-MKAV-H-SKE---
Outgroups (0/>100)	*Homo sapiens*	5VGZ_X	------TP-DVQA-VSG -LALR-AGRQ		TE-LKCV-Q-SKN---
	Mus musculus	EDL15644	------TP-DVQA-VSG -LALR-AGRQ		TE-LKCV-Q-SKN---

Supplementary Figure 12. Partial sequence alignments of a 26S proteasome regulatory protein subunit with a CSI consisting of a 1 aa insertion (shown in black box) that is specific for the *Caenorhabditis* genus and not present in any other nematode species.

			92		130
Caenorhabditis (6/6)	*Caenorhabditis elegans*	NP_001256283	VKRAALNELVDHVSGA	P	KGSLSDAVYPEAIGMFSTNLFR
	Caenorhabditis remanei	XP_003100389	----------------	A	-------------H--------
	Caenorhabditis latens	OZG13626	----------------	A	-------------H--------
	Caenorhabditis brenneri	EGT55351	----------------	A	R----E-------N--------
	Caenorhabditis nigoni	PIC26850	-----------Y--A-	S	--T----------N--------
	Caenorhabditis briggsae	XP_002636421	-----------Y--A-	S	--T----------N--------
Other Nematodes (0/39)	*Diploscapter pachys*	PAV63148	I--------I--ITTT		--AIVE-I---V-K-V-K-I--
	Heligmosomoides polygyrus	VDO29824	---------I-Y--TC		RDAIT-NI-----N--AL----
	Dictyocaulus viviparus	KJH45358	---------I-Y--TC		RDAIT-NI-----N--AV----
	Enterobius vermicularis	VDD95190	I--------I--ITTV		--AITE-I---V-N-V-M-I--
	Gongylonema pulchrum	VDN17983	I--------I--ITTV		--AITESI---V-K-V-K-I--
	Onchocerca ochengi	VDK71745	I--------I--ITTV		--AITESI---V-K-V-K-I--
	Cylicostephanus goldi	VDN34744	I--------I--ITTT		--AIVESI---V-K-V-K-I--
	Teladorsagia circumcincta	PIO72193	---------I-Y--TC		RDAITEN------N--AL----
	Capitella teleta	ELU16316	--------M-EY-TQN		R-VIT------S-R--GV----
	Haemonchus contortus	CDJ84163	---------I-Y--TC		RDAITENI-----N--AV----
	Onchocerca flexuosa	VDO26608	I--------I--ITTV		--AITESI---V-K-V-K-I--
	Brugia timori	VDO24925	I--------I--ITTV		--AITELI---V-K-V-K-I--
	Brugia pahangi	VDN92265	I--------I--ITTV		--AITELI---V-K-V-K-I--
	Brugia malayi	CRZ24423	I--------I--ITTV		--AITELI---V-K-V-K-I--
	Loa loa	XP_020303422	I--------I--ITTV		--AITELI---V-K-V-K-I--
	Angiostrongylus costaricensis	VDM53307	----T----I-Y--TC		RDVIT-N------N--AV----
	Ancylostoma ceylanicum	EPB69273	I--------I--ITTT		--AIVESI---V-K-V-K-I--
	Necator americanus	XP_013294654	I--------I--ITTT		--AIVESI---V-K-V-K-I--
	Wuchereria bancrofti	VDM09038	I--------I--ITTV		--AITELI---V-K-V-K-I--
	Ancylostoma duodenale	KIH63659	I--------I--ITTT		--AIVESI---V-K-V-K-I--
	Thelazia callipaeda	VDN06789	I--------I--ITTV		--AITESI---I-K-A-K-I--
	Toxocara canis	KHN78251	I--------I--ITTV		--AITEPI---V-K-V-K-I--
	Ancylostoma caninum	RCN34933	---------I-Y--TC		RDVITEN------N--AL----
	Acanthocheilonema viteae	VBB32494	I--------I--ITTV		--AITELI---M-K-V-K-I--
	Acanthaster planci	XP_022087591	-----------F-THQ		R-VITEPI----VK---I-M--
	Haemonchus placei	VDO23997	---------I-Y--TC		RDAITENI-----N--AI----
	Oesophagostomum dentatum	KHJ92877	I--------I--ITTT		--AIVESI---V-K-V-K-I--
	Trichinella patagoniensis	KRY08455	---ST----I-Y--AN		REIFTESI---VVN--AV-T--
	Trichinella pseudospiralis	KRZ40843	---ST----I-Y--AN		REIFTESI---VVN--AV-T--
	Trichinella sp. T6	KRX84522	---ST----I-Y--AN		REIFTESI---VVN--AV-T--
	Trichinella zimbabwensis	KRZ12447	---ST----I-Y--AN		REIFTESI---VVN--AV-T--
	Trichinella sp. T9	KRX66464	---ST----I-Y--AN		REIFTESI---VVN--AV-T--
	Trichinella papuae	KRZ78327	---ST----I-Y--AN		REIFTESI---VVN--AV-T--
	Trichinella britovi	KRY60742	---ST----I-Y--AN		REIFTESI---VVN--AV-T--
	Trichinella sp. T8	KRZ86685	---ST----I-Y--AN		REIFTESI---VVN--AV-T--
	Trichinella nelsoni	KRX17598	---ST----I-Y--AN		REIFTESI---VVN--AV-T--
	Trichinella spiralis	KRY43433	---ST----I-Y--AN		REIFTESI---VVN--AV-T--
	Trichinella nativa	KRZ61030	---ST----I-Y--AN		REIFTESI---VVN--AV-T--
	Trichinella murrelli	KRX50117	---ST----I-Y--AN		REIFTESI---VVN--AV-T--
	Trichuris trichiura	CDW53420	---IT-T-MI-FIGNN		--I-TESI---VVK-V-V-I--
	Soboliphyme baturini	VDP13876	---LN----I-YI-SN		R-I-TERI---IFSV-AL-T--
Outgroups (0/>100)	*Homo sapiens*	NP_851308	----G---M-EYITHS		RDVVTE-I----VT---V----
	Mus musculus	NP_033384	----G---M-EYITHS		RDVVTE-I----VT---V----

Supplementary Figure 13. Partial sequence alignments of a Serine/Threonine protein phosphatase 2A Regulatory protein subunit with a CSI consisting of a 1 aa insertion (shown in black box) that is specific for the *Caenorhabditis* genus and not present in any other nematode species.

Group	Species	Accession	176		211
Caenorhabditis homolog 1 (0/6)	*Caenorhabditis elegans*	NP_001293265	LEALASLLASNKYFFD	V	NEPSWLDCKAFAVLAQFKY
	Caenorhabditis nigoni	PIC50339	--------N-------	-	--------------V----
	Caenorhabditis briggsae	CAR98690	--------N-------	-	--------------V----
	Caenorhabditis remanei	XP_003109234	--------N-------	-	--------------V----
	Caenorhabditis angaria	ACI49217	----SAQ-S------N	-	K------------------
	Caenorhabditis brenneri	EGT46436	--------N-------	-	--------------V----
Caenorhabditis homolog 2 (0/6)	*Caenorhabditis elegans*	NP_496604	-D-ISTQ-GDKP-L-G		SSIKTI-VT---H--ELI-
	Caenorhabditis latens	OZG15802	-D--STQ-GDK--L-G		-SIKTI-AT---H---LI-
	Caenorhabditis remanei	OZF85650	-D--STQ-GDK--L-G		-SIKAI-AT---H---LI-
	Caenorhabditis briggsae	XP_002631730	-D--SVQ-GEK--L-G		-TIKTI-VTT--H--ELI-
	Caenorhabditis nigoni	PIC47567	-D--SVQ-GEK--L-G		-TIKTI-VTT--H--ELI-
	Caenorhabditis brenneri	EGT44430	-D--SIQ--DKP-L-G		--VKSI-AV---H--ELI-
Other Nematodes (0/33)	*Diploscapter pachys*	PAV73992	-K-VSVI-GDKD---G		DI-TTI-AT---H-A-LV-
	Strongyloides ratti	XP_024505706	-K--SV--GDKP---G		DSFTTT-ATV-GQ-A--Y-
	Nippostrongylus brasiliensis	VDL79647	-D-INMF-GNK----G		DK-TT---TM-GH-A--L-
	Loa loa	XP_003137781	-T--SVF-GDKQ---G		ST-TT--AI--GN-A-LF-
	Toxocara canis	KHN80093	-L--SVF-ADKPF--G		SK-TT--AT--GH-T-LYF
	Pristionchus pacificus	PDM69804	-KSICIF-GDKP--GG		DR--TI-ATM-GH-ASLV-
	Thelazia callipaeda	VDN06273	-T--SVF-DDK-F--G		ST-TT--AT--GH-A-IY-
	Heligmosomoides polygyrus	VDO72907	-D-INMF-GNK----G		DK-VT---TM--H-S-YL-
	Strongylus vulgaris	VDM73748	FD--KTI-GEQ-FL-G		DSI-AT--TV-GH-ATTL-
	Ancylostoma caninum	RCN37793	-R--S-Q-G-KP-L-G		-Q-TEA-ALL-GH-FTIIT
	Ancylostoma ceylanicum	EPB70131	-R--S-Q-G-KP-L-G		-Q-TEA-ALL-GH-FTIIT
	Ancylostoma duodenale	KIH46080	-R--S-Q-G-KP-L-G		-Q-TEA-ALL-GH-FTIIT
	Dictyocaulus viviparus	KJH41673	-R--S-Q-G-KP-L-G		SQ-TEA-ALL-GH-FTIIT
	Angiostrongylus costaricensis	VDM51872	-R--S-Q-G-KP-L-G		-Q-TEA-ALL-GH-FTIIT
	Oesophagostomum dentatum	KHJ95729	-R--SLQ-G-KP-L-G		-Q-TEA-ALL-GH-FTIIT
	Haemonchus contortus	CDJ97722	FDTYRD--GDK-FL-G		D-I-SA--AV-SH-ATVL-
	Teladorsagia circumcincta	PIO68216	-R--S-Q-G-KPFL-G		-Q-TEA-ALL-GH-FTIIT
	Brugia pahangi	VDN85522	IA-IDVI-GDK-FL-G		VK-TTS-FTV-GH-ATSY-
	Wuchereria bancrofti	VDM20389	IA-IDVI-GDK-FL-G		VK-TTS-FTV-GH-ATSY-
	Brugia malayi	CTP81381	IA-IDVI-GDK-FL-G		VK-TTS-FTV-GH-ATSY-
	Dracunculus medinensis	VDN55503	IR-VDLI-GDK-FL-G		DK-TTA-FTV--H-ATTYF
	Brugia timori	VDO23595	IA-IDVI-GDK-FL-G		VK-TTS-FTV-GH-ATSY-
	Necator americanus	XP_013298689	-R--S-Q-G-KS-L-G		-Q-TEA-ALL-GH-FTIIT
	Acanthocheilonema viteae	VBB25728	IA-IDVI-GDK-FL-G		VK-TAS-FTV-GH-ATSY-
	Soboliphyme baturini	VDP46272	-RSFSVF-GEKA-LMG		DK-TKV-AAS-GH-S-LW-
	Onchocerca flexuosa	VDP13294	IA-IDII-GDK-FL-G		VK-TMS-FTV-GH-ATSY-
	Onchocerca ochengi	VDK63043	IA-IDII-GDK-FL-G		VK-TMS-FTV-GH-ATSY-
	Litomosoides sigmodontis	VDK75340	IAVIDVI-GDK-FL-G		MK-TAC-FTV-GH-ATSY-
	Trichinella sp. T9	KRX54821	VQY-SER-GEQP---G		-N--AF-AYV-GY-APIIL
	Trichinella murrelli	KRX37203	VQY-SER-GEQP---G		-N--AF-AYV-GY-APIIL
	Trichinella zimbabwensis	KRZ10266	VK-ISVF-GDKT-LSG		EK-TK--AV--GH-S-LW-
	Trichinella pseudospiralis	KRY82320	VK-ISVF-GDKT-LSG		EK-TK--AV--GH-G-LW-
	Trichinella spiralis	XP_003373651	VK-ISVF-GDKT-LSG		EK-TK--AV--GH-G-LW-

Supplementary Figure 14. Partial sequence alignments of a Failed axon connections-like protein with a CSI consisting of a 1 aa insertion (shown in black box) that is specific for the *Caenorhabditis* genus and not present in any other nematode species. There are two homologs of this protein in the *Caenorhabditis* species, one of which contains the CSI. The sequence alignment for homolog 1 of the species *Caenorhabditis latens* was not observed in the BLASTP search. Homologous sequences for this protein were not observed for many non-nematode organisms in the BLASTP search.

Group	Species	Accession	62	(boxed insert)	97
Caenorhabditis (5/5)	*Caenorhabditis briggsae*	XP_002632399	PDLEWQAWLRGTR	RNVDVMYWNLYFD	RFPPSDTEIA
	Caenorhabditis remanei	XP_003103438	-------------	-------------	----------
	Caenorhabditis latens	OZF87462	-------------	-------------	----------
	Caenorhabditis brenneri	EGT41957	-A-----------	-------------	------R--T
	Caenorhabditis elegans	NP_001255924	-------------	-------------	------R---
Other Nematodes (0/25)	*Ancylostoma duodenale*	KIH45006	-GV---S--K---		------D---
	Ancylostoma ceylanicum	EYC31492	-GV---S--K---		------D---
	Ancylostoma caninum	RCN50008	-GV---S--K---		------D---
	Cylicostephanus goldi	VDN23744	-GV---S--K---		------Q-L-
	Angiostrongylus costaricensis	VDM56116	-TV---S--K---		-----EE---
	Heligmosomoides polygyrus	VDP16140	-SV---S--K---		-----EQ---
	Dictyocaulus viviparus	KJH44480	VSV------K---		------E---
	Oesophagostomum dentatum	KHJ80075	-GI---S--K-V-		------Q-L-
	Necator americanus	XP_013290957	-SV---S--K-A-		-----EE--P
	Toxocara canis	VDM47853	-V-------K---		------E--Q
	Haemonchus placei	VDO38169	-S----S--K---		-----EE-LQ
	Diploscapter pachys	PAV87825	-PI--SS-VK---		-I--T-Q---
	Dracunculus medinensis	VDN53121	-SP--ES------		-----SE-LE
	Enterobius vermicularis	VDD97068	-SV--L---K---		KH---GE-AR
	Anisakis simplex	VDK55739	-S----S--K---		TS---LEQLS
	Gongylonema pulchrum	VDN21460	-PP--MT----V-		KL--TED-V-
	Wuchereria bancrofti	EJW87638	-SP--LT----I-		KL--TEE--L
	Brugia pahangi	VDN85631	-SP--LT----I-		KL--TEE--L
	Brugia malayi	XP_001895178	-SP--LT----I-		KL--TEE--L
	Litomosoides sigmodontis	VDK73513	-SP--LT----I-		KL--TQE--L
	Onchocerca ochengi	VDK71796	-SP--LT----M-		KL--TSE--L
	Loa loa	XP_003141261	-SP--LT----L-		KL--TQE--L
	Onchocerca flexuosa	VDO37915	-PP--LT----I-		KL--TPE--L
	Thelazia callipaeda	VDN02353	-PP--LT----I-		NI--TPE--M
	Acanthocheilonema viteae	VBB26498	-PP--LT----I-		KS--TPE-VL

Supplementary Figure 15. Partial sequence alignments of a NADH dehydrogenase 1 alpha sub-complex assembly factor 2 protein with a CSI consisting of a 13 aa insertion (shown in black box) that is specific for the *Caenorhabditis* genus and not present in any other nematode species. The sequence alignment of the species *Caenorhabditis nigoni* was not observed in the BLASTP search. Many homologous sequences for this protein were not observed for non-nematode organisms in BLASTP search.

Group	Species	Accession	Sequence (135–170)
Caenorhabditis (6/6)	*Caenorhabditis brenneri*	EGT45899	MKESSRIKFKTNDEGNGN [ ] FTAQLELTNYKAKDSGTY
	Caenorhabditis briggsae	XP_002645170	------------------ [ ] ------------------
	Caenorhabditis elegans	NP_001024421	------------------ [ ] ------------------
	Caenorhabditis briggsae	XP_002645169	I--T-------------- [ ] ----------TVE-----
	Caenorhabditis nigoni	PIC19380	---------------S-- [ ] --V-----K-TV------
Other Nematodes (0/41)	*Diploscapter pachys*	PAV75577	I--------T-SSD-GS- T Y--T---K----------
	Gongylonema pulchrum	VDK68579	---------GLTPD-GA- T -------K----------
	Onchocerca flexuosa	VDP13540	---------GLTPD-GA- T -------K----------
	Baylisascaris schroederi	ACL31520	---------SLTPD-GA- T -------K----------
	Ascaris suum	BAC06575	---------SLTPD-GA- T -------K----------
	Toxocara canis	KHN88700	---------SLTPD-GA- T -------K----------
	Anisakis simplex	VDK45727	---------NLTPD-GA- T -------K----------
	Litomosoides sigmodontis	VDM92971	---------GLTPD-GA- T -------K----------
	Brugia malayi	XP_001899521	---------GLTPD-GA- T -------K----------
	Loa loa	XP_003137600	---------GLTPD-GA- T -------K----------
	Brugia pahangi	VDN91454	---------GLTPD-GA- T -------K----------
	Brugia timori	VDO27064	---------GLTPD-GA- T -------K----------
	Onchocerca flexuosa	OZC08359	---------GLTPD-GA- T -------K----------
	Pristionchus pacificus	PDM66066	----T----NLSPD-GA- T -------K----------
	Thelazia callipaeda	VDN01526	---------GLVPD-GA- T -------K----------
	Necator americanus	XP_013297867	----T--R-L-TPD-PN- T ---K---K----------
	Ancylostoma caninum	RCN38460	----T--R-L-TPD-PN- T ---K---K----------
	Teladorsagia circumcincta	PIO67639	----T--R-L-SPD-PN- T ---K---K----------
	Vicugna pacos	XP_006219910	----T--R-L-SPD-PN- T ---K---K----------
	Haemonchus contortus	ADZ24723	----T--R-L-SPD-PN- T ---K---K----------
	Nippostrongylus brasiliensis	VDL70373	----T--R-V-TPD-PN- T ---K---K----------
	Ancylostoma duodenale	KIH68744	----T--R-L-TPD-PN- T ---K---K----------
	Dictyocaulus viviparus	KJH47142	----T--R-F-SPD-PN- T ---K---K----------
	Strongylus vulgaris	VDM74829	----T--R-L-TPD-PN- T ---K---K----------
	Dracunculus medinensis	VDN56371	---------GLTPD-GA- T ---M---K----------
	Ancylostoma ceylanicum	EPB67396	----T--R-L-TPD-PN- T ---K---K----------
	Oesophagostomum dentatum	KHJ98154	----T--R-F-TPD-PN- T ---K---K----------
	Angiostrongylus costaricensis	VDM63399	----T--R-F-TPD-PN- T ---K---K----------
	Enterobius vermicularis	VDD87484	---------GLSPD-GA- T Y--L---K------T---
	Haemonchus placei	VDO32598	------THII-TPDDRE- T ---K---KKC-PN---V-
	Strongyloides ratti	XP_024509872	I--GN--GIQNIKDSGNG N Y--L---R----------
	Heligmosomoides polygyrus	VDP57378	---T--Q--STPD-PN- T --VK---K----N-C---
	Soboliphyme baturini	VDP10778	---GK----VFEPDEGE- T -Q---Q-K--QK------
	Trichinella spiralis	XP_003369356	---GN--S-VLQQ-EEPM H Y------R---T----K-
	Trichinella nativa	KRZ50882	---GN--S-VLQQ-EEPM H Y------R---T----K-
	Trichinella pseudospiralis	KRX93535	---GN--S-VLQQ-EEPM H Y------R---T----K-
	Trichinella sp. T6	KRX74678	---GN--S-VLQQ-EEPM H Y------R---T----K-
	Trichinella nelsoni	KRX15059	---GN--S-VLQQ-EEPM H Y------R---T----K-
	Trichinella zimbabwensis	KRZ08200	---GN--S-VLQK-EEPM H Y------R---T----K-
	Trichuris suis	KFD56086	----N----ILQED-ENH A Y----H-K--------V-
	Trichuris trichiura	CDW58363	----N----ILQED-ENH A Y----H-K--------V-
Outgroups (0/>100)	*Homo sapiens*	CAD12458	IE--E-L-QSQ- -- I QF -TIC-VQLV-Q-L-
	Mus musculus	NP_085039	LGN---TRVR -D- T -DV-ITTLR----F

Supplementary Figure 16. Partial sequence alignments of a Disorganized muscle protein 1 protein with a CSI consisting of a 1 aa deletion (shown in black box) that is specific for the *Caenorhabditis* genus and not present in any other nematode species.

Group	Species	Accession	54		78
Caenorhabditis (6/6)	*Caenorhabditis elegans*	NP_001024482	VAKMWGNRKHNTK	D	MDYYKLSRAIR
	Caenorhabditis brenneri	EGT29973	-------------	-	-----------
	Caenorhabditis briggsae	XP_002644498	-------------	-	-----------
	Caenorhabditis nigoni	PIC18007	-------------	-	-----------
	Caenorhabditis latens	OZG25708	-------------	-	-----------
	Caenorhabditis remanei	OZG08321	-------------	-	-----------
Other Nematodes (0/47)	*Diploscapter pachys*	PAV75468	L-RL--EK-N---		-T-E-----M-
	Ancylostoma caninum	RCN52589	L-RL--E--N---		-T-E-----M-
	Ancylostoma ceylanicum	EYC16575	L-RL--E--N---		-T-E-----M-
	Ancylostoma duodenale	KIH66619	L-RL--E--N---		-T-E-----M-
	Nippostrongylus brasiliensis	VDL69293	L-RL--E--N---		-T-E-----M-
	Necator americanus	XP_013299955	L-RL--E--N---		-T-E-----M-
	Dictyocaulus viviparus	KJH43386	L-RL--E--N---		-T-E-----M-
	Strongylus vulgaris	VDM74654	L-RL--E--N---		-T-E-----M-
	Heligmosomoides polygyrus	VDP00764	L-RL--E--N---		-T-E-----M-
	Angiostrongylus costaricensis	VDM54068	L-RL--E--N---		-T-E-----M-
	Teladorsagia circumcincta	PIO69733	L-RL--E--N---		-T-E-----M-
	Haemonchus contortus	CDJ88503	L-RL--E--N---		-T-E-----M-
	Haemonchus placei	VDO23124	L-RL--E--N---		-T-E-----M-
	Pristionchus pacificus	PDM84773	L-RL--E--N-Q-		-T-E-----M-
	Toxocara canis	KHN82222	L-RL--EK-N-Q-		-T-E-----M-
	Anisakis simplex	VDK42097	L-RL--EK-N-Q-		-T-E-----M-
	Strongyloides ratti	XP_024498651	L-RL--EK-N-Q-		-T-E-----M-
	Gongylonema pulchrum	VDN40596	L-RL--EK-N-Q-		-T-E-----M-
	Litomosoides sigmodontis	VDK75710	L-QL--E--N-Q-		-T-E-----M-
	Onchocerca flexuosa	OZC10835	L-QL--E--N-Q-		-T-E-----M-
	Acanthocheilonema viteae	VBB28739	L-QL--E--N-Q-		-T-E-----M-
	Brugia pahangi	VDN81825	L-QL--E--N-Q-		-T-E-----M-
	Brugia malayi	XP_001896909	L-QL--E--N-Q-		-T-E-----M-
	Onchocerca ochengi	VDK65325	L-QL--E--N-Q-		-T-E-----M-
	Wuchereria bancrofti	EJW86133	L-QL--E--N-Q-		-T-E-----M-
	Loa loa	XP_020307442	L-QL--E--N-Q-		-T-E-----M-
	Brugia timori	VDO09094	M-QL--E--N-Q-		-T-E-----M-
	Enterobius vermicularis	VDD84864	L-RL--KK-N-A-		-T-E-----M-
	Dracunculus medinensis	VDN57494	L--L--EK-N-Q-		-T-E-----M-
	Thelazia callipaeda	VDN01542	L-RF--Q--NR-Q		-N-D----SL-
	Soboliphyme baturini	VDO94383	--RR--E--NKPN		-N-D-----L-
	Oesophagostomum dentatum	KHJ94529	L-RF--Q--NRAQ		-N-D----SL-
	Cylicostephanus goldi	VDK85783	L-RF--Q--NRAQ		-N-D----SL-
	Trichinella nelsoni	KRX19706	L-RL--MK-N-SR		-T-E-----M-
	Trichinella britovi	KRY49119	L-RL--MK-N-SR		-T-E-----M-
	Trichinella zimbabwensis	KRZ07064	L-RL--MK-N-SR		-T-E-----M-
	Trichinella pseudospiralis	KRX90070	L-RL--MK-N-SR		-T-E-----M-
	Trichinella spiralis	XP_003382250	L-RL--MK-N-SR		-T-E-----M-
	Trichinella sp. T8	KRZ90731	L-RL--MK-N-SR		-T-E-----M-
	Trichinella patagoniensis	KRY20366	L-RL--MK-N-SR		-T-E-----M-
	Trichinella murrelli	KRX36026	L-RL--MK-N-SR		-T-E-----M-
	Trichinella papuae	KRZ75693	L-RL--MK-N-SR		-T-E-----M-
	Trichinella nativa	KRZ56336	L-RL--MK-N-SR		-T-E-----M-
	Trichuris suis	KFD57331	M-QL--LK-N-SR		-T-E-----M-
	Trichuris trichiura	CDW53786	M-QL--LK-N-SR		-T-E-----M-
	Trichinella sp. T6	KRX73529	--RL--L--NKPN		-N-D-----L-
	Trichinella sp. T9	KRX62147	--RL--L--NKPN		-N-D-----L-
Outgroups (0/>100)	*Homo sapiens*	AAA52411	--RR--K--NKP-		-N-E----GL-
	Mus musculus	CBA13447	---L--L--NK-N		-N-D-----L-

Supplementary Figure 17. Partial sequence alignments of an ETS (E26 transformation-specific) class transcription factor protein with a CSI consisting of a 1 aa insertion (shown in black box) that is specific for the *Caenorhabditis* genus and not present in any other nematode species.

Group	Species	Accession	430		466
Caenorhabditis (6/6)	*Caenorhabditis brenneri*	EGT38541	HLIGADSLPSRDQLDNNL	V	PFLPHLSNAEERAFVILN
	Caenorhabditis briggsae	CAP24891	------------------	-	------------------
	Caenorhabditis elegans	NP_001293390	------------------	-	------------------
	Caenorhabditis nigoni	PIC55883	------------------	-	------------------
	Caenorhabditis remanei	XP_003114575	------------------	-	------------------
	Caenorhabditis latens	OZG11498	------------------	-	------------------
Other Nematodes (0/36)	*Heligmosomoides polygyrus*	VD067565	------------------		Q--------D------V-
	Nippostrongylus brasiliensis	VDL79693	------------------		Q--------D------V-
	Haemonchus contortus	CDJ80361	------------------		Q--------D------V-
	Oesophagostomum dentatum	KHJ83169	---------------S--		Q--------D------V-
	Ancylostoma ceylanicum	EYC04497	---------------S--		Q--------D------V-
	Necator americanus	XP_013308737	---------------S--		Q--------D------V-
	Angiostrongylus costaricensis	VDM59654	------------------		Q----I---D------V-
	Ancylostoma caninum	RCN53200	---------------S--		Q--------D------V-
	Ancylostoma duodenale	KIH54863	---------------S--		Q--------D------V-
	Dictyocaulus viviparus	KJH53574	------------------		Q----I-SPD------V-
	Anisakis simplex	VDK50202	---------T-----P--		Q------T-D------M-
	Toxocara canis	KHN85013	--V------------P--		Q------TPD--------
	Thelazia callipaeda	VDN04120	--V---T---K----T--		Q------APD-----VM-
	Dracunculus medinensis	VDN54005	----S-----KN---P--		Q------SPD-----V--
	Brugia timori	VD022248	------T---K----T--		Q------APD---IAVM-
	Onchocerca ochengi	VDK66508	------T---K----A--		Q------APD---I-VM-
	Wuchereria bancrofti	VDM20530	------T---K----T--		Q------APD---IAVM-
	Brugia pahangi	VDN94771	------T---K----T--		Q------APD---IAVM-
	Brugia malayi	XP_001901269	------T---K----T--		Q------APD---IAVM-
	Litomosoides sigmodontis	VDK75146	------T---K----A--		Q------APD---IAVM-
	Onchocerca flexuosa	OZC12839	------T---K----A--		Q------APD---IAVM-
	Loa loa	XP_003140865	----------K----TD-		Q------APD---IAVM-
	Strongyloides ratti	XP_024507890	----S----CKG---I--		Q------AS---I-LVS-
	Trichinella zimbabwensis	KRZ03728	--V-P--M-ATA-VES-A		SYI-NI-S-K---M-VI-
	Trichinella papuae	KRZ71993	--V-P--M-AIS-VES-T		SYI-NI-S-K---M-VI-
	Soboliphyme baturini	VDP22009	--L-PG-I-TKS--EEH-		Q-F----SPD---M-VS-
	Trichinella pseudospiralis	KRZ34022	--V-P--M-AIA-VES-A		SYI-NI-S-K---M-VI-
	Trichinella patagoniensis	KRY17046	--V-P--M-AIA-VES-A		SYI-NI-S-K---M-VI-
	Trichinella spiralis	KRY28779	--V-P--M-AIA-VES-A		SYI-NI-S-K---M-VI-
	Trichinella nativa	OUC40531	--V-P--M-AIA-VES-A		SYI-NI-S-K---M-VI-
	Trichinella sp. T9	KRX57766	--V-P--M-AIA-VES-A		SYI-NI-S-K---M-VI-
	Trichinella sp. T6	KRX72098	--V-P--M-AIA-VES-A		SYI-NI-S-K---M-VI-
	Trichinella britovi	KRY49319	--V-P--M-AIA-VES-A		SYI-NI-S-K---M-VI-
	Trichinella murrelli	KRX38890	--V-P--M-AIA-VES-A		SYI-NI-S-K---M-VI-
	Trichinella sp. T8	KRZ87771	--V-P--M-AIA-VES-A		SYI-NI-S-K---M-VI-
	Trichinella nelsoni	KRX14027	--V-P--M-AIA-IET-A		SYI-NI-S-K---M-VI-

Supplementary Figure 18. Partial sequence alignments of a Glycine-rich domain-containing protein with a CSI consisting of a 1 aa insertion (shown in black box) that is specific for the *Caenorhabditis* genus and not present in any other nematode species. Homologous sequences for this protein were not observed for many non-nematode organisms in the BLASTP search.

Group	Species	Accession	364	(box)	399
Caenorhabditis (6/6)	*Caenorhabditis elegans*	NP_001255199	DEAVAYGAAIMAAVLSG		AEEVQDMRLIDVIPMSIG
	Caenorhabditis remanei	XP_003097172	---------V-------		V----------M------
	Caenorhabditis brenneri	EGT52753	---------V-------		V----------M------
	Caenorhabditis briggsae	CAP27311	---------V-----T-		V----------M------
	Caenorhabditis nigoni	PIC49760	---------V-----T-		V----------M------
	Caenorhabditis latens	OZG13115	---------V-------		V----------M------
Other Nematodes (0/55)	*Diploscapter pachys*	PAV72947	---------VQ--I---	DK	S-A---LL-L--A-L-
	Anisakis simplex	VDK75896	---------VQ--I---	DK	S-T---LL-L--A-L-
	Meloidogyne enterolobii	AHG55039	---------VQ--I---	DK	S-N---LL-L--A-L-
	Ditylenchus destructor	AHF71091	---------VQ--I-C-	DK	S-N---LL-L--A-L-
	Haemonchus contortus	CDJ88360	---------VQ--I---	DK	S-A---LL-L--A-L-
	Onchocerca volvulus	P11503.2	---------VQ--I---	DK	S-A---LL-L--A-L-
	Brugia malayi	AAR01009	---------VHS-I---	DK	S-A---LL-L--A-L-
	Teladorsagia circumcincta	PIO68034	---------VQ--I---	DK	S-A---LL-L--A-L-
	Dirofilaria immitis	AAA28298	---------VQ--I---	DK	S-A---LL-L--A-L-
	Onchocerca flexuosa	VDP11183	---------VQ--I---	DK	S-A---LL-L--A-L-
	Litomosoides sigmodontis	VDK67489	---------VQ--I---	DK	S-T---LL-L--A-L-
	Brugia pahangi	AAA27857	---------VQ--I---	DK	S-A---LL-L--A-L-
	Bursaphelenchus mucronatus	ACU00685	---------VQ--I-C-	DK	S-N---LL-L--A-L-
	Enterobius vermicularis	VDD92026	---------VQ--I---	DK	S-T---LL-L--A-L-
	Toxocara canis	VDM39102	---------VQ--I---	DK	S-A---LL-L--A-L-
	Acanthocheilonema viteae	VBB31912	---------VQ--I---	DK	S-A---LL-L--A-L-
	Wuchereria bancrofti	AAF32254	---------VQ--I---	DK	S-A---LL-L--A-L-
	Pristionchus pacificus	PDM79784	---------VQ--I---	DK	S-A---LL-L--A-L-
	Onchocerca ochengi	VDK66984	---------VQ--I---	DK	S-A---LL-L--A-L-
	Gongylonema pulchrum	VDN19430	---------VQ--I---	DK	S-A---LL-L--A-L-
	Anisakis pegreffii	AIU38247	---------VQ--I---	DK	S-T---LL-L--A-L-
	Heterodera glycines	AAN78300	---------VQ--I---	DK	S-T---LL-L--A-L-
	Strongylus vulgaris	VDM66254	---------VQ--I---	DK	S-A---LL-L--A-L-
	Haemonchus placei	VDO34176	---------VQ--I---	DK	S-A---LL-L--A-L-
	Loa loa	XP_020306180	---------VQ--I---	DK	S-A---LL-L--A-L-
	Ancylostoma caninum	AKO62674	---------VQ--I---	DK	S-A---LL-L--A-L-
	Ancylostoma ceylanicum	EPB70622	---------VQ--I---	DK	S-A---LL-L--A-L-
	Thelazia callipaeda	VDN04920	--------SVQ--I---	DK	S-A---LL-L--A-L-
	Heligmosomoides polygyrus	VDO81416	---------VQ--I---	DK	S-A---LL-L--A-L-
	Necator americanus	XP_013293739	---------VQ--I---	DK	S-A---LL-L--A-L-
	Strongyloides ratti	XP_024502726	---------VQ--I---	DK	S-A---LL-L--A-L-
	Dracunculus medinensis	ADI54942	---------VQ--I---	DK	S-A---LL-L--A-L-
	Angiostrongylus vasorum	ABV46675	---------VQ--I---	DK	S-A---LL-L--A-L-
	Setaria digitata	AAD13154	---------VQ--I---	DK	S-A---LLFV--A-L-
	Dictyocaulus viviparus	KJH46194	---------VQ--I---	DK	S-A---LL-L--A-L-
	Parastrongyloides trichosuri	AAF87583	---------VQ--I---	DK	S-A---LL-L--A-L-
	Aphelenchoides besseyi	AKH40414	-----F---VQ--I-C-	DK	S-N---LL-L--A-L-
	Angiostrongylus costaricensis	VDM64089	---------VQ--I---	DK	S-A---LL-L--A-L-
	Nippostrongylus brasiliensis	VDL68456	---------VQ--I---	DK	S-A---LL-L--A-L-
	Soboliphyme baturini	VDP32341	---------VQ--I---	EK	H-A---LL-L--T-L-
	Bursaphelenchus xylophilus	ABG74349	---------VQ--I-C-	DK	S-K---LL-L--A-L-
	Trichinella zimbabwensis	KRZ18308	---------VQ--I---	EK	H-A---LL-L--T-L-
	Trichinella britovi	KRY58600	---------VQ--I---	EK	H-A---LL-L--T-L-
	Trichinella spiralis	KRY38488	---------VQ--I---	EK	H-A---LL-L--T-L-
	Trichinella papuae	KRZ69869	---------VQ--I---	EK	H-A---LL-L--T-L-
	Trichinella pseudospiralis	KRY78483	---------VQ--I---	EK	H-A---LL-L--T-L-
	Trichinella sp. T8	KRZ89860	---------VQ--I---	EK	H-A---LL-L--T-L-
	Trichinella nelsoni	KRX27085	---------VQ--I---	EK	H-A---LL-L--T-L-
	Trichinella sp. T9	KRX62956	---------VQ--I---	EK	H-A---LL-L--T-L-
	Trichinella sp. T6	KRX70487	---------VQ--I---	EK	H-A---LL-L--T-L-
	Trichinella murrelli	KRX42824	---------VQ--I---	EK	H-A---LL-L--T-L-
	Trichinella nativa	KRZ63003	---------VQ--I---	EK	H-A---LL-L--T-L-
	Trichinella patagoniensis	KRY05166	---------VQ--I---	EG	N-K---LL-L--T-L-
	Trichuris trichiura	CDW56034	---------VQ--I---	DK	H-A---LL-L--T-L-
	Trichuris suis	KFD56098	---------VQ--I---	DK	H-A---LL-L--T-L-
Outgroups (0/>100)	*Homo sapiens*	BAD96505	---------VQ--I---	DK	S-N---LL-L--T-L-
	Mus musculus	BAE42451	---------VQ--I---	DK	S-N---LL-L--T-L-

Supplementary Figure 19. Partial sequence alignments of a Heat shock protein 70 protein with a CSI consisting of a 2 aa deletion (shown in black box) that is specific for the *Caenorhabditis* genus and not present in any other nematode species. Homologous partial sequence alignments of other nematode species were not observed in the BLASTP search.

Group	Species	Accession	441		476
Caenorhabditis (6/6)	*Caenorhabditis elegans*	NP_001255199	GERVLCSHNRQLGEISL		PIQPKRRGESVVDITLEVD
	Caenorhabditis remanei	XP_003097172	-----------------		--N--K-------V-----
	Caenorhabditis nigoni	PIC49759	-----------------		--N--K-------------
	Caenorhabditis briggsae	XP_002632174	-----------------		--H--K-------------
	Caenorhabditis latens	OZG13117	----------------		-N--K-------V-----
	Caenorhabditis brenneri	EGT52753	---------------T-		--N--K----I--V-----
Other Nematodes (0/16)	*Pristionchus pacificus*	PDM75588	---A-TKD-H---NFE-	S	G-P-TP--VPQIEVVF-I-
	Anisakis simplex	VDK65257	---A-TKD-N---RFD-	T	Q-P-AP--VPQIEV-FDL-
	Soboliphyme baturini	VDO98716	---EMAA--KL--QF--	V	G-P-AP--VPQIEV-FDI-
	Trichinella murrelli	KRX50303	---EMA---KL--QF--	V	G-P-AP--VPQIEV-FDI-
	Trichinella sp. T6	KRX76699	---EMA---KL--QF--	V	G-P-AP--VPQIEV-FDI-
	Trichinella sp. T9	KRX62429	---EMA---KL--QF--	V	G-P-AP--VPQIEV-FDI-
	Trichinella pseudospiralis	KRZ33796	---EMA---KL--QF--	V	G-P-AP--VPQIEV-FDI-
	Trichinella spiralis	KRY33164	---EMA---KL--QF--	I	G-P-AP--VPQIEV-FDI-
	Trichinella nativa	KRZ57762	---EMA---KL--QF--	V	G-P-AP--VPQIEV-FDI-
	Trichinella britovi	KRY58424	---EMA---KL--QF--	V	G-P-AP--VPQIEV-FDI-
	Trichinella patagoniensis	KRY23083	---EMA---KL--QF--	V	G-P-AP--VPQIEV-FDI-
	Trichinella sp. T8	KRZ92825	---EMA---KL--QF--	V	G-P-AP--VPQIEV-FDI-
	Trichinella nelsoni	KRX21108	---EMA---KL--QF--	I	G-P-AP--VPQIEV-FDI-
	Trichuris suis	KFD55321	---DMA---KL--QF--	V	G-P-AP--VPQIEV-FDI-
	Trichuris trichiura	CDW60853	---DMA---KL--QF--	V	G-P-AP--VPQIEV-FDI-
	Trichinella zimbabwensis	KRZ16667	---EMA---KL--QF--	V	G-P-AP--VPQIEVAFDI-
Outgroups (0/>200)	*Homo sapiens*	AAH15699	---AMTKD-NL--KFE-	T	G-P-AP--VPQIEV-FDI-
	Mus musculus	BAE29904	---AMTKD-NL- -KFE-	T	G-P-AP--VPQIEV-FDI-

Supplementary Figure 20. Partial sequence alignments of a Heat shock protein 70 protein with a CSI consisting of a 1 aa deletion (shown in black box) that is specific for the *Caenorhabditis* genus and not present in any other nematode species. Homologous partial sequence alignments of other nematode species were not observed in the BLASTP search.

Group	Species	Accession	123		151
Caenorhabditis (6/6)	*Caenorhabditis elegans*	NP_001024661	GPIIGQFCGHFEN		RILNTSSHNALTLWWH
	Caenorhabditis brenneri	EGT30233	-------------		K---------------
	Caenorhabditis remanei	POM49287	-------------		----M-----------
	Caenorhabditis briggsae	XP_002644596	-------------		----M-----------
	Caenorhabditis nigoni	PIC18220	-------------		----M-----------
	Caenorhabditis latens	OZG25193	-------------		----M-----------
Other Nematodes (0/28)	*Diploscapter pachys*	PAV79326	---V-----RIAP	L	TV--V--E--MS----
	Soboliphyme baturini	VDP02479	------I--KVK-	R	TL-A--TY--M-----
	Gongylonema pulchrum	VDK29616	---L-----AIK-	S	SE-TV-TE--------
	Toxocara canis	VDM24306	---V-----IIR-	A	SE-AI--E--------
	Onchocerca flexuosa	OZC09977	---L-----TIR-	A	SE-AV-TE--------
	Onchocerca ochengi	VDK65250	---L-----TIR-	A	SE-AV-TE--------
	Thelazia callipaeda	VDN01074	---L-----TIR-	A	SE-AV-TE--------
	Wuchereria bancrofti	VDM14467	---L-----TIR-	A	SE-AV-TE--------
	Angiostrongylus costaricensis	VDM52576	---------KRT-	A	SE-S--TL--------
	Ancylostoma duodenale	KIH43381	---------KLT-	T	SE-SV-TL--------
	Ancylostoma ceylanicum	EPB66588	---------KLT-	T	SE-SV-TL--------
	Anisakis simplex	VDK46880	---V-----IIR-	A	SE-AI--E--------
	Ancylostoma caninum	RCN52527	---------KLT-	T	SE-SV-TL--------
	Cylicostephanus goldi	VDK45323	---------KRT-	A	SE-SS-TL--------
	Heligmosomoides polygyrus	VDP41706	---------RRT-	T	TE-SV-TM--------
	Litomosoides sigmodontis	VDK71987	---L-----TIR-	A	SE-AV-TE--------
	Brugia malayi	XP_001899277	---L-----TIR-	A	SE-AV-TE--------
	Brugia pahangi	VDN90800	---L-----TIR-	A	SE-AV-TE--------
	Brugia timori	VDO32583	---L-----TIR-	A	SE-AV-TE--------
	Loa loa	XP_020303780	---L-----TIR-	A	SE-AV-TE--------
	Acanthocheilonema viteae	VBB30517	---L-----TIR-	A	SE-AV-TE--------
	Oesophagostomum dentatum	KHJ99798	---------KHS-	A	SE-SV-TL--------
	Haemonchus placei	VDO80095	---------RRN-	V	TD-SL-TM--------
	Haemonchus contortus	CDJ98395	---------RRN-	V	TD-SL-TM--------
	Necator americanus	XP_013293602	---------KRT-	T	SE-SV-TL--------
	Strongylus vulgaris	VDM79066	---------KRT-	A	SE-SV-TL--------
	Dictyocaulus viviparus	KJH41256	---------KRS-	I	SE-SI-TL--------
	Dracunculus medinensis	VDN58727	---L-----LIR-	A	SE-AV-TE--------

Supplementary Figure 21. Partial sequence alignments of an Abnormal cell migration protein 13 protein with a CSI consisting of a 1 aa deletion (shown in black box) that is specific for the *Caenorhabditis* genus and not present in any other nematode species. Homologous sequences for this protein were not observed for many non-nematode organisms in the BLASTP search.

Group	Species	Accession	143	CSI	175
Caenorhabditis (6/6)	*Caenorhabditis remanei*	XP_003089575	CIHLAACSAGQWLPM	S	NPHLPADLFTSCLTTPI
	Caenorhabditis latens	OZG15523	---------------	-	-----------------
	Caenorhabditis elegans	NP_001293858	---------------	-	-----------------
	Caenorhabditis nigoni	PIC32995	-----------Y---	-	-----------------
	Caenorhabditis briggsae	XP_002632899	-----------Y---	-	-----------------
	Caenorhabditis brenneri	EGT53100	-----------F---	-	-----------------
Other Nematodes (0/38)	*Diploscapter pachys*	PAV89799	--Q-G--R-DEF--T		--D-------A---S--
	Soboliphyme baturini	VDP10977	-------M-D-V---		--E--------------
	Angiostrongylus costaricensis	VDM53003	--Q----REDER---		--E--------------
	Heligmosomoides polygyrus	VDO79049	--Q----REDER--T		--D--------------
	Nippostrongylus brasiliensis	VDL64489	--Q----REDER--T		--E--------------
	Haemonchus placei	VDO26483	--Q----REDER--T		--D--------------
	Dictyocaulus viviparus	KJH44437	--Q----REDER--T		--E--------------
	Haemonchus contortus	CDJ97476	--Q----REDER--T		--D--------------
	Strongylus vulgaris	VDM65179	-------REDER--T		-AE--------------
	Ancylostoma ceylanicum	EYC05101	-------REDER--T		-AD--------------
	Ancylostoma duodenale	KIH69120	-------REDER--T		-AD--------------
	Ancylostoma caninum	RCN45671	-------REDER--T		-AD--------------
	Pristionchus pacificus	PDM76953	--Q----KV-EK---		AAE-------A------
	Acanthocheilonema viteae	VBB31036	---I---GDHEL---		DSR---------IL---
	Onchocerca ochengi	VDK78612	---IG--ADHEL---		DSR---------IL---
	Thelazia callipaeda	VDN06650	---IG--GDNEL---		DSR---------IL---
	Necator americanus	XP_013298983	----G--REDER--T		-ADV-------------
	Litomosoides sigmodontis	VDK77318	---IG--GDQEL---		DSR---------IL---
	Wuchereria bancrofti	EJW88084	---IG--GDHEL---		DSR---------IL---
	Brugia malayi	CRZ22876	---IG--GDHEL---		DSR---------IL---
	Brugia pahangi	VDN91436	---IG--GDHEL---		DSR---------IL---
	Loa loa	XP_020306206	---IG--GDHEL---		DSR---------IL---
	Brugia timori	VDO52578	---IG--GDHEL---		DSR---------IL---
	Onchocerca flexuosa	VDO44874	---IG--ADHEL---		DSR---------IL---
	Trichinella murrelli	KRX38173	--Q----ARD-T--T		D-A-------C------
	Trichinella spiralis	KRY35465	--Q----ARD-T--T		D-A-------C------
	Trichinella sp. T8	KRZ85612	--Q----ARD-T--T		D-A-------C------
	Trichinella nativa	KRZ49841	--Q----ARD-T--T		D-A-------C------
	Trichinella britovi	KRY46767	--Q----ARD-T--T		D-A-------C------
	Trichinella sp. T6	KRX75381	--Q----ARD-T--T		D-A-------C------
	Trichinella nelsoni	KRX15293	--Q----ARD-T--T		D-A-------C------
	Trichinella pseudospiralis	KRY84144	--Q----ARD-T--T		D-A-------C------
	Trichinella zimbabwensis	KRZ05231	--Q----ARD-T--T		D-A-------C------
	Trichinella papuae	KRZ73461	--Q----ARD-T--T		D-A-------C------
	Trichinella patagoniensis	KRY09658	--Q----ARD-T--T		D-A-------C------
	Trichinella sp. T9	KRX61440	--Q----ARD-T--T		D-A-------C------
	Trichuris suis	KFD50574	--Q----A-D-E--T		H-D-------C------
	Trichuris trichiura	CDW56927	--Q----A-D-E--T		H-D-------C------
Outgroups (0/>200)	*Homo sapiens*	NP_001156506	--Q----E-TEL---		I-D--------------
	Mus musculus	XP_006534426	--Q----E-HEL---		I-D--------------

Supplementary Figure 22. Partial sequence alignments of a Regulatory-associated protein of mTOR-like protein with a CSI consisting of a 1 aa insertion (shown in black box) that is specific for the *Caenorhabditis* genus and not present in any other nematode species.

Group	Species	Accession	141		170
Caenorhabditis (6/6)	*Caenorhabditis elegans*	NP_001024661	SSHNALTLWWHSN		PNGSNSKGFKLHWGSFR
	Caenorhabditis remanei	XP_003117772	-------------		Q-------------A--
	Caenorhabditis briggsae	CAP33035.2	-------------		--------------A--
	Caenorhabditis latens	OZG25193	-------------		Q-------------A--
	Caenorhabditis nigoni	PIC18220	-------------		------R-------A--
	Caenorhabditis brenneri	EGT30233	-------------		-----------------
Other Nematodes (0/28)	*Diploscapter pachys*	PAV79335	--E--MS----A-	SEV	SD-EKR-------SA--
	Anisakis simplex	VDK46880	--E---------D	ADL	-PNHSGE--R-L-SA--
	Toxocara canis	VDM24306	--E---------D	ADL	-PNHSGE--R-L-SA--
	Angiostrongylus costaricensis	VDM52576	-TL--------TD	SLL	-QQHPA---R---NA--
	Dictyocaulus viviparus	KJH41256	-TL--------TD	SLL	-QQHPA---R---NA--
	Gongylonema pulchrum	VDK29616	-TE--------TD	AEL	-LQHSGE--R-L-SA--
	Litomosoides sigmodontis	VDK71987	-TE--------TD	AEL	-FHH-GE--R-L-SA--
	Onchocerca ochengi	VDK65250	-TE--------TD	AEL	-FHH-GE--R-L-SA--
	Loa loa	XP_020303780	-TE--------TD	AEL	-FHH-GE--R-L-SA--
	Brugia malayi	CRZ25839	-TE--------TD	AEL	-FHH-GE--R-L-SA--
	Brugia timori	VDO32583	-TE--------TD	AEL	-FHH-GE--R-L-SA--
	Acanthocheilonema viteae	VBB30517	-TE--------TD	AEL	-FHH-GE--R-L-SA--
	Brugia pahangi	VDN90800	-TE--------TD	AEL	-FHH-GE--R-L-SA--
	Nippostrongylus brasiliensis	VDL77150	-TM--------TD	ALL	-QQHPA---Q---NA--
	Wuchereria bancrofti	EJW83984	-TE--------TD	AEL	-FHH-GD--R-L-SA--
	Haemonchus placei	VDO80095	-TM--------TD	AIL	-QQHPA---Q---NA--
	Haemonchus contortus	CDJ85812	-TM--------TD	AIL	-QQHPA---Q---NA--
	Thelazia callipaeda	VDN01074	-TE--------TD	AQT	SFHRSGE--R-L-SA--
	Dracunculus medinensis	VDN58727	-TE---------D	ANL	-INH-GE--R-L-SA--
	Onchocerca flexuosa	OZC09977	-TE--------TD	ADL	-FHH-GE--R-L-SA--
	Heligmosomoides polygyrus	VDP41706	-TM--------TD	ALL	SQQHPA---H---NA--
	Ancylostoma duodenale	KIH43381	-TL--------TD	PLL	AQQHPA---R-Q-NA--
	Oesophagostomum dentatum	KHJ99798	-TL--------TD	PLL	AQQHPA---R-Q-NA--
	Ancylostoma ceylanicum	EYB97855	-TL--------TD	PLL	AQQHPA---R-Q-NA--
	Necator americanus	XP_013293602	-TL--------TD	PLL	AQQHPA---R-Q-NA--
	Strongylus vulgaris	VDM79066	-TL--------TD	PLL	AQQHPA---R-Q-NA--
	Ancylostoma caninum	RCN52527	-TL--------TD	PLL	AQQHPA---R-Q-NA--
	Cylicostephanus goldi	VDK45323	-TL--------TD	PLL	AQQHPA---R-Q-NA--

Supplementary Figure 23. Partial sequence alignments of an Abnormal cell migration protein 13 protein with a CSI consisting of a 3 aa deletion (shown in black box) that is specific for the *Caenorhabditis* genus and not present in any other nematode species. Homologous sequences for this protein were not observed for many non-nematode organisms in the BLASTP search.

			220		251	
Caenorhabditis (6/6)	*Caenorhabditis elegans*	NP_001024660	KGFKLHWGSFRVSKTGNC		VTGEFSCGNGECIP	
	Caenorhabditis remanei	XP_003117772	--------A---------		--------------	
	Caenorhabditis latens	OZG25193	--------A---------		--------------	
	Caenorhabditis briggsae	XP_002644596	--------A----R----		--------------	
	Caenorhabditis brenneri	EGT30233	-------------R----		--------------	
	Caenorhabditis nigoni	PIC18220	R-------A----R----		--------------	
Other Nematodes (0/22)	*Diploscapter pachys*	PAV79326	-------SA---*I-VP-	S	KNR----R-D-F--	*VKKAA
	Nippostrongylus brasiliensis	VDL77150	---Q---NA---TAQVP-	Y	YGR--A---NQ---	
	Dictyocaulus viviparus	KJH41256	---R---NA---TANVP-	S	S-R--A-SHN----	
	Angiostrongylus costaricensis	VDM52576	---R---NA---TANVP-	S	SSR--A--QS----	
	Oesophagostomum dentatum	KHJ99798	---R-Q-NA---TANVP-	S	TSR--A--GN----	
	Haemonchus placei	VDO80095	---Q---NA---TAGVP-	S	AGR--A--HDQ---	
	Haemonchus contortus	CDJ98395	---Q---NA---TAGVP-	S	AGR--A--HDQ---	
	Toxocara canis	VDM24306	E--R-L-SA--K-N-AP-	N	TQR--T-A-Q----	
	Cylicostephanus goldi	VDK45323	---R-Q-NA---TANVP-	S	PSR--A--GN----	
	Ancylostoma ceylanicum	EPB66588	---R-Q-NA--ITSNVP-	S	SSR--A--GN----	
	Ancylostoma caninum	RCN52527	---R-Q-NA--ITSNVP-	S	SSR--A--GN----	
	Ancylostoma duodenale	KIH43381	---R-Q-NA--ITSNVP-	S	SSR--A--GN----	
	Necator americanus	XP_013293602	---R-Q-NA---TTNVP-	S	PSR--A--GN----	
	Anisakis simplex	VDK46880	E--R-L-SA--K-NSEP-	N	TQR--T-A-H----	
	Rhincodon typus	XP_020369132	E---FRHLGDDKIPPRRQ	C	LPN-----S-----	
	Brugia pahangi	VDN90800	E--R-L-SA--K-NIAP-	N	IQR--T-K-Q---A	
	Brugia malayi	CRZ25839	E--R-L-SA--K-NIAP-	N	IQR--T-K-Q---A	
	Onchocerca ochengi	VDK65250	E--R-L-SA--K-NVAP-	N	AQR--T-K-Q---A	
	Onchocerca flexuosa	OZC09977	E--R-L-SA--K-NVAP-	N	AQR--T-K-Q---A	
	Gongylonema pulchrum	VDK29616	E--R-L-SA--K-NIAP-	N	NQR--T-K-Q----	
	Acanthocheilonema viteae	VBB30517	E--R-L-SA--K-NIAP-	N	AQR--T-K-Q---A	
	Loa loa	XP_020303780	E--R-L-SA--K-NIAP-	N	AQR--T-R-Q---A	
Outgroups (0/>200)	*Homo sapiens*	XP_011509485	-	L	HN -----------	
	Mus musculus	BAD90371	-	L	PNQ -R-TS-Q-VL	

Supplementary Figure 24. Partial sequence alignments of an Abnormal cell migration protein 13 protein with a CSI consisting of a 1 aa deletion (shown in black box) that is specific for the *Caenorhabditis* genus and not present in any other nematode species.

			1460		1497
Caenorhabditis (6/6)	*Caenorhabditis elegans*	NP_500018	MLAAWFTFLLHDHLKTY	D	AGKKLYELFWGIKQQMEKGP
	Caenorhabditis remanei	XP_003099038	-----------------	-	--------------------
	Caenorhabditis nigoni	PIC37034	----------------F	-	--------------------
	Caenorhabditis briggsae	XP_002634865	----------------F	-	--------------------
	Caenorhabditis brenneri	EGT33873	----------------F	-	--------------------
	Caenorhabditis latens	OZG19088	-----------------	-	--------------------
Other Nematodes (0/27)	*Diploscapter pachys*	PAV71746	--T------MY---LKE		P-RC--N-YYS----I----
	Ancylostoma ceylanicum	EPB68178	---------M--Y-RNF		---R--D-Y-----------
	Ancylostoma duodenale	KIH66538	---------M--Y-RNF		---R--D-Y-----------
	Brugia malayi	CRZ23938	-----Y-I-MYRY-TEC		--R-----Y-AM--------
	Dictyocaulus viviparus	KJH51742	------------Y-RNF		---R--D-Y-----------
	Haemonchus contortus	CDJ83976	---------M--Y-RN-		---R--D-Y-----------
	Loa loa	XP_020306889	-----Y-I-MYRY-TEC		--R-----Y-AM--------
	Oesophagostomum dentatum	KHJ95623	---------M--Y-RNF		---R--D-Y-----------
	Onchocerca flexuosa	OZC11101	-----Y-I-MYRY-TEC		--R-----Y-AM--------
	Pristionchus pacificus	PDM64129	------S--M-NY-EES		---R-FD-Y-----------
	Strongyloides ratti	CEF60012	--S---A-FMYKF-RDC		G-QQ-FQ-Y-S---TT----
	Wuchereria bancrofti	EJW80286	-----Y-I-MYRY-TEC		--R-----Y-AM--------
	Teladorsagia circumcincta	PIO53665	---------M--Y-RN-		---R--D-Y-----------
	Toxocara canis	KHN85487	-----Y---M-RY-RER		--Q-----Y-A----V----
	Trichinella britovi	KRY48614	---------M-KY-LDC		--QP-FT-Y-A----V----
	Trichinella murrelli	KRX43850	---------M-KY-LDC		--QP-FT-Y-A----V----
	Trichinella nativa	KRZ55370	---------M-KY-LDC		--QP-FT-Y-A----V----
	Trichinella nelsoni	KRX17302	---------M-KY-LDC		--QP-FT-Y-A----V----
	Trichinella patagoniensis	KRY11761	---------M-KY-LDC		--QP-FT-Y-A----V----
	Trichinella pseudospiralis	KRY68294	---------M-KY-LDC		--QP-FT-Y-A----V----
	Trichinella sp. T6	KRX84092	---------M-KY-LDC		--QP-FT-Y-A----V----
	Trichinella sp. T8	KRZ95482	---------M-KY-LDC		--QP-FT-Y-A----V----
	Trichinella sp. T9	KRX59942	---------M-KY-LDC		--QP-FT-Y-A----V----
	Trichinella spiralis	KRY35089	---------M-KY-LDC		--QP-FT-Y-A----V----
	Trichinella zimbabwensis	KRZ11671	---------M-KY-LDC		--QP-FT-Y-A----V----
	Trichuris suis	KFD50499	---------M-KY-LEC		--QP-FM-Y-A----V----
	Trichuris trichiura	CDW56793	---------MYKY-LEC		--QP-FM-Y-A----V----
Outgroups (0/>200)	*Homo sapiens*	XP_011529485	--TN-------KF--EC		--EP-FL -YCA----
	Mus musculus	BAA13190	--TN-------KF--EC		--EP-FL -YCA----

Supplementary Figure 25. Partial sequence alignments of a Plexin protein with a CSI consisting of a 1 aa insertion (shown in black box) that is specific for the *Caenorhabditis* genus and not present in any other nematode species.

Group	Species	Accession	1020	CSI	1070
Caenorhabditis homolog 1 (4/4)	*Caenorhabditis elegans*	NP_503362	GCFRPTHYYVLHDTWT	G	KPDSFWPTVTHALTYNFCRSTTTVALPAPVLYAH
	Caenorhabditis brenneri	EGT57012	-VA---------N--E	K	L-V----CI-Y--------T-S---Q---L----
	Caenorhabditis latens	OZF81803	-LA--------WN--P	-	CLPT-----------M---A-S------------
	Caenorhabditis remanei	OZF90277	-LA--------WN--P	-	CLPT-----------M---A-S------------
Caenorhabditis homolog 2 (5/5)	*Caenorhabditis brenneri*	EGT32090	-IA------------D		PYVA-----------L---T-SV-S--S------
	Caenorhabditis briggsae	CAP22335	-LA---------NN-K		V-GT-----------L---A----N---------
	Caenorhabditis latens	OZG10323	-LV--------WN--K		CLPT-----------T------------------
	Caenorhabditis nigoni	PIC13092	-LA---------ND-K		V-GT-----------L-----S--N--------R
	Caenorhabditis remanei	OZF89967	-LV--------WN--K		CLPT-----------T------------------
Other Nematodes (0/46)	*Diploscapter pachys*	PAV88210	-TS--A-----W-E-Q		PSPA----I----S--YV-CMKSISI-G-IF---
	Ascaris suum	AEF32755	-TS--A--T--Y-S-N		LSPDD-QQ-AY--CHLYA-CARS-SI----Y---
	Gongylonema pulchrum	VDN33859	-TS--S-----Y-S-N		LTPDE-QHL-F--CHVYA-CNRS-SI----Y---
	Onchocerca flexuosa	OZC04814	-TS--A--I--Y-S-H		LSADE-QQLVY--CHVYA-CNKS-SI----Y---
	Acanthocheilonema viteae	VBB30375	-TS--S-----Y-S-N		LTSDE-QQL-F--CHLYA-CNRS-SI----Y---
	Loa loa	XP_020304102	-TS--S--T--Y-S-H		LSADE-QQMIY--CHVYA-CNKS-SI----Y---
	Anisakis simplex	VDK54211	-TS--A--T--Y-S-N		LSADQ-QQMAY--CHLYT-CARS-SI---IY---
	Thelazia callipaeda	VDM97873	-TS--S--I--Y-S-L		LSADE-QQMIY--CHVYA-CNKS-SI----Y---
	Wuchereria bancrofti	VDM09830	-TS--S--T--Y-S-H		LSADE-QQMIY--CHVYA-CNKS-SI----Y---
	Litomosoides sigmodontis	VDK72737	-TS--S--I--Y-S-H		LSADD-QQMIY--CHVYA-CNKS-SI----Y---
	Brugia malayi	CRZ24698	-TS--S--T--Y-S-L		LSADE-QQMIY--CHIYA-CNKS-SI----Y---
	Brugia timori	VDO27404	-TS--S--T--Y-S-L		LSADE-QQMIY--CHIYA-CNKS-SI----Y---
	Toxocara canis	VDM38397	-TS--A--T--Y-S-N		LLADQ-QQMAY--CHLYA-CARS-SI----Y---
	Onchocerca ochengi	VDM92461	-TS--A--I--Y-S-H		LSADE-QQMVY--CHVYA-CNKS-SI----Y---
	Brugia pahangi	VDN87401	-TS--S--T--Y-S-L		LSADE-QQMIY--CHIYA-CNKS-SI----Y---
	Soboliphyme baturini	VDP12733	-TS--S--H--W-DNN		LTADELQQL-YQ-CHTYV-C-RS-SI---AY---
	Necator americanus	XP_013299213	-TS--S--H--W-DNE		LTADELQQL-YQMCHTYV-C-RS-SI---AY---
	Teladorsagia circumcincta	PIO64661	-TS--S--H--W-DNE		LTADELQQL-YQMCHTYV-C-RS-SI---AY---
	Heligmosomoides polygyrus	VDO99643	-TS--S--H--W-DNE		LTADELQQL-YQMCHTYV-C-RS-SI---AY---
	Haemonchus contortus	CDJ87551	-TS--S--H--W-DNQ		LTADELQQL-YQMCHTYV-C-RS-SI---AY---
	Trichinella papuae	KRZ78211	-TS--S--H--W-DNS		LTADELQQL-YQ-CHTYV-C-RS-SI---AY---
	Haemonchus placei	VDO55419	-TS--S--H--W-DNQ		LTADELQQL-YQMCHTYV-C-RS-SI---AY---
	Dictyocaulus viviparus	KJH45308	-TS--S--H--W-DNE		LTADELQQL-YQMCHTYV-C-RS-SI---AY---
	Oesophagostomum dentatum	KHJ99080	-TS--S--H--W-DNE		LTADELQQL-YQMCHTYV-C-RS-SI---AY---
	Ancylostoma caninum	RCN47663	-TS--S--H--W-DNE		LTADELQQL-YQMCHTYV-C-RS-SI---AY---
	Pristionchus pacificus	PDM64682	-TS--S--H--W-DSS		LTADELQQL-YQMCHTYV-C-RS-SI---AY---
	Nippostrongylus brasiliensis	VDL77997	-TT--A--T-VY-E-S		QSPDKIYEMCYR-CFLYA-CRRP-S--C--Y---
	Strongyloides ratti	XP_024508426	-TS--S--H--W-DNQ		I-ADELQQL-YQMCHTYV-C-RS-SI---AY---
	Ancylostoma ceylanicum	EPB70479	-TS--S--H--W-DNE		LTADELQQL-YQMCHTYV-C-RS-SI---AY---
	Dracunculus medinensis	VDN60348	-TS--S--H--W-DNN		LTADELQQL-YQMCHTYV-C-RS-SI---AY---
	Angiostrongylus costaricensis	VDM60322	-TS--S--H--W-DNE		LTADELQQL-YQMCHTYV-C-RS-SI---AY---
	Enterobius vermicularis	VDD85869	-TS--S--H--W-DNS		LTADELQQL-YQMCHTYV-C-RS-SI---AY---
	Ancylostoma duodenale	KIH64115	-TS--AR-H--W-DNN		FSADEMQAI-YGMCHTYG-CARS-SI----Y--
	Trichinella zimbabwensis	KRZ12513	-TS--S--H--W-DNS		LTADELQQL-YQ-CHTYV-C-RS-SI---AY---
	Trichinella sp. T9	KRX66470	-TS--S--H--W-DNS		LTADELQQL-YQ-CHTYV-C-RS-SI---AY---
	Trichinella pseudospiralis	KRY88551	-TS--S--H--W-DNS		LTADELQQL-YQ-CHTYV-C-RS-SI---AY---
	Trichinella britovi	KRY60812	-TS--S--H--W-DNS		LTADELQQL-YQ-CHTYV-C-RS-SI---AY---
	Trichinella murrelli	KRX48413	-TS--S--H--W-DNS		LTADELQQL-YQ-CHTYV-C-RS-SI---AY---
	Trichinella nelsoni	KRX23112	-TS--S--H--W-DNS		LTADELQQL-YQ-CHTYV-C-RS-SI---AY---
	Trichinella spiralis	XP_003374460	-TS--S--H--W-DNS		LTADELQQL-YQ-CHTYV-C-RS-SI---AY---
	Trichinella sp. T6	KRX84491	-TS--S--H--W-DNS		LTADELQQL-YQ-CHTYV-C-RS-SI---AY---
	Trichinella nativa	KRZ52696	-TS--SY-H--W-DNN		FSADEMQQL-YQ-CHTYV-C-RS-SI---AY---
	Trichinella patagoniensis	KRY16554	-TS--SY-H--W-DNN		FSADEMQQL-YQ-CHTYV-C-RS-SI---AY---
	Trichinella sp. T8	KRZ95990	-TS--SY-H--W-DNN		FSADEMQQL-YQ-CHTYV-C-RS-SI---AY---
	Trichuris suis	KHJ40100	-TS--S--H--W-DNN		LSADELQQL-YQ-CHTYV-C-RS-SI---AY---
	Trichuris trichiura	CDW54952	-TS--S--H--W-DNN		LSADELQQL-YQ-CHTYV-C-RS-SI---AY---
Outgroups (0/>200)	*Homo sapiens*	EAW92208	-TS--S--H--W-DNR		FSSDELQIL-YQ-CHTYV-C-RS-SI---AY---
	Mus musculus	NP_067286	-SVS----N-IY-SSG		LKPDHIQRL-YK-CHVYYNWPGVIRV---CQ---

Supplementary Figure 26. Partial sequence alignments of a Piwi-like protein protein with a CSI consisting of a 1 aa insertion (shown in black box) that is specific for the *Caenorhabditis* genus and not present in any other nematode species. There are two homologs of this protein in the *Caenorhabditis* species, one of which contains the CSI. The sequence alignment for homolog 1 of the species *Caenorhabditis nigoni* and *Caenorhabditis briggsae* was not observed in the BLAST search.

Group	Species	Accession	70		99
Caenorhabditis homolog 1 (6/6)	*Caenorhabditis elegans*	NP_001123124	RAVVFRLGRLVP		DVKGPGIFFIIPCIDT
	Caenorhabditis latens	OZG25387	----------I-		E--------------Q
	Caenorhabditis remanei	XP_003117911	----------I-		E--------------Q
	Caenorhabditis briggsae	XP_002645067	----------I-		---------------Q
	Caenorhabditis nigoni	PIC19183	----------I-		E---------L-----
	Caenorhabditis brenneri	EGT33172	----------L-		E--------------Q
Caenorhabditis homolog 2 (6/6)	*Caenorhabditis brenneri*	EGT29906	---I------M-	G	GA--------V-----
	Caenorhabditis briggsae	XP_002644793	---I------M-	G	GA--------V-----
	Caenorhabditis nigoni	PIC18440	---I------M-	G	GA--------V-----
	Caenorhabditis elegans	NP_001024566	---I------M-	G	GA--------V-----
	Caenorhabditis latens	OZG24410	---I------M-	G	GA--------V-----
	Caenorhabditis remanei	OZF84041	---I------IK	G	GT----L--VL-----
Other Nematodes (0/26)	*Diploscapter pachys*	PAV57820	-V-I--I----F	G	GAR---MI--------
	Ancylostoma ceylanicum	EPB70273	-V-I--I----F	G	GAR---MI--------
	Ancylostoma duodenale	KIH68818	---I------M-	G	GA-------VL----S
	Ascaris suum	ERG80781	-V-I--I----F	G	GAR---MI-V------
	Brugia malayi	XP_001895378	---I------MT	G	KAR---L---L----S
	Dictyocaulus viviparus	KJH43651	-V-I--I----F	G	GAR---MI--------
	Haemonchus contortus	CDJ89985	---I------M-	G	GA--------V-----
	Loa loa	XP_020302578	---I------MT	G	RAR---L---L----S
	Necator americanus	XP_013298666	-V-I--I----F	G	GAR---MI--------
	Oesophagostomum dentatum	KHJ99155	-V-I--I----F	G	GAR---MI-V------
	Pristionchus pacificus	PDM83777	---I------M-	G	GA--------V-----
	Strongyloides ratti	CEF62830	---I------M-	G	GA--------V-----
	Toxocara canis	KHN87924	---I------M-	G	GA--------V-----
	Trichinella britovi	KRY60162	-V-I------M-	G	VAR---LV--M-----
	Trichinella murrelli	KRX44897	-V-I------M-	G	VAR---LV--M-----
	Trichinella nativa	KRZ62504	-V-I------M-	G	VAR---LV--M-----
	Trichinella nelsoni	KRX16776	-V-I------M-	G	VAR---LV--M-----
	Trichinella papuae	KRZ74240	-V-I------M-	G	VAR---LV--M-----
	Trichinella patagoniensis	KRY18828	-V-I------M-	G	VAR---LV--M-----
	Trichinella pseudospiralis	KRZ26557	-V-I------M-	G	VAR---LV--M-----
	Trichinella sp. T6	KRX75325	-V-I------M-	G	VAR---LV--M-----
	Trichinella sp. T8	KRZ95329	-V-I------M-	G	VAR---LV--M-----
	Trichinella sp. T9	KRX56998	-V-I------M-	G	VAR---LV--M-----
	Trichinella spiralis	XP_003380470	-V-I------M-	G	VAR---LV--M-----
	Trichuris suis	KHJ43320	-V-I------M-	G	VAR---LV--M-----
	Trichuris trichiura	CDW55292	-V-I------M-	G	VA----LV--M-----
Outgroups (0/>100)	*Homo sapiens*	NP_001257456	E--II-----IL	Q	GGA----L---L--T-
	Mus musculus	AAH58224	E---------IQ	A	DKA----LILVL----

Supplementary Figure 27. Partial sequence alignments of a Stomatin protein with a CSI consisting of a 1 aa deletion (shown in black box) that is specific for the *Caenorhabditis* genus and not present in any other nematode species. There are two homologs of this protein in the *Caenorhabditis* species, one of which contains the CSI.

Group	Species	Accession	257		280
Caenorhabditis (6/6)	*Caenorhabditis elegans*	NP_001123140	QCQGCVWSKR		RTAGERVYTVKATI
	Caenorhabditis nigoni	PIC17654	----------		--------------
	Caenorhabditis briggsae	XP_002644340	----------		--------------
	Caenorhabditis latens	OZG25237	----------		--------------
	Caenorhabditis remanei	POM50721	----------		--------------
	Caenorhabditis brenneri	EGT30457	----------		--------------
Other Nematodes (0/27)	*Diploscapter pachys*	PAV65431	-----N-A--	H	KLHSDK-C------
	Cylicostephanus goldi	VDK64261	----------	H	-LPN-K--------
	Ancylostoma duodenale	KIH53513	----------	H	-LPN-K--------
	Necator americanus	XP_013303822	----------	H	-LPN-K--------
	Ancylostoma ceylanicum	EPB79691	----------	H	-LPN-K--------
	Strongylus vulgaris	VDM75426	----------	H	-LPN-K--------
	Haemonchus placei	VDO32683	----------	H	-LSNDK--------
	Ancylostoma caninum	RCN33227	----------	H	-LPN-K--------
	Oesophagostomum dentatum	KHJ98476	----------	H	-LPN-K--------
	Anisakis simplex	VDK42437	----------	R	-KGP-A--S-R---
	Angiostrongylus costaricensis	VDM58682	----------	H	-LGNDK--------
	Haemonchus contortus	CDJ90030	----------	H	-LSNDK--------
	Dictyocaulus viviparus	KJH48789	----------	H	-LSNDK--------
	Toxocara canis	VDM36538	--------R-	R	KKGP-A--S-R---
	Dracunculus medinensis	VDN50960	-----I----	H	KKGP-A--S-----
	Brugia timori	VDO47024	-------N--	N	KQGP-T--------
	Brugia malayi	XP_001898198	-------N--	N	KQGP-T--------
	Brugia pahangi	VDN91899	-------N--	N	KQGP-T--------
	Acanthocheilonema viteae	VBB29420	-------N--	N	KRGP-T----R---
	Enterobius vermicularis	VDD93518	--------R-	H	KKGP-T--S-R---
	Wuchereria bancrofti	VDM08568	-------N--	N	KQGP-T----R---
	Litomosoides sigmodontis	VDK70981	-------N--	N	KRGR-T----R---
	Loa loa	XP_020303432	-------N--	N	KRGP-T----R---
	Onchocerca ochengi	VDK64311	-------N--	N	KLGP-T----R---
	Onchocerca flexuosa	OZC10900	-------N--	N	KLGP-T----R---
	Pristionchus pacificus	PDM60715	---SS---R-	H	KLGA--Y-------
	Thelazia callipaeda	VDN04565	----SI-N--	H	KRGP-T--S-R---
Outgroups (0/>200)	*Homo sapiens*	CAB45716	H-L--I--R-	D	KKENKHLAP-IR--
	Mus musculus	XP_006529331	H-L--I--Q-	D	KKENKHLAP-IR--

Supplementary Figure 28. Partial sequence alignments of a Ral guanine nucleotide dissociation stimulator protein with a CSI consisting of a 1 aa deletion (shown in black box) that is specific for the *Caenorhabditis* genus and not present in any other nematode species.

Group	Species	Accession	261		290
Caenorhabditis (6/6)	*Caenorhabditis elegans*	NP_001309573	VYTDKSGAASISIRLPPK		GDSLIFHYNLKF
	Caenorhabditis nigoni	PIC44232	-----T----V-------		------------
	Caenorhabditis brenneri	EGT39972	-----T------------		------------
	Caenorhabditis remanei	XP_003117505	-----T------------		------------
	Caenorhabditis latens	OZF84301	-----T------------		------------
	Caenorhabditis briggsae	XP_002630298	-----T----V-------		------------
Other Nematodes (0/38)	*Diploscapter pachys*	PAV72156	I---R----TVAV---V-	E	AH----------
	Ancylostoma duodenale	KIH42539	L----T--T-VA----E-	S	------------
	Ancylostoma ceylanicum	EYC03167	L----T--T-VA----E-	S	------------
	Oesophagostomum dentatum	KHJ79213	L----T--T-VA----E-	T	--T---------
	Necator americanus	XP_013296219	L----T--T-VA----E-	S	------------
	Teladorsagia circumcincta	PIO69203	L----T--T-VA----E-	S	------------
	Dictyocaulus viviparus	KJH44272	L----T--T-VA-K--D-	S	----------R-
	Haemonchus contortus	CDJ95820	L----T--T-VA----E-	P	------------
	Pristionchus pacificus	PDM62223	C---R------AVEI--E	A	A-N---------
	Loa loa	XP_020304904	-QA-R----T--VTMSNG	A	LN--------RY
	Ancylostoma caninum	RCN36404	L----T--T-VA----E-	S	------------
	Heligmosomoides polygyrus	VDO63815	L----T--T-VQ----E-	S	------------
	Nippostrongylus brasiliensis	VDL71649	L----T--T-VA----D-	S	--T---------
	Haemonchus placei	VDO42404	L----T--T-VA----E-	P	------------
	Angiostrongylus costaricensis	VDM53344	L----T--T-VA----E-	Y	--N---------
	Strongylus vulgaris	VDM79582	-T--T-VA----E- S --T---------		
	Anisakis simplex	VDK45457	ILA------T---NMCNE	A	L-----------
	Dracunculus medinensis	VDN59128	-QA-R----T---KMCSE	A	L-----------
	Enterobius vermicularis	VDD88292	-QA------T--VSMCRE	A	L-T---------
	Soboliphyme baturini	VDO95451	IFA------T-A-NMS-D	T	VGH---------
	Thelazia callipaeda	VDN04471	-KA-R----TV-VTMSNQ	A	MN---------Y
	Gongylonema pulchrum	VDN27534	LQA-R----T---TMSTE	A	LN---------
	Onchocerca ochengi	VDK64948	-KA-R----T---TMSNE	A	LN--------R-
	Litomosoides sigmodontis	VDK72628	-KA-R----T---TMSNE	A	LN--------R-
	Acanthocheilonema viteae	VBB29959	-KA-R----T--VTMSNE	A	LN--------R-
	Wuchereria bancrofti	VDM15282	-KA-R----T---TMSNE	A	LN--------R-
	Brugia pahangi	VDN85976	-KA-R----T---TMSNE	A	LN--------R-
	Strongyloides ratti	CEF60189	INS-D----T--LNMKNS	D	SKF-------R-
	Trichinella murrelli	KRX49645	-PA-QT---TVAVNMS-D	C	TR--------R-
	Trichinella nelsoni	KRX21745	-PA-QT---TVAVNMS-D	C	TR--------R-
	Trichinella pseudospiralis	KRX94659	-PA-QT---TVAVNMS-D	C	TR--------R-
	Trichinella sp. T6	KRX72173	-PA-QT---TVAVNMS-D	Y	TR--------R-
	Trichinella sp. T8	KRZ90868	-PA-QT---TVAVNMS-D	C	TR--------R-
	Trichinella spiralis	KRY34230	-PA-QT---TVAVNMS-D	C	TR--------R-
	Trichinella zimbabwensis	KRZ08997	-PA-QT---TVAVNMS-D	C	TR--------R-
	Trichuris suis	KFD55831	-PA------TVA-NMS-E	C	TKN-------R-
	Trichuris trichiura	CDW54076	-PA------TVA-NMS-E	C	TKN-------R-
	Toxocara canis	KHN77300	-LA------T---NMCSE	A	L-----------

Supplementary Figure 29. Partial sequence alignments of a Transglutaminase/ protease homolog protein with a CSI consisting of a 1 aa deletion (shown in black box) that is specific for the *Caenorhabditis* genus and not present in any other nematode species. Homologous sequences for this protein were not observed for many non-nematode organisms in the BLASTP search.

Group	Species	Accession	209		242
Caenorhabditis (6/6)	*Caenorhabditis elegans*	NP_001033544	FELQSEDMIE	S	VKLSNHTLPYMIHGLGIPYLATY
	Caenorhabditis remanei	OZG06150	-------V--	-	IR--S------------------
	Caenorhabditis latens	OZG25132	-------V--	-	IR--S------------------
	Caenorhabditis briggsae	XP_002644672	Y------E-V	N	IR--T---------------S--
	Caenorhabditis nigoni	PIC18362	Y------E-V	N	IR--T---------------S--
	Caenorhabditis brenneri	EGT30451	Y------V--	N	IR--H---------------D--
Other Nematodes (0/21)	*Diploscapter pachys*	PAV83346	YH-I---R-S		LNIPKRAQ-FQFNLI-LSSYEIH
	Ancylostoma ceylanicum	EYC37437	YL-LA--QLS		LSANRASQ-CQFLMA-L-TCES-
	Ancylostoma duodenale	KIH69031	YR-LA--QLS		LSANRTSQ-CQFLMA-L-TCES-
	Brugia malayi	XP_001898333	-S-TT--R--		MCTCDQGNLTLF-MSSV-HENV-
	Dictyocaulus viviparus	KJH48985	YR-L---RLS		LSVKANSQ--QFSLA-V-SCDS-
	Haemonchus contortus	CDJ90309	YR-LA--RLS		FSSKTFSH-CQVSMA-V-AIES-
	Loa loa	XP_020303866	-T-TT--R--		MYTCDRGNLTLF-MSSL--ENV-
	Necator americanus	XP_013308897	YR-LA--QLS		LSAK-CSQ-WQFSMA-L-SCES-
	Oesophagostomum dentatum	KHJ99848	YS-LA--RLS		LSANRSSQ-CQFSMA-L-CFES-
	Strongyloides ratti	CEF63947	-MITA--I-D		MNGVLPNQLHRLKMF----DKE-
	Wuchereria bancrofti	EJW85503	-N-TT--R--		MCTCDRGNLTLF-MSSV-HENV-
	Pristionchus pacificus	PDM64559	YD--A--R-S		LA--AR-TAAQV-LGAL-HDGL-
	Trichinella britovi	KRY47448	-SVF---I--		M-D-EMLSCHQY-FS-L-GDQL-
	Trichinella murrelli	KRX47109	-SVF---I--		M-D-EMLSCHQY-FS-L-GDQL-
	Trichinella nativa	KRZ60864	-SVF---I--		M-D-EMLSCHQY-FS-L-GDQL-
	Trichinella nelsoni	KRX17237	-SVF---I--		M-D-EMLSCHQY-FS-L-GDQL-
	Trichinella patagoniensis	KRY18874	-SVF---I--		M-D-EMLSCHQY-FS-L-GDQL-
	Trichinella pseudospiralis	KRX88958	-SVF---I--		M-D-EMLSCHQY-FS-L-GDQL-
	Trichinella sp. T6	KRX73586	-SVF---I--		M-D-EMLSCHQY-FS-L-GDQL-
	Trichinella sp. T9	KRX53517	-SVF---I--		M-D-ELLSCHQY-FS-L-GDQL-
	Trichinella spiralis	KRY28374	-SVF---I--		M-D-EMLSCHQY-FS-L-GDQL-

Supplementary Figure 30. Partial sequence alignments of a Vacuolar protein sorting-associated protein 41 homolog protein with a CSI consisting of a 1 aa insertion (shown in black box) that is specific for the *Caenorhabditis* genus and not present in any other nematode species. Homologous sequences for this protein were not observed for many non-nematode organisms in the BLASTP search

			13		36
Caenorhabditis (6/6)	*Caenorhabditis elegans*	NP_001317731	EPTYAEAHK		RPNEALLCFATPSDL
	Caenorhabditis brenneri	EGT44953	---------		---------------
	Caenorhabditis briggsae	XP_002631115	---------		---------------
	Caenorhabditis latens	OZG11344	---------		----------S-T--
	Caenorhabditis nigoni	PIC46074	---------		---------------
	Caenorhabditis remanei	XP_003117041	---------		----------S-T--
Caenorhabditis (0/36)	*Diploscapter pachys*	PAV79982	-V---D---	K	EQ---II---SRR--
	Angiostrongylus costaricensis	VDM55226	-V-F-D---	N	ER--GVV----RE--
	Haemonchus placei	VDO40680	-V-F-D---	N	ER--GVV----RE--
	Haemonchus contortus	CDJ85139	-V-F-D---	N	ER--GVV----RE--
	Teladorsagia circumcincta	PIO62909	-V-F-D---	N	ER--GVV----RE--
	Strongylus vulgaris	VDM65061	-V-F-D---	N	ER--GVV----RE--
	Heligmosomoides polygyrus	VDO83327	-V-F-D---	N	ER--GVV----RE--
	Necator americanus	XP_013301253	-V-F-D---	N	ER--GVV----RE--
	Ancylostoma ceylanicum	EYC17281	-V-F-D---	N	ER--GVV----RE--
	Nippostrongylus brasiliensis	VDL76374	-V-F-D---	N	ER--GVV----RE--
	Dictyocaulus viviparus	KJH49634	-V-F-D---	N	ER--GVV----RE--
	Pristionchus pacificus	PDM73741	-V---D---	Q	VR--GII---SR---
	Onchocerca flexuosa	VDP18596	-V-F-D---	Q	H---GIV--L-RE--
	Litomosoides sigmodontis	VDK84450	-V-F-D---	Q	H---GIV--L-RE--
	Enterobius vermicularis	VDD86392	-V---D---	E	H---GIV---SRE--
	Onchocerca ochengi	VDK67641	-V-F-D---	Q	H---GIV--L-RE--
	Loa loa	XP_020301701	-V-F-D---	Q	H---GIV--L-RD--
	Gongylonema pulchrum	VDN25083	-V-F-D---	Q	HA--GIV--L-RE--
	Brugia pahangi	VDN91487	-V-F-D---	Q	H---GIV--L-RD--
	Brugia malayi	XP_001902042	-V-F-D---	Q	H---GIV--L-RD--
	Wuchereria bancrofti	VDM20183	-V-F-D---	Q	H---GIV--L-RD--
	Dracunculus medinensis	VDN51147	-V-F-D---	Q	-A--GVV--L-RE--
	Thelazia callipaeda	VDN03402	-V-F-D---	Q	H---GIV--L-RD--
	Toxocara canis	KHN86131	-V-F-D---	Q	HA--GVV--Q-RD--
	Trichinella patagoniensis	KRY13721	-V---D--R	I	KKH-GIV---SHYE-
	Trichinella pseudospiralis	KRX92226	-V---D--R	I	KKH-GIV---SHYE-
	Trichinella papuae	KRZ74679	-V---D--R	I	KKH-GIV---SHYE-
	Trichinella nelsoni	KRX21810	-V---D--R	I	KKH-GIV---SHYE-
	Trichinella sp. T6	KRX82412	-V---D--R	I	KKH-GIV---SHYE-
	Trichinella spiralis	KRY35895	-V---D--R	I	KKH-GIV---SHYE-
	Trichinella sp. T8	KRZ90387	-V---D--R	I	KKH-GIV---SHYE-
	Trichinella sp. T9	KRX54744	-V---D--R	I	KKH-GIV---SHYE-
	Trichinella nativa	KRZ60668	-V---D--R	I	KKH-GIV---SHYE-
	Trichinella murrelli	KRX48392	-V---D--R	I	KKH-GIV---SHYE-
	Trichinella britovi	KRY51015	-V---D--R	I	KKH-GIV---SHYE-
	Trichuris suis	KFD48640	-V---D--R	A	AR--GVV--TSH-E-
Outgroups (0/>200)	*Mus musculus*	AAH26944	-V---D---	G	-K--GVIE-VSY--M
	Homo sapiens	AAA36649	-V---D---	G	-K--GVIE-VSY--M

Supplementary Figure 31. Partial sequence alignments of a Serine/arginine-rich splicing factor protein with a CSI consisting of a 1 aa deletion (shown in black box) that is specific for the *Caenorhabditis* genus and not present in any other nematode species.

Group	Species	Accession	159		191
Caenorhabditis (6/6)	*Caenorhabditis nigoni*	PIC40784	YGLACDLLWSDPA	Q	PERNGWGLSHRGISFT
	Caenorhabditis briggsae	XP_002642928	-------------	-	----------------
	Caenorhabditis elegans	NP_001249242	-------------	-	----------------
	Caenorhabditis remanei	OZF95316	-------------	-	-G--------------
	Caenorhabditis latens	OZF87542	-------------	-	-G--------------
	Caenorhabditis brenneri	EGT35952	-------V----E	S	TSNI--S--A-----S
Other Nematodes (0/47)	*Diploscapter pachys*	PAV85879	T--L--------D		KDVT---END--V---
	Ancylostoma caninum	RCN49073	--IG--------S		-Q-D--V-----L---
	Ancylostoma ceylanicum	EPB70403	--IG--------S		-Q-D--V--------
	Cylicostephanus goldi	VDK48331	--IG--------S		-Q-D--V--------P
	Necator americanus	XP_013307020	--IG--------S		-Q-D--V--------
	Oesophagostomum dentatum	KHJ94276	--IG--------S		-QKD--V--------
	Strongylus vulgaris	VDM70279	--IG--------S		-QKD--V--------
	Ancylostoma duodenale	KIH43961	--IG--------S		-Q-D--V-----LP-
	Haemonchus contortus	CDJ91010	--IG---V-----		LQ-D--V--------
	Haemonchus placei	VDO28303	--IG---V-----		LQ-D--V--------
	Heligmosomoides polygyrus	VDP38261	--IG---V-----		-H-D--T--------
	Dictyocaulus viviparus	KJH47949	--IG--------S		VQGD--I--------
	Teladorsagia circumcincta	PIO71578	--IG---V-----		LH-D--V--------
	Angiostrongylus costaricensis	VDM53877	--IG--------S		TLGD--I--------
	Toxocara canis	VDM43604	---M---V----D		EKH---SM-Q------
	Onchocerca flexuosa	OZC05533	--VM--V-----D		DRYP--A--A------
	Litomosoides sigmodontis	VDM93365	---M--I-----D		DRYP--A--A------
	Wuchereria bancrofti	EJW73884	---M--I-----D		DRYP--A--A------
	Brugia timori	VDO32279	---M--I-----D		DRYP--A--A------
	Brugia malayi	CRZ25408	---M--I-----D		DRYP--A--A------
	Brugia pahangi	VDN91264	---M--I-----D		DRYP--A--A------
	Anisakis simplex	VDK25200	---I--V-----D		DKY---A--P------
	Onchocerca ochengi	VDM94263	--MM--V-----D		DRYP--A--A------
	Acanthocheilonema viteae	VBB31930	---M--T-----D		DRYP--A--A------
	Loa loa	XP_020300975	---M--T-----D		DRYP--A--A------
	Thelazia callipaeda	VDN06638	---M--T-----D		DRYP--A--A------
	Pristionchus pacificus	PDM74400	---M---V----D		NKYP--A--T----Y-
	Soboliphyme baturini	VDP06334	F--I--I-----R		ALTM--AE-N------
	Gongylonema pulchrum	VDK49626	IR-L--------D		KDVQ---END--V---
	Radopholus similis	AZN18358	Q--L--------D		KDVQ---END--V---
	Nippostrongylus brasiliensis	VDL74770	T--L--------D		KDVT---END--V---
	Strongyloides ratti	XP_024505874	Q--L--------D		KDVT---END--V---
	Enterobius vermicularis	VDD86098	T--L--------D		KDVQ---END--V---
	Trichinella spiralis	XP_003377232	F--C--------D		-GHK--AENA--V---
	Trichinella nelsoni	KRX27798	F--C--------D		-GHK--AENA--V---
	Trichinella papuae	KRZ80860	--MC--------E		QGL---AENA--V---
	Trichinella zimbabwensis	KRZ19022	--MC--------E		QGL---AENA--V---
	Trichinella pseudospiralis	KRY91030	---C--------E		QGL---AENA--V---
	Trichinella nativa	OUC48627	F--C--------E		-GLK--AENT--V---
	Trichinella sp. T8	KRZ92852	F--C--------E		-GLK--AENT--V---
	Trichinella britovi	KRY60959	F--C--------E		-GLK--AENT--V---
	Trichinella patagoniensis	KRY19678	F--C--------E		-GLK--AENA--V---
	Trichinella sp. T6	KRX85648	F--C--------E		-GLK--AENT--V---
	Trichinella sp. T9	KRX64885	F--C--------E		-GLK--AENT--V---
	Trichinella murrelli	KRX48811	F--C--------E		-GLK--AENT--V---
	Trichuris suis	KFD70703	T--L--------D		KDVQ---END--V---
	Trichuris trichiura	CDW56051	---F---V-A--D		STIE---E-P-----
Outgroups (0/>200)	*Homo sapiens*	NP_996756	Q--L--------D		KDVQ---END--V---
	Mus musculus	BAE42366	T--L--------D		KDVQ---END--V---

Supplementary Figure 32. Partial sequence alignments of a Serine/threonine-protein phosphatase protein with a CSI consisting of a 1 aa insertion (shown in black box) that is specific for the *Caenorhabditis* genus and not present in any other nematode species.

Group	Species	Accession	761		804
Caenorhabditis homolog 1 (6/6)	*Caenorhabditis elegans*	NP_001317749	GYESMTMEQIFFHLQALYFC		RPPGGSTGELTAKDARQHYSTRVN
	Caenorhabditis brenneri	EGT34367	-F-DFGLDKLY--IA--TW-		SAREEFPL-TKLTHPHATP-F---
	Caenorhabditis briggsae	CAP35351	-FDNDNTRK---RVA--S--		A-RDTEDIDYV-QREHPTD-F---
	Caenorhabditis latens	OZF91768	-F-DEKPDKL----T--NY-		SSRSLRSLAEQLTFTHPTN-F---
	Caenorhabditis nigoni	PIC48108	-F-DLSF-KLY-RIG--KW-		S-RSPL-L-DQL-QVHATT-F---
	Caenorhabditis remanei	XP_003109959	-F-NYDI-KLY--IH--N--		A-RDT-SLDEAMGWIHPTD-F---
Caenorhabditis homolog 2 (0/6)	*Caenorhabditis elegans*	NP_506520	-FQNF-SQ-M--LAY-NNW-	S	LVRPKHYIQIILT-VHAPSKY-AM
	Caenorhabditis brenneri	EGT35769	-F-DVSDD-M--YNY-VRR-	E	TSSSPKNR--MLRRTHPTSRF---
	Caenorhabditis briggsae	CAP25386	-FQNF-SQ-M--LAY-NNW-	S	LVRPKHYIQIILT-VHAPSKY-AM
	Caenorhabditis latens	OZF96819	-FDDA-G--M--YNY-FRQ-	D	VSDPNFRP-HLKNRPHPTPRY---
	Caenorhabditis nigoni	PIC46155	HF--LSS--L--VGY-QVW-	G	AKTPETKTL-LLT-PHSPETA---
	Caenorhabditis remanei	XP_003109896	-FDDA-G--M--YNY-FRQ-	D	VSDPNFRH-HLKNRPHPTPRY---
Other Nematodes (0/27)	*Ancylostoma caninum*	AAG29103	-L-QY-PN---WITYGYSW-	M	SQTDS-LIRQLLT-VHSPG-C---
	Ancylostoma ceylanicum	AAQ75757	-L-QY-PN---WITYGFSW-	M	SQTDS-LIRQLLT-VHSPG-C---
	Ancylostoma duodenale	KIH50844	-L-QY-PN---WITYGYSW-	M	SQTDS-LIRQLLT-VHSPG-C---
	Brugia malayi	CDP96775	-L-HLD-N----LSS-QMW-	G	HSRPAALIRQVLT-QHAPLRL---
	Dictyocaulus viviparus	KJH41117	-L-QFDN--M--LGY-TVW-	G	HSTVD-LIN-ILT-SHAPQKY---
	Diploscapter pachys	PAV57490	-FQNF-S--M--LAY-NNW-	A	LIRPKYYVQ-ALT-VHSPSKY-AI
	Haemonchus contortus	AAC31568	-L-KF-PN---WISYGYSW-	A	KETQS-LVKRLLTNPHSPN-C---
	Loa loa	XP_020303789	-L-NLD-N----LSS-QTW-	G	HSRPAALIRQVLT-QHAPSRF---
	Necator americanus	XP_013303479	TT-DI-AD-L--LGWGIIL-	E	KYGEEELENAILV-NHAPSVL---
	Oesophagostomum dentatum	KHJ99552	-L-QYNN--M--LGY-TVW-	G	HSTDD-MIN-ILT-PHSPQRY---
	Onchocerca flexuosa	OZC11274	-L-HLD-D----LSS-QMW-	G	HSRPAALIRHILTGQHAPLRF---
	Pristionchus pacificus	PDM80021	-L--F-PD---WITYGGTW-	M	KLTDERLARQLMT-VHSPS-C--D
	Strongyloides ratti	CEF59415	-F-KYSND----MSY-QTW-	G	KAKPQ-VIRQILG-PHSPMRF---
	Teladorsagia circumcincta	PIO72527	-L-QYNN--M--MGY-TTW-	G	HMTKDALIN-ILT-PHSPERY---
	Toxocara canis	KHN75499	-L-HYSDD-L--INS-YTW-	A	ISRPQAVLDSLLN-GHSPHRY---
	Wuchereria bancrofti	EJW80247	-MSDF-D-----LSF-QVW-	G	HQTKEAQIKQVLTNEHSPAKY---
	Trichinella murrelli	KRX48555	-LVN--ND----LSY-NFW-	G	HKKPAAALQQVLT-PHSPEMF--I
	Trichinella nelsoni	KRX16250	-LVN--ND----LSY-NFW-	G	HKKPAAALQQVLT-PHSPEMF--I
	Trichinella papuae	KRZ70145	-LVN--ND----LSY-NFW-	G	HKKPAAALQQVLT-PHSPEMF--I
	Trichinella patagoniensis	KRY15148	-LVN--ND----LSY-NFW-	G	HKKPAAALQQVLT-PHSPEMF--I
	Trichinella pseudospiralis	KRX92259	-LVN--ND----LSY-NFW-	G	HKKPAAALQQVLT-PHSPEMF--I
	Trichinella sp. T6	KRX75996	-LVN--ND----LSY-NFW-	G	HKKPAAALQQVLT-PHSPEMF--I
	Trichinella sp. T8	KRZ86267	-LVN--ND----LSY-NFW-	G	HKKPAAALQQVLT-PHSPEMF--I
	Trichinella sp. T9	KRX55739	-LVN--ND----LSY-NFW-	G	HKKPAAALQQVLT-PHSPEMF--I
	Trichinella spiralis	XP_003372438	-LVN--ND----LSY-NFW-	G	HKKPAAALQQVLT-PHSPEMF--I
	Trichinella zimbabwensis	KRZ10628	-LVN--ND----LSY-NFW-	G	HKKPAAALQQVLT-PHSPEMF--I
	Trichuris trichiura	CDW59783	SL-QF--D----LSF-NFY-	G	NSTNMFLHKMININEHAPDR---V

Supplementary Figure 33. Partial sequence alignments of NEPrilysin metallopeptidase family protein with a CSI consisting of a 1 aa deletion (shown in black box) that is specific for the *Caenorhabditis* genus and not present in any other nematode species. There are two homologs of this protein in the *Caenorhabditis* species, one of which contains the CSI.

Group	Species	Accession	224		262
Caenorhabditis (6/6)	*Caenorhabditis elegans*	NP_001251923	CPFRHSEKQVLKQKLGSDAI	L	KKEQIERIAELAELNQYD
	Caenorhabditis brenneri	EGT35184	-------IAL---R-S-EEV	-	---HV--------A----
	Caenorhabditis briggsae	XP_002640609	---------------S-ESV	-	----VD--S---------
	Caenorhabditis nigoni	PIC54883	---------------S-ESV	-	----VD--S---------
	Caenorhabditis remanei	OZF76862	---------------SGES-	-	--D-V-L-VD---S----
	Caenorhabditis latens	OZG16059	---------------AGES-	-	--D-VDL-VD---S----
Other Nematodes (0/27)	*Ancylostoma ceylanicum*	EYB89256	---K-LDH-L-A-R-EK-GL		NRN-VNQ-VNYSKASA--
	Ancylostoma duodenale	KIH58777	---K-LDH-L-A-R-EK-GL		NRN-VNQ-VNYSKASA--
	Brugia malayi	XP_001901741	--Y---DLRL-T-N-E-SGL		S-S---H-TL-SKNK---
	Carlito syrichta	XP_008068171	------DPEL-----Q-YK-		SPGG-SQ-LD-VKGTH-Q
	Dictyocaulus viviparus	KJH47173	---K-VHHPQ-AER-EKKGL		NTD--NH-INYSKA-AF-
	Diploscapter pachys	PAV61350	---A-M-TAL-T-R-KNCD-		SDVEV-N-VGFSKTR---
	Haemonchus contortus	CDJ95440	---K-LDN-L-A-R-EK-GL		NR---SQ-VNYTKT-AF-
	Loa loa	XP_020307065	--Y---DL-P-V-N-R-SGL		S-S---H-TL-SKNT---
	Necator americanus	XP_013292330	---K-LDH-L-A-R-EK-GL		NRD--SQ-VNYSKISA--
	Oesophagostomum dentatum	KHJ79721	---K-LDQ-L-A-R-QK-GL		NAN--NQ-VNYSKASA--
	Onchocerca flexuosa	OZC08232	--Y--NDL-S-A-N-ENSGL		S-PKS-H-IL-SKNT---
	Pristionchus pacificus	PDM67073	---K--TEDN--SI-KANK-		-S-D-DEMIA-SKI--F-
	Strongyloides ratti	CEF65434	---K--D-RN-IHL-RNMGL		TT--VDVVL-QIKT-RF-
	Toxocara canis	KHN76917	------DAT--A-M-EKVGL		L-HE-AT-VD-SKSS---
	Wuchereria bancrofti	EJW88833	--Y---DLRL-A-N-E-SGL		P-S---H-TL-SKN----
	Trichinella papuae	KRZ77714	---K-CDAEH-Q-L-KNCG-		H-DD-KN-VSY-SN-H-N
	Trichinella pseudospiralis	KRZ28229	---K-CDAEH-Q-L-KNCG-		H-DD-KN-VNY-SN-H-N
	Trichinella sp. T9	KRX62449	---K-CDAEH-Q-L-KNCG-		H-DN-KN-VNY-SN-H-N
	Trichinella zimbabwensis	KRZ08210	---K-CDAEH-Q-L-KNCG-		H-DD-KN-VSY-SN-H-N
	Trichinella nativa	OUC41177	---K-CDAEH-Q-L-KNCG-		H-DN-KN-VNY-SN-H-N
	Trichinella sp. T6	KRX74683	---K-CDAEH-Q-L-KNCG-		H-DN-KN-VNY-SN-H-N
	Trichinella britovi	KRY25348	---K-FDAEH-Q-L-KNCG-		H-DN-KN-VNY-SN-H-N
	Trichinella nelsoni	KRX15057	---K-CDAEH-Q-L-KNCG-		H-DD-KN-VNY-SN-H-N
	Trichinella spiralis	KRY40501	---K-CDAEH-Q-L-KNCG-		H-DD-KN-VNY-SN-H-N
	Trichoplax adhaerens	XP_002114281	-----TDPDL-R-R-L-NGL		SS-GAKE-T---KGSH-Q
	Trichuris suis	KFD55183	---K-CDT---R-L-TEWGI		G-DDA-K-V-FSQWKR--
	Trichuris trichiura	CDW57123	---K-CDV---R-L-TEWGI		G-DDA-K-V-FSQWKR--
Outgroups (0/>100)	*Homo sapiens*	XP_016866496	------DPEL-----Q-YK-		SP GG-SQ-LD-VKGTH-
	Mus musculus	XP_011236757	------DAEL----MQ-YK-		PA SG-SQ-LD-VKG-H-

Supplementary Figure 34. Partial sequence alignments of a DNA PRImase homolog protein with a CSI consisting of a 1 aa insertion (shown in black box) that is specific for the *Caenorhabditis* genus and not present in any other nematode species.

Group	Species	Accession	56		91
Caenorhabditis (6/6)	*Caenorhabditis elegans*	NP_001252372	SSGIDGRLVASATGRF		LGNMRHSYFANRNVMALKRR
	Caenorhabditis brenneri	EGT32311	-C-V--------S--L		-R-IH-PF----SI-S----
	Caenorhabditis latens	OZG16099	---V----L---S--L		-K-NHLPF-----L-T----
	Caenorhabditis briggsae	XP_002640718	-C-V-----------L		-K--H-PF-----LLQ----
	Caenorhabditis remanei	OZF76907	-C-V----L---S--L		-K-NHLPF-----L------
	Caenorhabditis nigoni	PIC54820	-C-V-----------L		-K--H-PF-----LLQ----
Other Nematodes (0/24)	*Loa loa*	XP_020306018	-A-A----QV-LN--L	AAV	GSEKDFLFN---V-LT-L-K
	Wuchereria bancrofti	EJW81713	-A-A----QV-LN--L	AAI	GSEKDFVFN-C-V-LT-V-K
	Onchocerca flexuosa	VDO78063	-V-G----QV-LN--L	AAV	GSEKDFLFN-CQV-LT-I-K
	Acanthocheilonema viteae	VBB34527	-A-A----QV-LN--L	AAV	GSERDFLFS-C-V-LT-L-K
	Litomosoides sigmodontis	VDM91432	-A-A----QV-LN--L	AAV	GSQKDFLFS-C-V-LT-V-K
	Brugia pahangi	VDN83211	-A-A----QV-LN--L	AAI	RSEKDFVFN-C-V-LT-M-K
	Brugia timori	VDO23402	-A-A----QV-LN--L	AAI	RSEKDFVFN-C-V-LT-V-K
	Onchocerca ochengi	VDK89390	-V-G----QV-LN--L	AAV	GSEKDFLFN-CQV-LTVI-K
	Thelazia callipaeda	VDM97139	-V-A----QI-LN--L	VAS	GSEKDFGFSVCQV--TVVKK
	Gongylonema pulchrum	VDN40914	-V-G----II-QN--L	APH	ESDNDL-FN-C-V--T-L-K
	Dracunculus medinensis	VDN51116	-V-G-----V-LN--I	VPH	GTQRDVFFK-C-VL-TVI-K
	Toxocara canis	VDM40044	TV-A----EV-LN-HI	VPR	HAQIDFPFNSC-VA-T-V-K
	Angiostrongylus costaricensis	VDM56220	-C-V----LL--N--L	ATF	GSS-DFAFSVLKSAL-IT--
	Dictyocaulus viviparus	KJH41983	-C-V----LL--N--L	VTF	GST-DYGFSVL-SSLTIS--
	Strongylus vulgaris	VDM65762	-C-A--K-LL-TN--L	VTT	CHIKDYGFSLLKTALT-V-K
	Nippostrongylus brasiliensis	VDL81967	-C-V----LL--N--L	ATF	ASAVD-PFCFVKAALT-T--
	Haemonchus contortus	CDJ92092	-C-V----LL-SN--L	VTF	ASPCDYGFSLMKSSLT-T--
	Haemonchus placei	VDO35340	-C-V----LL-SN--L	VTF	ASPCDYGFSLMKSSLT-T--
	Ancylostoma duodenale	KIH63320	-C-L--KVLL-TN--L	VTI	ANTVDFGFSLLKTALTIV--
	Ancylostoma ceylanicum	EYB93854	-C-L--KVLL-TN--L	VTI	ANTVDFGFSLLKTALTIV--
	Ancylostoma caninum	RCN47650	-C-L--KVLL-TN--L	VTI	ANTVDFGFSLLKTALTIV--
	Pristionchus pacificus	PDM66927	TA-A---MEL-MN-KV	VPR	GCGQDL-VNLHTEPIL-I-
	Enterobius vermicularis	VDD92248	TV-A--Q-NL-L---L	LPA	CKQRQFNFGGI-TLLS--KK
	Oesophagostomum dentatum	KHJ81200	TC-A --K-LL--N-L	VTP	SHTSDYGFSLLKTALCIV--

Supplementary Figure 35. Partial sequence alignments of a Probable maleylacetoacetate isomerase protein with a CSI consisting of a 3 aa deletion (shown in black box) that is specific for the *Caenorhabditis* genus and not present in any other nematode species. Homologous sequences for this protein were not observed for many non-nematode organisms in the BLASTP search.

			39		61
Caenorhabditis Homolog 1 (5/5)	*Caenorhabditis elegans*	NP_001254102	VVYLYQFPR	P	ANRQPNLSPYCLK
	Caenorhabditis brenneri	EGT46528	---------	-	---------F---
	Caenorhabditis briggsae	XP_002630778	---------	-	---L---------
	Caenorhabditis nigoni	PIC46704	---------	-	---L-----F---
	Caenorhabditis remanei	XP_003108831	---------	-	--KL-----F---
Caenorhabditis Homolog 2 (0/6)	*Caenorhabditis brenneri*	EGT48831	---------		CRLL----S--M-
	Caenorhabditis elegans	NP_496813	---------		-RLL-----F-M-
	Caenorhabditis latens	OZG18599	---------		TRLL-----F-M-
	Caenorhabditis nigoni	PIC26241	---------		I-TV----S----
	Caenorhabditis briggsae	CAP37749	---------		TRVL----TF-M-
	Caenorhabditis remanei	XP_003102439	---------		TRLL-----F-M-
Other Nematodes (0/41)	*Diploscapter pachys*	PAV87405	T---F----		TQHL-----F---
	Brugia malayi	XP_001898570	I------N-		-SSS--I--F-F-
	Ancylostoma ceylanicum	EPB73791	T--------		SKAL--V--F---
	Ancylostoma duodenale	KIH57073	T--------		SPVL-----F-M-
	Ascaris suum	ERG82980	-------K-		SPIM--I--F---
	Dictyocaulus viviparus	KJH50494	---------		CKEI-----F---
	Haemonchus contortus	CDJ97726	I--------		-RSV----T----
	Loa loa	XP_003138099	-I-----S-		-SSS--I--F-F-
	Necator americanus	XP_013293585	T--------		SPVL-----F---
	Oesophagostomum dentatum	KHJ90324	T--------		SKYV--V--F---
	Onchocerca flexuosa	OZC11826	-------N-		-SSS--I--F-F-
	Pristionchus pacificus	PDM61759	I--------		SPIL---------
	Strongyloides ratti	CEF67762	-------K-		TPAL--M--F---
	Wuchereria bancrofti	EJW83270	-------N-		-SSS--I--F-F-
	Teladorsagia circumcincta	PIO65880	--------		PKML---------
	Haemonchus placei	VDO62328	I--------		-KSV---------
	Pristionchus pacificus	PDM61759	I--------		SPIL---------
	Strongylus vulgaris	VDM84836	I--------		-KTV--I------
	Heligmosomoides polygyrus	VDP08595	--------		SPVM-----F---
	Ancylostoma ceylanicum	EPB76689	--------		SPVL-----F---
	Dictyocaulus viviparus	KJH50494	---------		CKEI-----F---
	Toxocara canis	KHN81881	---------		VKCI-----F---
	Nippostrongylus brasiliensis	VDL85117	---------		SPTV--V--F---
	Angiostrongylus costaricensis	VDM52956	---------		SKTI--M--F---
	Acanthocheilonema viteae	VBB33885	--------		VKHI--I--F---
	Anisakis simplex	VDK43584	I--------		SKGI-S---F---
	Gongylonema pulchrum	VDK37804	---------		-KCI-SQ--F---
	Thelazia callipaeda	VDN01285	-------G-		S-SA--I--F-F-
	Onchocerca ochengi	VDK63043	-------N-		-SSS--I--F-F-
	Wuchereria bancrofti	EJW83270	-------N-		-SSS--I--F-F-
	Litomosoides sigmodontis	VDK72690	I------S-		-SSS--I--F-F-
	Loa loa	XP_003138099	-I-----S-		-SSS--I--F-F-
	Enterobius vermicularis	VDD85294	---------		VKYV-SF--F---
	Soboliphyme baturini	VDP18697	---F----		SSFI--I--F---
	Brugia pahangi	VDN88413	I------N-		-SSS--I--F-F-
	Brugia malayi	CTP81381	I------N-		-SSS--I--F-F-
	Brugia timori	VDO23595	I------N-		-SSS--I--F-F-
	Ancylostoma caninum	RCN39977	---------		TKIL-SP-AP---
	Trichinella spiralis	XP_003373650	---F-P--		STYI--I--FS--
	Trichinella zimbabwensis	KRZ10264	---F-P--		STYI--I--FS--
	Trichinella pseudospiralis	KRX89604	---F-P--		STYI--I--FS—

Supplementary Figure 36. Partial sequence alignments of a Glutathione S-transferase protein with a CSI consisting of a 1 aa insertion (shown in black box) that is specific for the *Caenorhabditis* genus and not present in any other nematode species. There are two homologs of this protein in the *Caenorhabditis* species, one of which contains the CSI. The sequence alignment for homolog 1 of the species *Caenorhabditis latens* was not observed in the BLASTP search.

Group	Species	Accession	32		52
Caenorhabditis (6/6)	*Caenorhabditis elegans*	NP_001254124	KYQVVKYH	S	NIPMSPLTTHRL
	Caenorhabditis briggsae	XP_002646742	--------	-	------------
	Caenorhabditis nigoni	PIC45241	--------	-	------------
	Caenorhabditis remanei	XP_003113789	-----R--	-	------------
	Caenorhabditis latens	OZG16232	--------	-	------------
	Caenorhabditis brenneri	EGT55705	--------	-	K-----------
Other Nematodes (0/30)	*Diploscapter pachys*	PAV72616	-K---R-P		IV-L----A---
	Ancylostoma ceylanicum	EYC06479	----I---		HV-C----A---
	Ancylostoma duodenale	KIH68239	----I---		HV-C----A---
	Brugia malayi	XP_001896433	-R--IR-E		YV-L---SA---
	Dictyocaulus viviparus	KJH48055	--------		HV-C----A---
	Haemonchus contortus	CDJ98226	--------		HV-C----A---
	Oesophagostomum dentatum	KHJ95417	----I---		RV-C----A---
	Necator americanus	XP_013301552	----I---		HV-C----A---
	Pristionchus pacificus	PDM61696	-R---T-E		H--L----A---
	Wuchereria bancrofti	EJW74639	-R--IR-E		YV-L---SA---
	Haemonchus placei	VDO36023	--------		HV-C----A---
	Heligmosomoides polygyrus	VDP18530	--------		HV-C----A---
	Nippostrongylus brasiliensis	VDL81814	--------		HV-C----A---
	Ancylostoma caninum	RCN34876	----I---		HV-C----A---
	Angiostrongylus costaricensis	VDM60974	-------D		HV-C----A---
	Dracunculus medinensis	VDN60070	-R---R-Q		R--L----A---
	Soboliphyme baturini	VDP07701	Q----R-V		AV-L----SQ--
	Thelazia callipaeda	VDN02079	-R---R-Q		YV-L---SA---
	Litomosoides sigmodontis	VDK68374	-H--IR-E		Y--L---SA---
	Enterobius vermicularis	VDD95055	-R-----D		FL-L---SA---
	Onchocerca flexuosa	VDP20800	-R--IR-T		YV-L---SA---
	Onchocerca ochengi	VDK83226	-R--IR-A		YV-L---SA---
	Acanthocheilonema viteae	VBB25206	-H--IR-E		YV-L---SA---
	Brugia timori	VDO42288	-R--IR-E		YV-L---SA---
	Brugia pahangi	VDN84995	-R--IR-E		YV-L---SA---
	Loa loa	XP_003142184	-R--IR-E		YV-L---SA---
	Brugia malayi	XP_001896433	-R--IR-E		YV-L---SA---
	Anisakis simplex	VDK20128	-R---R-E		CV-L---SAR--
	Trichuris suis	KFD58678	QH--IR-V		H--L--ISDQ--
	Trichuris trichiura	CDW58322	QH--IR-V		D--L--VSDQ--
Outgroups (0/>100)	*Homo sapiens*	NP_056158	Q--T-R-D		IL-L--VSRN--
	Mus musculus	NP_080293	Q--T-R-D		IL-L---SRN--

Supplementary Figure 37. Partial sequence alignments of a CTD nuclear envelope phosphatase 1 homolog protein with a CSI consisting of a 1 aa insertion (shown in black box) that is specific for the *Caenorhabditis* genus and not present in any other nematode species.

Group	Species	Accession	208		248
Caenorhabditis (6/6)	*Caenorhabditis elegans*	NP_506895	MYIFGGRSDESGQ	VGDEHL	FHTIHDQYDDTLMALNLATGAW
	Caenorhabditis brenneri	EGT59124	---------QM--	------	---TQ-L----------S--V-
	Caenorhabditis latens	OZG18972	---------HN--	------	---TQ-L----------T--V-
	Caenorhabditis remanei	OZF77388	---------HN--	------	---TQ-L----------T--V-
	Caenorhabditis nigoni	PIC29033	---------HN--	------	---TQ-L---S----D-E-QE-
	Caenorhabditis briggsae	XP_002637963	---------HN--	------	---TQ-L---S----D-E-QE-
Other Nematodes (0/33)	*Diploscapter pachys*	PAV83173	--V------IT-E		--STR-T---R----D-K--L-
	Heligmosomoides polygyrus	VDO86832	---------YT--	-SET	--STR-M--ER-K--D-R--E-
	Ancylostoma duodenale	KIH61473	---------HT--		--STR-M--ER-K--D-R--E-
	Ancylostoma ceylanicum	EYB92376	---------HT--		--STR-M--ER-K----R--E-
	Strongylus vulgaris	VDM79853	---------HT--		--STR-M--ER-K--D-R--E-
	Dictyocaulus viviparus	KJH53729	---------HT--		--STR-M--ER-K--D-R--E-
	Ancylostoma caninum	RCN28697	---------HT--		--STR-M--ER-K--D-R--E-
	Necator americanus	XP_013307245	---------HT--		--STR-M--ER-K--D-R--E-
	Oesophagostomum dentatum	KHJ87750	---------HT--		--STR-M--ER-K--D-R--E-
	Angiostrongylus costaricensis	VDM62307	---------HT--		--STR-M--ER-K--D-R--E-
	Haemonchus contortus	CDJ94868	---------YT--		--STR-M--ER-K--D-R--E-
	Teladorsagia circumcincta	PIO68211	---------YT--		--STR-M--ER-K--D-R--E-
	Nippostrongylus brasiliensis	VDL81029	---------YT--		--STR-M--ER-K--D-R--E-
	Haemonchus placei	VDO35379	---------YT--		--STR-M--ER-K--D-R--E-
	Wuchereria bancrofti	EJW80092	---------LH-A		--SSR-Y-S-V-KV---K--R-
	Brugia timori	VDO43970	---------LH-A		--SSR-Y-S-V-KV---K--R-
	Loa loa	XP_003148138	---------LQ-A		--SSR-Y-S-V-KV---K--R-
	Brugia pahangi	VDN94587	---------LH-A		--SSR-Y-S-V-KV---K--R-
	Brugia malayi	XP_001895672	---------LH-A		--SSR-Y-S-V-KV---K--R-
	Litomosoides sigmodontis	VDK89662	---------LH-A		--SSR-Y-S-V-KV---K--R-
	Acanthocheilonema viteae	VBB30887	--V------LH-A		--SSR-Y-S-V-KV---K--H-
	Anisakis simplex	VDK18725	---------QL--		--SSR-F-CKR-KV-D-KKFE-
	Toxocara canis	VDM42343	--V------QL--		--SSR-L-C-R-KV-D-K-TQ-
	Trichinella spiralis	KRY37343	--V-------A-P		H-SNVER-PTD-FY-D-S-FE-
	Trichinella murrelli	KRX46935	--V-------A-P		H-SNVER-PTD-FY-D-S-FE-
	Trichinella britovi	KRY52927	--V-------A-P		H-SNVER-PTD-FY-D-S-FE-
	Trichinella patagoniensis	KRY19859	--V-------A-P		H-SNVER-PTD-FY-D-S-FE-
	Trichinella nativa	OUC43113	--V-------A-P		H-SNVER-PTD-FY-D-S-FE-
	Trichinella nelsoni	KRX18119	--V-------A-P		H-SNVER-PTD-FY-D-S-FE-
	Trichinella sp. T6	KRX81102	--V-------A-P		H-SNVER-PTD-FY-D-S-FE-
	Trichinella sp. T8	KRZ93130	--V-------A-P		H-SNVER-PTD-FY-D-S-FE-
	Trichuris suis	KHJ46275	--V---------P		---NSEL-NTE--Y-DF-DNS-
	Trichuris trichiura	CDW55263	--V---------P		---NSEL-NTE--Y-DFTDNS-
Outgroups (0/>100)	*Homo sapiens*	AAH41793	--V----A-RF-P		--SNNEI-CNRIRVFDTR-E--
	Mus musculus	BAB23704	--V----A-RF-P		--SNNEI-CNRIRVFDTR-E--

Supplementary Figure 38. Partial sequence alignments of a Kelch-domain protein with a CSI consisting of a 6 aa insertion (shown in black box) that is specific for the *Caenorhabditis* genus and not present in any other nematode species.

Group	Species	Accession	946 – 983
Caenorhabditis homolog 1 (0/6)	*Caenorhabditis elegans*	NP_741705	LAEVEGRNFFLERLIDDLKNN [ ] EEAKFFEISLAERDAQI
	Caenorhabditis latens	OZG22582	------------K--E--R-- [ ] --S-M-------K----
	Caenorhabditis nigoni	PIC15211	------------K--E--R-- [ ] --S-LY------K----
	Caenorhabditis remanei	POM49663	------------K--E--R-- [ ] --S-M-------K----
	Caenorhabditis briggsae	XP_002643265	------------K--E--R-- [ ] --S-LY------K----
	Caenorhabditis brenneri	EGT43922	------*-----K--E----- [ ] --S-LY----S-K---- *RVSIPFEGKKSIIIQ
Caenorhabditis homolog 2 (0/6)	*Caenorhabditis remanei*	POM51826	-SDL----VL--KQ-E--NYQ LE DDQRSY-AA-NDK----
	Caenorhabditis latens	OZG23421	-SDL----VL--KQ-E--NYQ LE DDQRSY-AA-NDK----
	Caenorhabditis brenneri	EGT30317	M-DL----LL--KQVE--NYQ ME -DMRSY-Q--NDK-SS-
	Caenorhabditis nigoni	PIC18245	M-DL----LL--KQVE--NYQ ME -DMRTY-Q--NDK-SS-
	Caenorhabditis briggsae	XP_002644610	M-DL----LL--KQVE--NYQ ME -DMRTY-Q--NDK-SS-
	Caenorhabditis elegans	NP_508836	M-DL----LL--KQ-E--NYQ ME -DMRSY-Q--NDK-TS-
Other Nematodes (0/49)	*Ancylostoma ceylanicum*	EYC23157	--NL----HI--K--Q--NYQ ID D-SRVTMAH-S-K-DE-
	Ancylostoma caninum	RCN38944	--NL----HI--K--Q--NYQ ID D-SRVTMAH-S-K-DE-
	Oesophagostomum dentatum	KHJ96139	--NL----HI--K--Q--NYQ ID D-SRVTMAH-S-K-DE-
	Cylicostephanus goldi	VDN25456	--NL----HI--K--Q--NYQ IE D-SRVTMAH-S-K-DE-
	Angiostrongylus costaricensis	VDM56448	--NL----HI--K--Q--NFQ IE D-SRVTMAH-S-K-DE-
	Dictyocaulus viviparus	KJH42959	--NL----HI--K--Q--NFQ IE D-SRVTMAH-S-K-DE-
	Necator americanus	XP_013302480	--NL----HI--K--Q--NYQ IE D-SRVTMAH-S-K-DE-
	Haemonchus contortus	CDJ82493	--NL----HI--K--Q--TYQ IE D-NRVSMAH-S-K-DE-
	Strongyloides ratti	XP_024507819	--DL----AL--KQ-E--NYQ IE DDQRSY-AA-N-K-V--
	Diploscapter pachys	PAV81200	--DL----AM--KQ-QE-NYQ LE DDQRSY-AA-NDK----
	Dracunculus medinensis	VDN53013	--DL----AL--KQ-QE-NYQ LE DDQRSY-AA-ND-----
	Nippostrongylus brasiliensis	VDL80648	--DL----AL--KQ-QE-NYQ LE DDQRSY-AA-ND-----
	Gongylonema pulchrum	VDN18649	--DL----SL--KQ-QE-NYQ LE DDQRSY-AA-ND-----
	Haemonchus placei	VDO26827	--DL----AL--KQ-QE-NYQ LE DDQRSY-AA-ND-----
	Ancylostoma duodenale	KIH69140	--DL----AL--KQ-QE-NYQ LE DDQRSY-AA-ND-----
	Teladorsagia circumcincta	PIO76169	--DL----AL--KQ-QE-NYQ LE DDQRSY-AA-ND-----
	Loa loa	XP_020304756	--DL----SL--KQMQE-NYQ LE DDQRSY-AA-ND--S--
	Wuchereria bancrofti	VDM16573	--DL----SL--KQMQE-NYQ LE DDQRSY-AA-ND-----
	Brugia malayi	CRZ23584	--DL----SL--KQMQE-NYQ LE DDQRSY-AA-ND-----
	Onchocerca volvulus	AAC41561	--DL----SL--KQMQE-NYQ LE DDQRSY-AA-ND-----
	Litomosoides sigmodontis	VDK86494	--DL----SL--KQMQE-NYQ LE DDQRSY-AA-ND-----
	Onchocerca flexuosa	OZC09651	--DL----SL--KQ-QE-NFQ LE DDQRSY-AA-ND-----
	Thelazia callipaeda	VDN06362	--DL----SL--KQ-QE-NFQ LE DDQRSY-AA-ND-----
	Onchocerca ochengi	VDK87566	--DL----SL--KQMQE-NYQ LE DDQRSY-AA-ND-----
	Setaria digitata	BAU25821	--DL----SL--KQMQE-NYQ LE DDQRSY-AA-ND-----
	Acanthocheilonema viteae	VBB27072	--DL----SL--KQ-QE-NFQ LE DDQRSY-AA-ND-----
	Dirofilaria immitis	AAF37702	--DL----SL--KQ-QE-NFQ LE DDQRSY-AA-ND-----
	Brugia timori	VDO34026	--DL----SL--KQ-QE-NFQ LE DDQRSY-AA-ND-----
	Anisakis simplex	VDK61084	--DL----AL--KQ-QE-NFQ LE DDQRSY-AA-ND-----
	Heligmosomoides polygyrus	VDO88828	-SDL----AL--KQ-QE-NYQ LE DDQRSY-AA-ND-----
	Ascaris lumbricoides	CAA60046	--DL----AL--KQ-QE-NFQ LE DDQRSY-AA-ND-----
	Toxocara canis	KHN74012	--DL----AL--KQ-QE-NFQ LE DDQRSY-AA-ND-----
	Brugia pahangi	VDN89362	--DL----SL--KQ-QE-NFQ LE DDQRSY-AA-ND-----
	Enterobius vermicularis	VDD86780	--DL----SL--KQTQE-NYQ LE DDQRSY-AA-ND-----
	Pristionchus pacificus	PDM84472	--DL----AL--KQTQE-NYQ LE DDQRSY-AA-ND---S-
	Trichuris suis	KFD55367	--DL-S--SL--KQVQE-TYQ LE DDQRQY-SA-ND-----
	Trichuris trichiura	CDW54395	--DL-S--SL--KQVQE-TYQ LE DDQRQY-SA-ND-----
	Trichinella patagoniensis	KRY09282	-GDL-S--SL--KQVQE-TYQ LE DDQRQY-SA-ND--G--
	Trichinella sp. T6	KRX71243	-GDL-S--SL--KQVQE-TYQ LE DDQRQY-SA-ND--G--
	Trichinella spiralis	KRY27589	-GDL-S--SL--KQVQE-TYQ LE DDQRQY-SA-ND--G--
	Trichinella britovi	KRY45949	-GDL-S--SL--KQVQE-TYQ LE DDQRQY-SA-ND--G--
	Trichinella papuae	KRZ68635	-GDL-S--SL--KQVQE-TYQ LE DDQRQY-SA-ND--G--
	Trichinella zimbabwensis	KRZ06996	-GDL-S--SL--KQVQE-TYQ LE DDQRQY-SA-ND--G--
	Trichinella sp. T9	KRX53078	-GDL-S--SL--KQVQE-TYQ LE DDQRQY-SA-ND--G--
	Trichinella murrelli	KRX38326	--DL-SK-VM--KQVQE-NHQ LE DDQRQY-MA-ND----L
	Trichinella nelsoni	KRX15494	--DL-SK-VM--KQVQE-NHQ LE DDQRQY-MA-ND----L
	Trichinella sp. T8	KRZ90607	--DL-SK-VM--KQVQE-NHQ LE DDQRQY-MA-ND----L
	Trichinella nativa	KRZ52411	--DL-SK-VM--KQVQE-NHQ LE DDQRQY-MA-ND----L
	Trichinella pseudospiralis	KRX90006	--DL-SK-VM--KQVQE-NHQ LE DDQRQY-MA-ND----L
Outgroups (0/>100)	*Homo Sapiens*	NP_003371	VDALK-T-ES---QMREMEE- FA V--ANYQDTIGRLQDE-
	Mus musculus	NP_001124492	-ESLR-T-ES---QMREQEER HA R-SASYQEA--RLEEEG

Supplementary Figure 39. Partial sequence alignments of an Intermediate filament protein with a CSI consisting of a 2 aa deletion (shown in black box) that is specific for the *Caenorhabditis* genus and not present in any other nematode species. There are two homologs of this protein in the *Caenorhabditis* species, one of which contains the CSI.

			87		130
Rhabditoidea (7/7)	*Caenorhabditis elegans*	NP_001255355	YVGNLLWYTTDADLLKALQSTGLA	RS	QFADMKFFENRTNGQSKG
	Caenorhabditis nigoni	PIC32931	-I---------------IA----S	--	------------------
	Caenorhabditis briggsae	XP_002632965	-I---------------IA----S	--	------------------
	Caenorhabditis remanei	POM45433	--------------M--IST----	--	------------------
	Caenorhabditis latens	OZG15436	--------------M--IST----	--	------------------
	Caenorhabditis brenneri	EGT39303	--------------M--II----P	--	------------------
	Diploscapter pachys	PAV62065	----MD-F---EY--QQ-RAI--D	M-	SLV----HD--I----R-
Other Nematodes (1/42)	*Nippostrongylus brasiliensis*	VDL74792	-I---V-WA----VALQIK-I--T		DLI---------------
	Dictyocaulus viviparus	KJH44095	-I---V-WA----VALHIKAI-IT		DLI---------------
	Pristionchus pacificus	PDM70858	-I--MS-W---D--V-VIT-C-VD		DLV-L--Y---L------
	Haemonchus contortus	CDJ96265	-I---V-WA----VALHVKQL-IT		DLI---------------
	Heligmosomoides polygyrus	VDP39109	-I---V-WA---- ILHVKAI-V-		DLI---------------
	Necator americanus	XP_013296525	-I---V-WA----VGLHIKAI-IP		DLI---------------
	Ancylostoma duodenale	KIH43897	-I---V-WA----VGLHIKAL-IP		DLI---------------
	Ancylostoma ceylanicum	EYC08983	-I---V-WA----VGLHIKAL-IP		DLI---------------
	Ancylostoma caninum	RCN53282	-I---V-WA----VGLHIKAL-IP		DLI---------------
	Oesophagostomum dentatum	KHJ89953	-I---V-WA----VGLHIKAL-IP		DLI---------------
	Enterobius vermicularis	VDD90720	-I--MT-W---V--QTLIL-C-VS		DLI-I--Y---N------
	Brugia timori	VDO15610	-I--MT-W---V--QTMIL-C-AT		NLI-I--Y---N------
	Anisakis simplex	VDK46003	-I--MT-W---V--QTVIL-C-A-		DLI-L--Y---N------
	Wuchereria bancrofti	VDM07655	-I--MT-W---V--QTMIL-C-AT		NLI-I--Y---N------
	Brugia malayi	CDQ06651	-I--MT-W---V--QTMIL-C-AT		NLI-I--Y---N------
	Brugia pahangi	VDN82590	-I--MT-W---V--QTMIL-C-AT		NLI-I--Y---N------
	Loa loa	XP_020302758	-I--MT-W---V--QTMIL-C-AT		NLI-I--Y---N------
	Toxocara canis	KHN77344	-I--MT-W---V--QTLIL-C-AS		DLI-I--Y---N------
	Onchocerca flexuosa	OZC07263	-I--MT-W---V--QTMIL-C-AT		NLI-I--Y---N------
	Onchocerca ochengi	VDK75787	-I--MT-W---V--QTMIL-C-AT		NLI-I--Y---N------
	Thelazia callipaeda	VDM99214	-I--MT-W---V--QTMIL-C-AT		NLI-I--Y---N------
	Acanthocheilonema viteae	VBB30190	-I--MT-W---V--QTIIL-C-AT		NLI-I--Y---N------
	Litomosoides sigmodontis	VDK74201	-I--MT-W---V--QTIIL-C-AT		NLI-I--Y---N------
	Gongylonema pulchrum	VDN31613	----MT-W---V--QTMIL-C-AT		NLI-I--Y---N------
	Dracunculus medinensis	VDN55947	-I--MT-W---V--QSLIL-V-AT		DLI-I--Y---N----R-
	Soboliphyme baturini	VDP06154	-----T-W---Q--AD-VA-INVT		DLV-I--Y---A------
	Strongyloides ratti	XP_024502417	-IS--T-W---D--SEFVIQQ-CE		TPL-I--H-YKN------
	Trichinella patagoniensis	KRY14057	-----T-W--YF--Q-----I-IT		DLVEVR-N---A------
	Trichinella britovi	KRY57026	-----T-W--YF--QN----I-IT		DLVEVR-N---A------
	Trichinella sp. T6	KRX77213	-----T-W--YF--QN----I-IT		DLVEVR-N---A------
	Trichinella nelsoni	KRX21924	-----T-W--YF--QN----I-IT		DLVEVR-N---A------
	Trichinella spiralis	KRY39760	-----T-W--YF--QN----I-IT		DLVEVR-N---A------
	Trichinella murrelli	KRX49284	-----T-W--YF--QN----I-IT		DLVEVR-N---A------
	Trichinella papuae	KRZ74509	-----T-W--YF--QN----I-IT		DLVEVR-N---A------
	Trichinella pseudospiralis	KRX86329	-----T-W--YF--QN----I-IT		DLVEVR-N---A------
	Trichinella nativa	KRZ57179	-----T-W--YF--QN----I-IT		DLVEVR-N---A------
	Trichinella sp. T9	KRX60839	-----T-W--YF--QN----I-IT		DLVEVR-N---A------
	Trichinella zimbabwensis	KRZ15665	-----T-W--YF--QN----I-IT		DLVEVR-N---A------
	Trichinella sp. T8	KRZ90261	-----T-W--YF--QN----I-IT		DLVEVR-N---A------
	Trichuris trichiura	CDW56901	PNS--T-W---T-VE---AKMDIR		DVV-VR-Y---V------
	Trichuris suis	KFD73083	-----T-W---I-VE---AKMDIR		DVV-VR-Y---V------
Outgroups (0/56)	*Acanthochromis polyacanthus*	XP_022052239	-I---T-W---E--TE-IR-I-IT		DVLEI------A------
	Acromyrmex echinatior	XP_011056593	-----T-W-S-Q-ITD-V--I-VT		D--EV------A------
	Bombus impatiens	XP_012240732	-----T-W-S-Q-ITD-V--I-VS		D-VEV------A------
	Danio rerio	XP_005164510	-I---T-W---E--TD-IR-I-IN		DVLEI------A------
	Melipona quadrifasciata	KOX77759	-----T-W-S-Q-ITD-V--I-VS		D-VEV------A------
	Monopterus albus	XP_020455096	-I---T-W---E--TE-IL-I-IT		DVLEI------A------
	Pelecanus crispus	XP_009492064	-I---T-W---E--TE-VH-L-VN		DILEI------A------
	Rhincodon typus	XP_020384749	-I---T-W---E--TE--H-M-IT		DLLEI------A------
	Homo sapiens	3P5T_L	-I---T-W---E--TE-VH-L-VN		DILEI------A------
	Mus musculus	BAC32898	-I---T-W---K--TE-VH-L-VN		DILEI------A------

Supplementary Figure 40. Partial sequence alignments of a Cleavage Factor IM (CFIm) homolog protein with a CSI consisting of a 2 aa insertion (shown in black box) that is specific for the *Rhabditoidea* suborder. The CSI is not present in other nematodes or any non-nematode outgroup.

Group	Species	Accession	158		200
Rhabditoidea (6/6)	*Caenorhabditis elegans*	NP_001021012	KDKLDVNFIGLALHDQPLCE	RI	PIAHIVEEISSQEKRVLDARK
	Caenorhabditis remanei	XP_003114838	-----GS-MA--H-------	-V	-F-QL----------------
	Caenorhabditis brenneri	EGT39543	-----A-YMA--H----I--	-V	-F-QVGD-VAA----------
	Caenorhabditis briggsae	XP_002640351	-----AS-MA--H----I--	-V	IF-SLN---AA---------R
	Caenorhabditis nigoni	PIC52865	-----ASYMA--H----I--	-V	IF-SLN---VA---------R
	Diploscapter pachys	PAV72761	-E--AE-LLLNYDPN--MIQ	W-	DKITLE-D-RA----------
Other Nematodes (0/21)	*Ancylostoma ceylanicum*	EYC21265	-E--ES-LVLNVNPE---LR		--SVLP-D-TA--R-------
	Ancylostoma duodenale	KIH64568	-E--ES-LVLNVNPE---LR		--SVLP-D--A--R-------
	Brugia malayi	CRZ25463	-KQ-ESSPSFSTNV---FIQ		AMSVSEQD-QQ--R----M--
	Dictyocaulus viviparus	KJH53557	-E--ES-LLLYVNPE---LR		-VTVLP-D-TN--R-------
	Haemonchus contortus	CDJ84966	-E--ES-LMLNVNP---ILR		--TVQP-D-TA--R-------
	Loa loa	XP_003138092	-KQ-ES-PSVNTNV---FIQ		AMSVSEQD-QQ--R----M--
	Necator americanus	XP_013307984	-E-MES-LLLHINPE---LR		--SVLP-D-AT--R-------
	Oesophagostomum dentatum	KHJ92924	-E--ES-LLLNVNPE---LR		--SV-P-DVTA--R-------
	Onchocerca flexuosa	OZC07137	-KQ-ES-PSVNTNV---FIQ		AVSVSEQD-QQ--RL---M--
	Pristionchus pacificus	AAV85979	GKSIEH-PR-TMDT----IA		GMVVMEDDVKT--R--I----
	Teladorsagia circumcincta	PIO61978	-E--ES-LMLHVNP---ILR		-VTVQP-D--A--R-------
	Toxocara canis	KHN80387	-KQ--S-PSVNTNVE--FVQ		AVTVNEQD-QQ--R----M--
	Trichinella britovi	KRY61524	-KS--S-PGANVNP----FQ		AVVITEDDVLL-Q---I---R
	Trichinella nelsoni	KRX26031	-KS--S-PGANVNP----FQ		AVVITEDDVLL-Q---I---R
	Trichinella papuae	KRZ75498	-KS--S-PGANVNP----FQ		AVVITEDDVLL-Q---I---R
	Trichinella pseudospiralis	KRY79108	-KS--S-PGANVNP----FQ		AVVITEDDVLL-Q---I---R
	Trichinella spiralis	XP_003378007	-KS--S-PGANVNP----FQ		AVVITEDDVLL-Q---I---R
	Trichinella nativa	KRZ63363	-KS--S-PGANVNP----FQ		AVVITEDDVLL-Q---I---R
	Trichuris suis	KHJ46414	-KA-ES-PNVNFNP----IQ		AVMISE-D-IM--R------R
	Trichuris trichiura	CDW51823	-KA-ES-PNVNFNP----IQ		AVMISE-D-MM--R------R
	Wuchereria bancrofti	EJW76445	-KQ-ESSPSVSTNV---FIQ		AMSVSEQD-QQ--R----M--
Outgroups (0/21)	*Acyrthosiphon pisum*	NP_001156167	RSHY-K E-VYRIP----VQ		EVSVT--D-K---E--IA---
	Agrilus planipennis	XP_018323606	RSS--K-PGVFLDPK---VQ		AVSIQD-D-KR--E--AA--R
	Folsomia candida	XP_021967669	RTN--N-PGVYINPE---IQ		A-VVAE-D-R---E---V---
	Harpegnathos saltator	XP_011136314	-AA-EK-PGVFLNP----VQ		AVSIAD-DLKR--E--AL---
	Ooceraea biroi	XP_011328960	-TA-EK-PGVFLNP----VQ		AVSIAD-D-KR--D--AV---
	Priapulus caudatus	XP_014677085	-SS--K-PGVYLNP----MQ		ALMVSE-D-KR--TK--E---
	Trachymyrmex cornetzi	XP_018365156	-TA-EK-PGVFLNP----VQ		AVSIAD-D-RR--D--VV---
	Trichogramma pretiosum	XP_014222284	-AQ-EK-PGSFLNP----LQ		AVTITD-D-KK--EK--A--Q

Supplementary Figure 41. Partial sequence alignments of a Methyl-CpG-binding protein with a CSI consisting of a 2 aa insertion (shown in black box) that is specific for the Rhabditoidea suborder. The CSI is not present in other nematodes or any non-nematode outgroup.

Group	Species	Accession	71	CSI	105
Rhabditoidea (7/7)	*Caenorhabditis elegans*	NP_001024660	MLVAPIGYSIRVRALQFDV	AS	TENARTCEKDTLHV
	Caenorhabditis brenneri	EGT30233	--------N----------	--	-----N-------I
	Caenorhabditis latens	OZG25193	--------------IH---	--	-----N--------
	Caenorhabditis remanei	OZG08426	--------------IH---	--	-----N--------
	Caenorhabditis nigoni	PIC18220	--------N-----IE---	--	-----N--R-----
	Caenorhabditis briggsae	CAP33035	--------N-----IE---	-R	-----N--R-----
	Diploscapter pachys	PAV79335	--I--V--R--L--IE---	-G	SGGKGS-H------
Other Nematodes (0/29)	*Ancylostoma ceylanicum*	EYB97856	--I-----R--L-V-E---		NGQNSV--------
	Ancylostoma duodenale	KIH43381	--I-----R--L-V-E---		NGQNSV-------
	Brugia malayi	XP_001899277	LIT--S--R--L-V-D-N-		LGD-HN-D------
	Dictyocaulus viviparus	KJH41256	--I-----R--LKV-D-E-		NGKNSL--------
	Haemonchus contortus	CDJ85812	--I--L--R--LKIIE---		NG-NSS--------
	Loa loa	XP_020303780	LIT--S--R--L-V-D-N-		LGD-HN-D------
	Necator americanus	XP_013293602	--I-----R--L-V-E---		NGQNSI--------
	Oesophagostomum dentatum	KHJ99798	--I-----R--L-VME---		NGQKTV--------
	Onchocerca flexuosa	OZC09977	LIM--S--R--L-V-D-N-		LG--HN-D------
	Teladorsagia circumcincta	PIO70590	--I--L--R--LKV-E---		NG-NSS--------
	Toxocara canis	KHN87003	LIT--V--R--L-V-D-N-		LGDPQN-D------
	Wuchereria bancrofti	EJW83984	LIT--S--R--L-V-D-N-		LGD-HN-D------
	Teladorsagia circumcincta	PIO70590	--I--L--R--LKV-E---		NG-NSS--------
	Heligmosomoides polygyrus	VDP41706	--I-----RV---VVE---		NGRNTS--------
	Cylicostephanus goldi	VDK45323	--------R--L-V-E---		NGQSSN--------
	Ancylostoma caninum	RCN52527	--I-----R--L-V-E---		NGQNSV--------
	Strongylus vulgaris	VDM79066	--I-----R--L-V-E---		NGQNSN--------
	Nippostrongylus brasiliensis	VDL77150	--------R--L-VIE---		NGQNSS--------
	Haemonchus placei	VDO80095	--I--L--R--LKIIE---		NG-NSS--------
	Angiostrongylus costaricensi	VDM52576	--I-----R--LKV-D---		NGKHTV--------
	Onchocerca ochengi	VDK65250	LIT--S--R--L-V-D-N-		LG--HN-D------
	Dracunculus medinensis	VDN58727	LIT-----R--L-V-D-N-		LGDPKN-N------
	Gongylonema pulchrum	VDK29616	LIT--V--R--L-V-D---		LGDSHN-D-----
	Anisakis simplex	VDK46880	LIT--V--R--L-V-D-N-		LGDPQN-D------
	Litomosoides sigmodontis	VDK71987	LIA--S--R--L-V-D-N-		LGD-HN-D------
	Thelazia callipaeda	VDN01074	LIT--A--R--L-V-D-N-		LGD-HN-D------
	Acanthocheilonema viteae	VBB30517	LIT--S--R--L-V-D-N-		LGD-HN-D------
	Brugia timori	VDO32583	LIT--S--R--L-V-D-N-		LGD-HN-D------
	Brugia pahangi	VDN90800	LIT--S--R--L-V-D-N-		LGD-HN-D------
Outgroups (0/>100)	*Drosophila melanogaster*	NP_476879	TIA--DNSYVQLIF-T--I		-SSEN-TF-YVQ-
	Chrysemys picta bellii	XP_005307507	L--SER--RVELTFQT-E-		-EEAD-GY-YIEL
	Eurypyga helias	XP_010159303	VI--ED--GVELIFQT-EI		-EEAD-GY-YME-
	Gekko japonicus	XP_015279232	L----E-HT-NLTFVA-E-		-RHSS-RW-SVTI
	Papilio xuthus	XP_013164435	SI---M-HFVKLTF-T-EL		-PEVN-GY-FVQ-
	Limulus polyphemus	XP_022247735	IIE-ED-V-V-L-FMT-H-		-HEQD-GY-YVEI
	Heterocephalus glaber	XP_004839034	VI--ED--GVEL-FQT-E-		-EEAD-GY-YMEA
	Exaiptasia pallida	XP_020911194	KIASRS-- -KL-FKE--L		--EKF-SY-EVII
	Homo sapiens	XP_006527155	QV---VQ-R-SLQFEA-EL		-GNDV-KY-FVE-
	Mus musculus	XP_017173690	QV---VQ-R-SLQFEA-EL		-GNDV-KY-FVE-
	Drosophila biarmipes	XP_016960768	TIA--DNSYVQLIF-T--I		-SSEN-TF-YVQ-
	Musca domestica	XP_005182121	TIQ--PNSYVNLIF-T--L		-SSEN-TY-YVQ-
	Stomoxys calcitrans	XP_013103389	TIQ-SPNSYVQLIF-T--L		-SSEN-TY-YVQ-
	Ceratitis capitata	XP_004523366	TIQ--ASSNVQLLF-T--I		-SSEN-TY-YVQ-
	Zeugodacus cucurbitae	XP_011186021	TIQ--ASSNVQLLF-T--I		-ASEN-TY-YVQ-
	Sarcophaga bullata	TMW45646	TIQ--PSSNVQLIF-T--L		--SEN-TY-YVQ-
	Vanessa tameamea	XP_026492763	SI---V-QFV-LTF-T--L		-PEDN-GY-YVQ-
	Amyelois transitella	XP_013200300	SI---P-QFVKLTF-T-EL		-PEAN-GY-YVQ-
	Chilo suppressalis	RVE47431	SI-----QFV-LTF-T-EL		-PEAN-GY-YVQ-
	Ostrinia furnacalis	XP_028170358	SI---M-QFV-LTF-T-EL		-PEAN-GY-YVQ-
	Eumeta japonica	GBP18868	SI---L-HFV-LTF-T-EL		-PEPN-RY-YVQ-

Supplementary Figure 42. Partial sequence alignments of an Abnormal cell migration protein 13 with a CSI consisting of a 1 aa insertion (shown in black box) that is specific for the Rhabditoidea suborder. The CSI is not present in other nematodes or any non-nematode outgroups.

Group	Species	Accession	126		164
Rhabditoidea (6/6)	*Caenorhabditis elegans*	NP_001250840	KEEWDPMSLRQTRHLTTFCSLF		EKLPNLTEKSKQFNAFL
	Caenorhabditis remanei	OZF79963	-------------N-A----I-		-----------------
	Caenorhabditis latens	OZG18420	------------KN-A----I-		------N----------
	Caenorhabditis briggsae	CAP31469	-------------N-A-C--I-		---------------L-
	Caenorhabditis brenneri	EGT32791	-D----------KK-AM---I-		--I----D-----TG--
	Diploscapter pachys	PAV85955	TT----L--S--NR-AQILNDL		HVY-T--R--RP-LKL-
Other Nematodes (0/30)	*Pristionchus pacificus*	PDM66686	SD--E-V--T-STRIS-LIRTL	L	QDT-S-DQS--PLKRL-
	Ancylostoma ceylanicum	EYB89229	NV--E----K-SQR-GAVL-GL	S	DYPKLNV-D--S-AVLA
	Ancylostoma duodenale	KIH55911	NV--E----K-SQR-GAVL-GL	S	DYPKLNV-D--S-AVLA
	Ascaris suum	ERG87385	Q-Q---L-QK-CSR-GFLL-SL	V	DEC-T-VPS-RSVKRL-
	Brugia malayi	XP_001893893	R-R---L-QH-C-N-GFLLNQL	I	DEC-T-VPS-RPVQKL-
	Dictyocaulus viviparus	KJH47162	NV--E----K-SKR-GAVLCGL	S	DYPELNI-D--S-A-LA
	Haemonchus contortus	CDJ93966	NV--E----K-SKR-GAVL-GL	S	DYPKMNIDD--S-AILA
	Necator americanus	XP_013298932	NV--E----K-SQR-GAVL-GL	S	DYPKLNVDE--S-AVLA
	Oesophagostomum dentatum	KHJ88487	NV--E----K-SKR-GAVL-GL	T	DYPKMNV-D--A-A-LA
	Toxocara canis	KHN84480	Q-Q---L-QK-CSR-GFLLDSL	V	DEC---VPS-RSVKRL-
	Wuchereria bancrofti	EJW84293	R-R---L-QQ-C-N-GFLLDQL	I	DEC-T-VPS-RPVQKL-
	Enterobius vermicularis	VDD94889	-R---L-EK-CKR-GFLLESL	V	-EC-SMVSS-QPVQRL-
	Brugia pahangi	VDN84991	-R---L-QH-C-N-GFLLNQL	I	DEC-T-VPS-RPVQKL-
	Brugia timori	VDO28846	---L-QH-C-N-GFLLNQL	I	DEC-T-VPS-RPVQKL-
	Onchocerca flexuosa	VDO64826	-R---L-QH-CQN-GFLLNQL	I	DEC-T-VPT-RPVQKL-
	Onchocerca ochengi	VDK64967	---L-QH-C-N-GFLLNQL	I	DEC-T-IST-RPVQKL-
	Litomosoides sigmodontis	VDK71787	---L-QH-C-N-GFLL-QL	I	DEC-T-VPS-RPVQKL-
	Acanthocheilonema viteae	VBB26053	-R---L-QH-C-N-GFLL-QL	I	DEC-T-VPS-RPVQKL-
	Gongylonema pulchrum	VDN36303	-R---L-Q-HC-N-ALLLNQL	I	DEC-T-MVS-RPVQKL-
	Loa loa	XP_020302321	---L-QH-C-N-GFLLNQL	I	DEC-T-VPN-RPVQKL-
	Trichinella britovi	KRY50355	NKV---T-GK---R-VD-LDNL	F	TNY-T--PE-G-VGSLV
	Trichinella murrelli	KRX42937	NKV---T-GK---R-VD-LDNL	F	TNY-T--PE-G-VGSLV
	Trichinella nativa	KRZ54145	NKV---T-GK---R-VD-LDNL	F	TNY-T--PE-G-VGSLV
	Trichinella nelsoni	KRX20309	NKV---T-GK---R-VD-LDNL	F	TNY-T--PE-G-VGSLV
	Trichinella papuae	KRZ76140	NKV---T-GK---R-VD-LDNL	F	TNY-T--PQ-E-VC-LV
	Trichinella pseudospiralis	KRZ30430	NKV---T-GK---R-VH-LDNL	F	TNY-T--PQ-E-VC-LV
	Trichinella spiralis	XP_003379916	NKV---T-GK---R-VD-LDNL	F	TNY-T--PE-G-VGSLV
	Trichinella zimbabwensis	KRZ05150	NKV---T-GK---R-VH-LDNL	F	TNY-T--PQ-E-VC-LV
	Trichuris suis	KHJ43583	-KV-R-A-SS--EL-CS-LRHL	F	STY-T--GE--RTTQL-
	Trichuris trichiura	CDW52567	-KV-R-A-SS--EL-C--LRHL	F	STY-T--GE--TTRQL-
Outgroups (0/>100)	*Acanthaster planci*	XP_022100458	--V---V-ST--NR-VSLIHKL	V	-DY-TVNSD--NTRDL-
	Acromyrmex echinatior	XP_011056917	EKI-----TS--LR-VGTINRL	I	KEY---NDS---LETLF
	Aedes aegypti	XP_021709601	EQV---L-TT--LK-VRLINRL	S	RDY-S-RRTC--LRLLF
	Microtus ochrogaster	XP_005364761	EFI---L-AS---S--VH-R-V	F	-HSASEN-V--SKQDL-
	Rattus norvegicus	XP_006236765	EMI---L-TS---I--VH-RVA	F	-QFASE--V--SKQDL-
	Strongylocentrotus purpuratus	XP_011664408	E-V-----TL--HR-IDTLHQL	A	QDY-TISADN-NTQLL-
	Drosophila ananassae	XP_001953199	T-S---L-TT--LR-VG-INRL	G	REF- -SGTN--L-KLF
	Homo sapiens	NP_057715	ENM---F-TT--SRMVGITLKL	I	NGY-SVVNAEN-NTQVY
	Mus musculus	XP_011239693	ETI---L-TS---S--VH-RVA	F	-QFASEN-V- -NKQDL

Supplementary Figure 43. Partial sequence alignments of a PAX3- and PAX7 binding protein 1 protein with a CSI consisting of a 1 aa in (shown in black box) that is specific for the Rhabditoidea suborder. The CSI is not present in other nematodes or any non-nematode outgroup.

Group	Species	Accession	234		270
Chromadorea (28/30)	*Oesophagostomum dentatum*	KHJ83757	LLTINETTCEQAKPAVLKFDF		KADYNMGKMNNFKQVF
	Pristionchus pacificus	PDM73700	-ISL---------IPNIRG-P		--N-DV-RWK-IEI--
	Ancylostoma ceylanicum	EYC21828	--S------------------		-----L---K--Q---
	Haemonchus contortus	CDJ92987	--S------------------		-----L-REK--E---
	Dictyocaulus viviparus	KJH51495	-ISL----------S------		-----L--EK--L---
	Diploscapter pachys	PAV75305	-VEL-----------I-R--N		M------RAA-L-KI-
	Caenorhabditis brenneri	EGT55552	-ISL----V--T-----R--N		A-------W---RA--
	Caenorhabditis remanei	XP_003101477	-IAL----V--T-----R--N		A-------W---RS--
	Caenorhabditis elegans	NP_001021339	-ISL----V--T---L-R--N		A-------Y---QS--
	Caenorhabditis briggsae	CAP28989	-ISL----V--T-----R--N		A-------W---RA--
	Caenorhabditis latens	OZG13909	-IAL----V--T-----R--N		A-------W---RS--
	Caenorhabditis nigoni	PIC27080	-ISL----V--T-----R--N		A-------W---RA--
	Ascaris suum	ERG81007	-ASL----------PNIRG--		-------TYR-LRAA-
	Toxocara canis	KHN74907	-ASL----------PNIRG-V		-------TYR-LRAA-
	Brugia malayi	CDQ04118	-A-L----------PNIRG-S		N------IYR-LRA--
	Loa loa	XP_003144956	-A-L----------PNIRG-S		N------TYR-LRA--
	Onchocerca flexuosa	OZC08312	-AAL----------PNIRG-S		N------TYR-LRA--
	Litomosoides sigmodontis	VDK69424	-A-L----------PNIRG-S		S----V-TYR-LRA--
	Ancylostoma caninum	RCN31337	--S------------------		-----L---K--Q---
	Cylicostephanus goldi	VDK84008	--S------------N-----		-------RR---Q---
	Haemonchus placei	VDO41434	--S------------------		-----L-REK--E---
	Heligmosomoides polygyrus	VDO94671	--S------------------		-----L-REK--Q---
	Angiostrongylus costaricensis	VDM53442	-ISV----------S------		-----L-RAK--M---
	Brugia pahangi	VDN86328	-A-L----------PNIRG-S		N------IYR-LRA--
	Onchocerca ochengi	VDM94527	-A-L----------PNIRG-S		N------TYR-LRA--
	Acanthocheilonema viteae	VBB27020	-V-L----------PNIRG-S		N----V-TYR-LRA--
	Anisakis simplex	VDK66445	-ASL----------PNIRG-P		-------TYR-LRAA-
	Thelazia callipaeda	VDN01689	-ASL----------PNIRG-S		N------AYR-LRA--
	Strongyloides ratti	CEF66832	-IK--------C--PIFRGGN	I	N-T--TTVKE-IRSAL
	Wuchereria bancrofti	EJW86797	-TAR-QS-I-SFR-P-FIYGI	D	-NGF-L-IRR--R---
Outgroups (0/62)	*Amyelois transitella*	XP_013186177	-VMN-R--L-AFRAPMFRGGA	D	-NGFSL-AY----E--
	Amborella trichopoda	XP_020522962	-IST-Q--Y-NFR YRY-R	R	ANP--L-VV----EI-
	Hydra vulgaris	XP_012562211	--SK-R--L-SFRAPLFHYGA	D	-DGF-I-T---IR---
	Theileria equi	XP_004830431	-IS--K--I-FCE WKAS	G	SYN--L-IIS------
	Zea mays	ONM56237	-MST-Q--Y-NFR YRY -R	R	ANP--I-IL---MEI-
	Fonticula alba	XP_009496369	-IAR-R--FD FSYFYHIYKE	L	YDQ----PRG--A-I-
	Papilio xuthus	XP_013166240	-VAH-R--L-AFRAPMFRGGA	D	-NGFSL-AF----E--
	Opisthorchis viverrini	XP_009163389	-VMK-KL-L-FHR-PIFRDAS	R	RDSFDL-VKQ-MA---

Supplementary Figure 44. Partial sequence alignments of a Palmitoyltransferase protein with a CSI consisting of a 1 aa deletion (shown in black box) that is specific for the Chromadorea class. The CSI is not present in any non-nematode outgroup. The CSI is not present in the species *Strongylodies ratti* and *Wuchereria bancrofti*. Blastp search shows no homologs of this protein present in species of the Enoplea class.

Group	Species	Accession	255		282
Chromadorea (20/20)	*Oesophagostomum dentatum*	KHJ83757	KADYNMGKMNNFKQVFG		WGLWLFPLITS
	Ancylostoma ceylanicum	EYC21828	-----L---K--Q----		---------N--
	Haemonchus contortus	CDJ92987	-----L-REK--E----		---------K--
	Dictyocaulus viviparus	KJH51495	-----L--EK--L----		---------R-T
	Diploscapter pachys	PAV75305	M------RAA-L-KI--		-----V--P-D
	Caenorhabditis brenneri	EGT55552	A-------W---RA---		----MW-IESN
	Caenorhabditis remanei	XP_003101477	A-------W---RS---		----MW-IESN
	Caenorhabditis elegans	NP_001021339	A-------Y---QS---		-----C-IDS-
	Caenorhabditis briggsae	CAP28989	A-------W---RA---		--V-MW-IESN
	Caenorhabditis latens	OZG13909	A-------W---RS---		----MW-IESN
	Ascaris suum	ERG81007	-------TYR-LRAA--		-------VS-H
	Toxocara canis	KHN74907	-------TYR-LRAA--		-------VS-H
	Brugia malayi	CDQ04118	N------IYR-LRA---		----A--VDSH
	Loa loa	XP_003144956	N------TYR-LRA---		----A--V
	Onchocerca flexuosa	OZC08312	N------TYR-LRA---		----A--VDSH
	Pristionchus pacificus	PDM73700	--N-DV-RWK-IEI---		-------VR-T
	Strongyloides ratti	CEF66832	N-T--TTVKE-IRSAL-		-------VD-E
	Wuchereria bancrofti	EJW75594	--IYR-LRA---		----A--VDSH
Other Nematodes (0/12)	*Trichuris suis*	KFD53008	-NGF-Y-TLR--QE---	KR	LL--FL-VY--
	Trichuris trichiura	CDW56012	-NGF-Y-TLR--QE---	KR	PL--FL-VY-
	Trichinella nelsoni	KRX27745	-EGF-L-TTR--REI--	DS	PFY--I-VFS-
	Trichinella spiralis	KRY42951	-EGF-L-TTR--REI--	DS	PFY--I-VFS-
	Trichinella patagoniensis	KRY17610	-EGF-L-TTR--REI--	DS	PFY--I-VFS-
	Trichinella sp. T9	KRX64942	-EGF-L-TTR--REI--	DS	PFY--I-VFS-
	Trichinella murrelli	KRX48841	-EGF-L-TTR--REI--	DS	PFY--I-VFS-
	Trichinella nativa	KRZ58482	-EGF-L-TTR--REI--	DS	PFY--I-VFS-
	Trichinella papuae	KRZ81046	-EGF-L-MSR--REI--	DS	PFY--I-VFS-
	Trichinella pseudospiralis	KRY00051	-EGF-L-MSR--REI--	DS	PFY--I-VFS-
	Trichinella zimbabwensis	KRZ19084	-EGF-L-MSR--REI--	DS	PFY--I-VFS-
	Soboliphyme baturini	VDP43282	-DGF-L-VRK--VE---	KN	KVT-IL-IF--
Outgroups (0/62)	*Aedes aegypti*	XP_021697678	-NGFSL--L---QE---	DD	RK--FV-VY--
	Hydra vulgaris	XP_012562211	-DGF-I-T---IR----	NT	TWK-F--IF-T
	Papilio xuthus	XP_013166236	-NGFSL-AF----E---	NS	PN--ML-VF--
	Sarcophilus harrisii	XP_012408785	-NGF-L-FIR--Q----	EN	KK---L-IGS-
	Gossypium raimondii	XP_012480671	YDLGRKK --E----	TK	KS------FS
	Drosophila melanogaster	NP_724868.2	-NG--L-RYA--CE---	DD	-QY-FL-VFS-
	Danio rerio	XP_021331799	SKAFDV-VQA--L----	KK	KR-----VFS-
	Stomoxys calcitrans	XP_013106168	-NGF-L--FA--QE---	DN	-K--FL-IYS-

Supplementary Figure 45. Partial sequence alignments of a Palmitoyltransferase protein with a CSI consisting of a 2 aa deletion (shown in black box) that is specific for the Chromadorea class. The CSI is not present in any non-nematode outgroup.

Group	Species	Accession	162		194
Chromadorea (33/33)	*Caenorhabditis brenneri*	EGT30700	AGLLGSFSYAFLTE	PHM	ANLTPKVALLIQLFIP
	Caenorhabditis briggsae	CAP22728	--------------	---	----------------
	Caenorhabditis elegans	NP_505237	--------------	---	----------------
	Caenorhabditis latens	OZF90828	--------------	---	----------------
	Caenorhabditis nigoni	PIC22854	--------------	---	----------------
	Caenorhabditis remanei	OZF85401	--------------	---	----------------
	Ancylostoma ceylanicum	EYC10320	-------------D	RS-	-D-Q------------
	Ancylostoma duodenale	KIH62876	-------------D	RS-	-D-Q------------
	Brugia malayi	XP_001901223	--II--LT--A---	--F	L--S-RIT---M-VV-
	Dictyocaulus viviparus	KJH42909	------LA------	KS-	------D-V-L-----
	Diploscapter pachys	PAV60708	---I------L---	-K-	G--S--TT--------
	Necator americanus	XP_013296815	--VV-A----G--D	RQL	LA-S-TE-M-VM-VV-
	Strongyloides ratti	CEF68195	---V---A--G---	-NW	---S--N-C-VMQI--
	Haemonchus contortus	CDJ95123	---I----------	RT-	G--Q--D---------
	Loa loa	XP_020304569	--IF---A--I--D	M--	LG---QN---LM-IV-
	Oesophagostomum dentatum	KHJ93977	-------------D	RS-	G--Q----------V-
	Onchocerca flexuosa	OZC12673	--II-ALA--V---	--F	L--S--TT---M-IV-
	Pristionchus pacificus	PDM69720	--VF-ALA--S--D	AA-	------Q---TM-VV-
	Teladorsagia circumcincta	PIO76278	---I------L---	-S-	G--Q------------
	Toxocara canis	KHN85722	--V--A-T--A--D	S--	LAMS-RT---TM----
	Wuchereria bancrofti	EJW86032	--II--LT--A---	--F	L--S-RIT---M-VV-
	Nippostrongylus brasiliensis	VDL62951	--------------	KS-	G--S------------
	Ancylostoma caninum	RCN29588	-------------D	RS-	-D-Q------------
	Heligmosomoides polygyrus	VDO71568	---I---------D	-AI	G--Q----------V-
	Haemonchus placei	VDO66999	---I----------	RT-	G--Q--D---------
	Brugia timori	VDO29491	--II--LT--A---	--F	L--S-RIT---M-VV-
	Brugia pahangi	VDN82008	--II--LT--A---	--F	L--S-RIT---M-VV-
	Acanthocheilonema viteae	VBB29710	--IF---A--M--D	M--	LG---QN---LM-IV-
	Enterobius vermicularis	VDD87126	-SIF--LI--G---	--F	-H-S--N---LM-C--
	Litomosoides sigmodontis	VDK87641	--IF---A--I--D	MS-	LG---QK---VM-IV-
	Strongylus vulgaris	VDM81356	---V------G--D	RNL	LG---AQ-M-VM-VV-
	Angiostrongylus costaricensis	VDM52847	---A-T----VM-D	VNL	LA---TD-M-VM-VV-
	Thelazia callipaeda	VDN08382	--IF---T--L--D	KR-	LG-L-QNT--TM-I--
Outgroups (0/>200)	*Acanthaster planci*	XP_022105346	S--VS-LT--G-A-		V-G-EKS--VMVVV-
	Acinonyx jubatus	XP_014917735	-----AL--LG--Q		-G-S-QHT--SM-G--
	Acromyrmex echinatior	XP_011053297	--VI-AV---G--L		V-SIENT--VM-IV-
	Acropora digitifera	XP_015763073	---F-AL---G--S		-GMS-RK-V-VMNV--
	Homo sapiens	AAD01558	-----AL--LG--Q		-G-S-QQT--SM-G--
	Mus musculus	AAH47120	------L--LG--Q		-G-S-QHT--SM-G--
	Callithrix jacchus	XP_017834325	-----AL--LG--Q		-G-S-QHT--SM-G--
	Thamnophis sirtalis	XP_013931658	---I-AL--LG--Q		IG-S-QDT--FMTIV-
	Octopus bimaculoides	XP_014786536	--IF-AL---G--M		L--S-RHTI-VM-I--

Supplementary Figure 46. Partial sequence alignments of a Battenin protein with a CSI consisting of a 3 aa insertion (shown in black box) that is specific for the Chromadorea class. The CSI is not present in any other nematode species or non-nematode outgroup.

Group	Species	Accession	122	CSI	155
Chromadorea homolog 1 (33/33)	*Dictyocaulus viviparus*	KJH47557	GQIQLWQFLLELLADA	A	SNAHCIAWEGSSGEFKL
	Haemonchus contortus	CDJ98481	----------------	-	----------A------
	Ancylostoma ceylanicum	EYC19665	----------------	-	----------G------
	Ancylostoma duodenale	KIH51654	----------------	-	----------G------
	Necator americanus	XP_013309383	----------------	-	----------G------
	Oesophagostomum dentatum	KHJ79766	----------------	-	----------G------
	Haemonchus placei	VDO42951	----------------	-	----------A------
	Heligmosomoides polygyrus	VDO88533	----------------	-	----------G------
	Angiostrongylus costaricensis	VDM63452	-------------G--	-	--G-------A------
	Brugia malayi	CRZ25787	-------------S-T	H	------T---QN-----
	Diploscapter pachys	PAV55673	--------------EG	S	V-S---V---GI-----
	Loa loa	XP_020301541	-------------S-T	H	------T---QN-----
	Onchocerca flexuosa	OZC06734	-------------S-T	H	------T---QN-----
	Teladorsagia circumcincta	PIO60565	----------------	G	----------G------
	Toxocara canis	KHN83764	-------------S--	H	----------GN-----
	Wuchereria bancrofti	EJW86656	-------------S-T	H	------T---QN-----
	Acanthocheilonema viteae	VBB28050	---------------T	H	------T---QN-----
	Thelazia callipaeda	VDM95915	-------------S-T	H	------T---QN-----
	Gongylonema pulchrum	VDK27269	-------------S-T	H	------T---QN-----
	Litomosoides sigmodontis	VDK82646	-------------S-T	H	------T---EN-----
	Brugia pahangi	VDN92405	-------------S-T	H	------T---QN-----
	Brugia timori	VDO08037	-------------S-T	H	------T---QN-----
	Onchocerca ochengi	VDK80025	-------------S-T	H	------T---QN-----
	Anisakis simplex	VDK48258	-------------S--	H	T--Y------AN-----
	Dracun culus medinensis	VDN58716	-------------S-S	Q	NY---------G-----
	Caenorhabditis latens	OZG24979	-TVH----IR---DQP	K	QYGT-VR-*RDE-T--I *VE
	Caenorhabditis remanei	OZG07314	-TVH----IR---DQP	K	QYGT-VR-*RDE-T--I *VE
	Caenorhabditis nigoni	PIC18783	-TVH----IR---DQP	K	QYSN-VR-*RDE-T--I *VD
	Caenorhabditis briggsae	XP_002644898	-TVH----IR---DQP	K	QYSN-VR-*RDE-T--I *VD
	Caenorhabditis elegans	NP_001123137	-TVH----IR---DQP	K	QYSA-VR-*RDE-T--I *VD
	Caenorhabditis brenneri	EGT43113	-TVH----IR---DQP	K	QYSA-VR-*RDE-T--I *VD
	Strongyloides ratti	XP_024499015	--------I----GND	H	IFSY--S---GH-----
	Enterobius vermicularis	VDD96228	-TVH--H-IR---DQP	K	QYSS-VR-*REE-T--I *VD
Chromadorea homolog 2 (0/10)	*Strongyloides ratti*	CEF71270	-------------S-S		R--DS-T---TQ-----
	Nippostrongylus brasiliensis	VDL85697	--T------------K		RYDDV-T---TQ-----
	Enterobius vermicularis	VDD92848	-------------S-S		C--DV-T---T------
	Caenorhabditis brenneri	EGT30352	----------------		V----------N-----
	Caenorhabditis elegans	NP_001263956	----------------		V----------N-----
	Caenorhabditis latens	OZG25316	----------------		V----------N-----
	Caenorhabditis remanei	OZG07579	----------------		V----------N-----
	Caenorhabditis nigoni	PIC18295	----------------		V-ST-------N-----
	Caenorhabditis briggsae	XP_002644635	----------------		V-ST--T----N-----
Enoplea (0/14)	*Trichinella nativa*	OUC48995	-------------S-G		R-ME--T---TN-----
	Trichinella sp. T6	KRX74809	-------------S-G		R-ME--T---TN-----
	Trichinella patagoniensis	KRY11413	-------------S-G		R-ME--T---TN-----
	Trichinella nelsoni	KRX14286	-------------S-G		R-ME--T---TN-----
	Trichinella pseudospiralis	KRX92484	-------------S-G		R-ME--T---TN-----
	Trichinella zimbabwensis	KRZ13603	-------------S-G		R-ME--T---TN-----
	Trichinella papuae	KRZ73610	-------------S-G		R-ME--T---TN-----
	Trichinella spiralis	XP_003368363	-------------S-N		---AF-T---IN-----
	Trichinella britovi	KRY57928	-------------S-N		---AF-T---IN-----
	Trichinella murrelli	KRX47624	-------------S-N		---AF-T---IN-----
	Trichuris suis	KFD53731	-------------S-G		R-ME--T---TN-----
	Trichuris trichiura	CDW56634	-------------S-N		A--SF-----MN-----
Outgroups (0/>200)	*Soboliphyme baturini*	VDO94732	-------------S-S		A-SAF-----TN-----
	Acanthisitta chloris	XP_009078900	-------------S-S		---S--T---TN----M
	Aedes aegypti	XP_021712753	-------------S-S		A--A--T---TN-----
	Aethina tumida	XP_019876307	-------------S-S		---A--T---TN-----
	Alligator mississippiensis	KYO39391	-------------S-R		A-L-------AN-----
	Camelus bactrianus	XP_010947219	---------------R		A--G------GH-----
	Octopus bimaculoides	KOF74414	-------------S-T		G-SN--T---TN-----
	Sorex araneus	XP_004617195	---------------R		A--G------GH-----
	Mus musculus	BAC39053	-------------S-S		A--S--T---TN----M
	Homo sapiens	NP_001257941	-------------S-S		A--S--T---TN----M

Supplementary Figure 47. Partial sequence alignments of an ETS (E26 transformation-specific) class transcription factor protein with a CSI consisting of a 1 aa insertion (shown in black box) that is specific for the Chromadorea class. There are two homologs of this protein in the Chromadorea class, one of which contains the CSI. The CSI is not present in species of the Enoplea class and in any non-nematode outgroup.

Group	Species	Accession	93	CSI	122
Chromadorea homolog 1 (34/34)	*Dictyocaulus viviparus*	KJH46562	HAPGCKLLLSGI	N	WDYHSVDTLRIYFEKFG
	Oesophagostomum dentatum	KHJ99864	Q----------L	-	----R-----V------
	Caenorhabditis elegans	NP_741785	P--------N--	T	NGV----S--V---T--
	Caenorhabditis brenneri	EGT31853	PL--Y-------	V	MRV-CI-F-LR--NTY-
	Caenorhabditis briggsae	XP_002644659	PS----------	T	SGV----S--V---T--
	Caenorhabditis latens	OZG25695	P-----------	T	GGV----S--V---T--
	Caenorhabditis remanei	XP_003117544	P-----------	T	SGI----S--V---T--
	Ancylostoma ceylanicum	EPB71169	Q----------L	-	----R------------
	Ancylostoma duodenale	KIH66985	Q----------L	-	----R------------
	Haemonchus contortus	CDJ83341	QS---------L	-	-----------------
	Necator americanus	XP_013302959	Q----------L	-	----R------------
	Angiostrongylus costaricensis	VDM52269	------------	-	----T------------
	Nippostrongylus brasiliensis	VDL71477	Q----------L	-	-----------------
	Strongylus vulgaris	VDM74679	Q----------L	-	----R-----V------
	Ancylostoma caninum	RCN42914	Q----------L	-	----R------------
	Diploscapter pachys	PAV85317	P----------L	H	FE-----CI-L------
	Pristionchus pacificus	PDM83796	IPQ----EVT--	S	C-F-T-ELM-KH-----
	Ascaris suum	ERG84717	PD-----S----	H	-E--T--V--H--DT--
	Brugia malayi	XP_001897671	PD-----S----	H	-AW-T--D--Q--DN--
	Loa loa	XP_020306622	PD-----S----	H	-AW-T--D--Q--DN--
	Onchocerca flexuosa	OZC10654	PD-----S----	H	-AW-T--D--Q--DN--
	Strongyloides ratti	CEF60414	PST---ITI---	H	--F-T--GI-T--DR--
	Toxocara canis	KHN76068	PD-----S----	H	----T--G--H--D---
	Enterobius vermicularis	VDD93996	-----S----	H	--F-T--G--E--DS--
	Anisakis simplex	VDK45885	-----S----	H	--F-T--E--H--DT--
	Litomosoides sigmodontis	VDK85343	S-----S----	H	-AW-T--D--Q--D---
	Brugia pahangi	VDN92749	-----S----	H	-AW-T--D--Q--DN--
	Onchocerca ochengi	VDK66688	-----S----	H	-AW-T--D--Q--DN--
	Acanthocheilonema viteae	VBB25398	-----S----	H	-AW-T--D--Q--DN--
	Caenorhabditis nigoni	PIC18338	S-------N--	T	SGV----S--V---T--
	Gongylonema pulchrum	VDN34668	-----S----	H	-AW-T--D--Q--DS--
	Thelazia callipaeda	VDN00755	-----S----	H	-AR-T--D--Q--DN--
	Dracunculus medinensis	VDN56236	-----F----	Y	SSK----I--R--A---
	Wuchereria bancrofti	EJW79684	QIFV---	H	-AW-T--D--Q--DN--
Chromadorea homolog 2 (0/12)	*Caenorhabditis brenneri*	EGT50557	NVSTKR-YV--V		RED-TE-M-TE--S-Y-
	Caenorhabditis briggsae	CAP25844	NVSTKR-YV--V		RED-TEEM-SD--S-Y-
	Caenorhabditis elegans	NP_500326	NVSTKR-YV--V		RED-TE-M-TE--T-Y-
	Caenorhabditis latens	OZG20332	NVSTKR-YV--V		RED-NE-M-TD--S-Y-
	Caenorhabditis remanei	XP_003094843	NVSTKR-YV--V		RED-NE-M-TD--S-Y-
	Dictyocaulus viviparus	KJH52650	NVSTKR-YV--V		RED-TEEMFTE--S---
	Loa loa	XP_020307328	NVSSKR-YV--V		REE-TEQMFED--SQ--
	Oesophagostomum dentatum	KHJ92511	NVSTKR-YV--V		RED-TEEMFTE--S---
	Onchocerca flexuosa	OZC08989	NVSSKR-YV--V		REE-TEQMFED--NQ--
	Pristionchus pacificus	PDM78546	NVSSKR-YV---		REE-TDEQ-QD--A---
	Wuchereria bancrofti	EJW79347	NISSKR-YV--V		REE-TE-MFKEH-G-Y-
	Toxocara canis	KHN82967	NISSKR-YV--V		RED-TE-MFKDH-G-Y-
Other Nematodes (0/11)	*Trichinella britovi*	KRY59221	-ISVK-VYI---		K-E-TE-M--E--MQY-
	Trichinella murrelli	KRX44628	-ISVK-VYI---		K-E-TE-M--E--MQY-
	Trichinella nativa	KRZ57303	-ISVK-VYI---		K-E-TE-M--E--MQY-
	Trichinella nelsoni	KRX20916	-ISVK-VYI---		K-E-TE-M--E--MQY-
	Trichinella papuae	KRZ68309	-ISVK-VYI---		K-E-TE-M--E--MQY-
	Trichinella patagoniensis	KRY20454	-ISVK-VYI---		K-E-TE-M--E--MQY-
	Trichinella pseudospiralis	KRZ44360	-ISVK-VYI---		K-E-TE-M--E--MQY-
	Trichinella spiralis	XP_003381660	-ISVK-VYI---		K-E-TE-M--E--MQY-
	Trichinella zimbabwensis	KRZ13867	-ISVK-VYI---		K-E-TE-M--E--MQY-
	Trichuris suis	KHJ42825	-VSVK-VYI---		K-E-TEEM--D--S-Y-
	Trichuris trichiura	CDW53958	-VSVK-VYI---		K-E-TEEM--D--S-Y-
Outgroups (0/86)	*Acanthisitta chloris*	XP_009078363	-VTVK--FVG--		KEDTEEHH--D---EY-
	Alligator mississippiensis	XP_014458634	-VTVK--FVG--		KEDTEEHH--D---EY-
	Bos taurus	XP_015327666	LTVK--FVG--		EDTEEHH--Y----Y-
	Homo sapiens	AAH00506	-VTVK--FVG--		KEDTEEHH--D---EY-
	Hypsibius dujardini	OQV17871	Q-TV--AFVG-V		KEGVEESD--E--S---
	Mus musculus	AAI72047	HLTVK-I-VG--		KEDTKEHH--D---QY-
	Lucilia cuprina	KNC24965	NSSVK-VFVG-L		KE--DEQC-KEH-QQ--
	Xenopus laevis	P51989	-VTVK--FVG--		KEDTEEHH--E---EY-
	Zonotrichia albicollis	XP_005480939	-VTVK--FVG--		KEDTEEHH--D---EY-

Supplementary Figure 48. Partial sequence alignments of a Heterogeneous nuclear ribonucleoprotein A1 protein with a CSI consisting of a 1 aa insertion (shown in black box) that is specific for the Chromadorea class. There are two homologs of this protein in the Chromadorea class, one of which contains the CSI.

Group	Species	Accession	139		171
Chromadorea homolog 1 (19/19)	*Necator americanus*	XP_013302959	LRIYFEKFGSLDQVEILGDH		RGFGFVVFEDKQS
	Ancylostoma ceylanicum	EYB89670	--------------------		-------------
	Ascaris suum	ERG84717	--H--DT--VVE------NP		---------E-SA
	Brugia malayi	CRZ22460	--Q--DN--TVE------NP		--L------N-S-
	Caenorhabditis brenneri	EGT30022	--V---T--T--------QP		--L---IY---E-
	Caenorhabditis briggsae	CAP33098	--V---T--T--------QP		--L---IY---E-
	Caenorhabditis elegans	NP_741784	--V---T--T--------QP		--L---IY---E-
	Caenorhabditis latens	OZG25695	--V---T--T--------QP		--L---IY---E-
	Caenorhabditis remanei	OZG07648	--V---T--T--------QP		--L---IY---E-
	Dictyocaulus viviparus	KJH46562	-------------------R		-------Y-----
	Diploscapter pachys	PAV85315	I-L---------------NP		--L---I--E-E-
	Haemonchus contortus	CDJ83341	-------------------R		-------Y-----
	Loa loa	XP_020306622	--Q--DN--TVE------NP		--L------S-P-
	Oesophagostomum dentatum	KHJ99864	--V-----------------		-------------
	Onchocerca flexuosa	OZC10654	--Q--DN--TVE------NP		--L------N-S-
	Pristionchus pacificus	PDM60141	--L------T--------KP		--V------RRED
	Strongyloides ratti	CEF60414	I-T--DR--AVE------NP		--M------E-D-
	Toxocara canis	KHN76068	--H--D---VVE------NP		--L------E-TA
	Wuchereria bancrofti	EJW79684	--Q--DN--TVE------NP		--L------N-S-
Chromadorea homolog 2 (0/37)	*Caenorhabditis brenneri*	EGT29982	-HE---Q--DIEEAVVIT-R	NTQKS	--Y---TMK--A-
	Caenorhabditis briggsae	XP_002644445	-HE---Q--DIEEAVVIT-R	NTQKS	--Y---TMK--A-
	Caenorhabditis latens	OZG25472	-HE---Q--DIEEAVVIT-R	NTQKS	--Y---TMK--A-
	Caenorhabditis remanei	XP_003117654	-HE---Q--DIEEAVVIT-R	NTQKS	--Y---TMK--A-
	Caenorhabditis nigoni	PIC17906	-HE---Q--DIEEAVVIT-R	NTQKS	--Y---TMK--A-
	Caenorhabditis elegans	NP_497799	MKQ---TY-KVEDAMLMF-K	ATQRH	------T-DSDEV
	Strongylus vulgaris	1529521408	-HE---Q--DIEEAVVIT-R	QTQKS	--Y---TMK-RAA
	Ancylostoma duodenale	748398207	-HE---Q--DIEEAVVIT-R	QTQKS	--Y---TMK-RAA
	Angiostrongylus costaricensis	1529411915	-HE---Q--DIEEAVVIT-R	QTQKS	--Y---TMK-RAA
	Oesophagostomum dentatum	732742957	-HE---Q--DIEEAVVIT-R	QTQKS	--Y---TMK-RAA
	Haemonchus placei	1535211029	-HE---Q--DIEEAVVIT-R	QTQKS	--Y---TMK-RAA
	Haemonchus contortus	560135604	-HE---Q--DIEEAVVIT-R	QTQKS	--Y---TMK-RAA
	Heligmosomoides polygyrus	1530613144	-HE---Q--DIEEAVVIT-R	QTQKS	--Y---TMK-RAA
	Diploscapter pachys	1240347977	-HE---Q--DIEEAVVIT-R	QTQKS	--Y---TMK-RAA
	Ancylostoma ceylanicum	510862001	-HE---Q--DIEEAVVIT-R	QTQKS	--Y---TMK-RAA
	Dictyocaulus viviparus	768182347	-HE---Q--DIEEAVVIT-R	QTQKS	--Y---TMK-RAA
	Nippostrongylus brasiliensis	1529650831	-HE---Q--DIEEAVVIT-R	QTQKS	--Y---TMK-RAA
	Necator americanus	915279951	-HE---Q--DIEEAVVIT-R	QTQKS	--Y---TMK-RAA
	Toxocara canis	1535096396	-HE---Q--EIEEAVVIT-R	QTQKS	--Y---TMK-RAA
	Ancylostoma caninum	1432398790	-HE---Q--DIEEAVVIT-R	QTQKS	--Y---TMK-RAA
	Enterobius vermicularis	1529000477	-HE---Q--EIEEAVVIT-R	QTQKS	--Y---TMK-RA-
	Onchocerca ochengi	1528797026	-HE---Q--EIEEAVVIT-R	QTQKS	--Y---TMK-RAA
	Litomosoides sigmodontis	1528633243	-HE---Q--EIEEAVVIT-R	QTQKS	--Y---TMK-RAA
	Dracunculus medinensis	1535172495	-HE---Q--EIEEAVVIT-R	QTQKS	--Y---TMK--AA
	Loa loa	1158600458	-HE---Q--EIEEAVVIT-R	QTQKS	--Y---TMK-RAA
	Acanthocheilonema viteae	1511224209	-HE---Q--EIEEAVVIT-R	QTQKS	--Y---TMK-RAA
	Anisakis simplex	1528893083	-HE---Q--EIEEAVVIT-R	QTQKS	--Y---TMK-RAA
	Thelazia callipaeda	1528655218	-HE---Q--EIEEAVVIT-R	QTQKS	--Y---TMK-RVA
	Wuchereria bancrofti	1528678708	-HE---Q--EIEEAVVIT-R	QTQKS	--Y---TMK-RVA
	Brugia pahangi	1528772760	-HE---Q--EIEEAVVIT-R	QTQKS	--Y---TMK-RVA
	Brugia malayi	170577719	-HE---Q--EIEEAVVIT-R	QTQKS	--Y---TMK-RVA
	Brugia timori	1535296232	-HE---Q--EIEEAVVIT-R	QTQKS	--Y---TMK-RVA
	Pristionchus pacificus	1253290953	-HE---Q--EIEEAVVIT-R	QTQKS	--Y---TMK-RPA
	Soboliphyme baturini	1535368442	--Q---Q--DIEEAVVIT-R	QTGKS	--Y---TMKERS-
	Strongyloides ratti	1376136874	-HEF--VY-DIEEAVVIT-R	VTQKS	--Y---TMK-RAA
	Teladorsagia circumcincta	1276837198	--D--GR--EVNECMVMR-P	ATKRA	-----IT-V-PA-
	Onchocerca flexuosa	1528692356	-KAA--S--EISEAKVIR-P	HTLKS	K-Y---S-PF-E-
Other Nematodes (0/13)	*Trichinella pseudospiralis*	954298993	--E---Q--EIEEAVVIT-R	QTGKS	--Y---TMK-RP-
	Trichinella papuae	954590396	--E---Q--EIEEAVVIT-R	QTGKS	--Y---TMK-RP-
	Trichinella zimbabwensis	954482541	--E---Q--EIEEAVVIT-R	QTGKS	--Y---TMK-RP-
	Trichinella sp. T8	954617935	--EH--QY-EIEEAVVIT-R	QTGKS	--Y---TMK-RP-
	Trichinella patagoniensis	954337818	--EH--QY-EIEEAVVIT-R	QTGKS	--Y---TMK-RP-
	Trichinella nativa	954527271	--EH--QY-EIEEAVVIT-R	QTGKS	--Y---TMK-RP-
	Trichinella nelsoni	954210113	--EH--QY-EIEEAVVIT-R	QTGKS	--Y---TMK-RP-
	Trichinella sp. T9	954260219	--EH--QY-EIEEAVVIT-R	QTGKS	--Y---TMK-RP-
	Trichinella spiralis	954362426	--EH--QY-EIEEAVVIT-R	QTGKS	--Y---TMK-RP-
	Trichinella murrelli	954252124	--EH--QY-EIEEAVVIT-R	QTGKS	--Y---TMK-RP-
	Trichinella sp. T6	954293473	--EH--QY-EIEEAVVIT-R	QTGKS	--Y---TMK-RP-
	Trichinella britovi	954386464	--EH--QY-EIEEAVVIT-R	QTGKS	--Y---TMK-RP-
	Trichuris suis	669324980	--E---Q--DIEEAVVIT-R	QTGKS	--Y---TMKERT-
Outgroups	*Acropora digitifera*	XP_015752827	-TNH-KT---VTESKLMF-K	VTNRH	------T-QTEDD
	Cicer arietinum	XP_004514931	M-M---Q--EILEAVVIT-K	YTGRY	K-Y---T-K-PEA
	Falco peregrinus	XP_013159650	-KD--S---EVVDCTLKL-P	ITGRS	------L-KESE-

Outgroups (0/>100)	*Fukomys damarensis*	XP_010627066	-KD--S---EVVDCTLKL-P ITGRS ------L-KESE-
	Manihot esculenta	XP_021620866	--E--QA--EVVEAV-MK-R ATGRA --------A-PAV
	Homo sapiens	XP_016879636	-D--S---EIRECMVMR- P TTKRS ------T-A-PA-
	Mus musculus	BAC27370	-D--S---EIRECMVMR- P TTKRS ------T-T-PA-
	Saccharomyces cerevisiae	GAX68065	--E--G-Y-TVTDLK-MK-P ATGRS -----LS--KPS-
	Priapulus caudatus	XP_014673197	--E--S---EVTECMVMR-P TTKRS ------TYGNPEA

Supplementary Figure 49. Partial sequence alignments of a Heterogeneous nuclear ribonucleoprotein A1 protein with a CSI consisting of a 5 aa deletion (shown in black box) that is specific for the Chromadorea class. The CSI is not present in any non-nematode outgroup. There are two homologs of this protein in the *Caenorhabditis* genus, under the Chromadorea class, one of which contains the CSI.

Group	Species	Accession	221		252
Chromadorea (29/31)	*Caenorhabditis brenneri*	EGT30339	EEFKKMKDGKKSTT	Q	KAIEIYSEFVAEHSPKE
	Caenorhabditis briggsae	XP_002644390	--------------	-	-------------A---
	Caenorhabditis elegans	NP_508610	--------------	-	-----------------
	Caenorhabditis latens	OZG25613	--------------	-	------T------A---
	Caenorhabditis remanei	XP_003117797	--------------	-	------T------A---
	Ancylostoma ceylanicum	EPB80520	-----------A-I	-	--H--FK-Y--ASA---
	Ascaris suum	ERG79363	-------E---A--	-	R-YA--N-YI--Q----
	Brugia malayi	XP_001891554	---R---E-----I	-	--HS--NKYI--Q----
	Dictyocaulus viviparus	KJH43166	---R---E--RA-I	-	R-H--FK-Y--AAA---
	Haemonchus contortus	CDJ91770	-------E---A-I	-	R-H--FK-Y--AAA---
	Loa loa	XP_020301888	---R---E-----I	-	--HS--NKYI--Q----
	Necator americanus	XP_013290523	-------E---A-I	-	--H--FK-Y--AAA---
	Oesophagostomum dentatum	KHJ99479	-------E---A-I	-	--Q--FN----PA----
	Onchocerca flexuosa	OZC07703	---R---E-----I	-	--HS--NKYI--Q----
	Toxocara canis	KHN74888	-------E---A-I	-	R-HT--N-YI--Q----
	Wuchereria bancrofti	EJW88279	---R---E-----I	-	--HS--NKYI--Q----
	Diploscapter pachys	PAV84640	----NI--P-- LK	-	--H---K-----QA---
	Ancylostoma ceylanicum	EPB80520	-----------A-I	-	--H--FK-Y--ASA---
	Ascaris suum	ERG79363	-------E---A--	-	R-YA--N-YI--Q----
	Brugia malayi	XP_001891554	---R---E-----I	-	--HS--NKYI--Q----
	Dictyocaulus viviparus	KJH43166	---R---E--RA-I	-	R-H--FK-Y--AAA---
	Haemonchus contortus	CDJ91770	-------E---A-I	-	R-H--FK-Y--AAA---
	Loa loa	XP_020301888	---R---E-----I	-	--HS--NKYI--Q----
	Necator americanus	XP_013290523	-------E---A-I	-	--H--FK-Y--AAA---
	Oesophagostomum dentatum	KHJ99479	-------E---A-I	-	--Q--FN----PA----
	Onchocerca flexuosa	OZC07703	---R---E-----I	-	--HS--NKYI--Q----
	Toxocara canis	KHN74888	-------E---A-I	-	R-HT--N-YI--Q----
	Wuchereria bancrofti	EJW88279	---R---E-----I	-	--HS--NKYI--Q----
	Diploscapter pachys	PAV84640	----NI--P-- LK	-	--H---K-----QA---
	Pristionchus pacificus	PDM77096	--------------		--N---KK----G-LR-
	Strongyloides ratti	CEF63735	-------E----YN		--LH--DLYIH-DGS-Q
Outgroups (0/>100)	*Acanthisitta chloris*	XP_009078988	-----I-SQS-MVS		--KK-FA-YI-IQ-C--
	Amazona aestiva	KQK83323	-----I-SQS-MVS		--KK-FA-YI-IQ-C--
	Chelonia mydas	EMP31408	--Y--I-SQS-MVS		--KK-FA-YI-IQ-C--
	Danio rerio	XP_017211556	-DY--I-SQS-M-S		--KK-FA--I-IQ-C--
	Daphnia magna	KZS19170	-DY-HL- PQ-MA-		--QR--ND---VQAS--
	Homo sapiens	2OJ4_A	-D---V-SQS-MAS		--KK-FA-YI-IQAC--
	Mus musculus	NP_001334044	-----TRSTA-LV-		--HR-FE---DVQA-R-
	Xiphophorus maculatus	XP_005802346	--Y--I-SST-LVS		--NK-FK--IEVQ--R-

Supplementary Figure 50. Partial sequence alignments of a Regulator of G-protein signaling 7 protein with a CSI consisting of a 1 aa insertion (shown in black box) that is specific for the Chromadorea class. The CSI is not present in any non-nematode outgroup. The CSI is not present in the species *Pristionchus pacificus* and *Strongylodies ratti*.

Group	Species	Accession	210		245
Nematoda homolog 1 (42/42)	*Caenorhabditis elegans*	NP_001294068	GQRIVGVNGQLIYPTT	G	HKEVVALIKKDTMKTTLLV
	Caenorhabditis briggsae	CAP38400	--------------N-	-	--D--------N-------
	Caenorhabditis brenneri	EGT34238	---------E------	-	-----S-----N-------
	Caenorhabditis latens	OZG08793	---------E----N-	-	-----S-----N-------
	Caenorhabditis remanei	XP_003101224	---------E----N-	-	-----S-----N-------
	Caenorhabditis nigoni	PIC32830	--------------N-	-	-----------N-------
	Haemonchus contortus	CDJ86857	---------V-V--N-	P	------Q--RSPLR-----
	Diploscapter pachys	PAV68526	--------DT-V----	P	---------RSSLQ-----
	Pristionchus pacificus	PDM75425	A--------I--H---	P	----------NPVS-----
	Strongyloides ratti	CEF59908	------I-N-I---N-	P	-----T---RTPLEAR---
	Toxocara canis	KHN71588	---------S------	P	-----G---MNPLQ-D---
	Wuchereria bancrofti	EJW83604	--------------S-	A	-----S----NPLR-E---
	Brugia malayi	XP_001899140	--------------S-	A	-----S----NPLR-E---
	Loa loa	XP_020302439	--------------S-	A	-----S----NPLR-E---
	Ancylostoma duodenale	KIH63028	---------V-V--N-	P	------Q--RSPLR-----
	Ancylostoma ceylanicum	EYC04072	---------V-V--N-	P	------Q--RSPLR-----
	Brugia pahangi	VDN81659	--------------S-	A	-----S----NPLR-E---
	Brugia timori	VDO28175	--------------S-	A	-----S----NPLR-E---
	Onchocerca ochengi	VDK65450	--------------S-	A	-----S----NPLR-E---
	Litomosoides sigmodontis	VDK77581	--------------S-	A	-----S---TNPLR-E---
	Dracunculus medinensis	VDN52796	--------------Y-	P	--D--S---M-PLR-D---
	Heligmosomoides polygyrus	VDP23533	---------V-V--N-	P	------Q--RSPLR-----
	Anisakis simplex	VDK51478	---------H------	P	-----G---MNPLQ-D---
	Haemonchus placei	VDO07548	---------V-V--N-	P	------Q--RSPLR-----
	Nippostrongylus brasiliensis	VDL66851	---------V-V--N-	P	------H--RSPLR-----
	Angiostrongylus costaricensis	VDM53718	---------V-V--N-	P	------Q--RSPLR-----
	Teladorsagia circumcincta	PIO64747	---------M-V--N-	P	------Q--RSPLR-----
	Ancylostoma caninum	RCN49057	---------V-V--N-	P	------Q--RSPLR-----
	Necator americanus	XP_013301750	---------V-V--N-	P	------Q--RSPLR-----
	Dictyocaulus viviparus	KJH47934	---------V-V--N-	P	------Q--RSPLC-----
	Enterobius vermicularis	VDD95482	---------K----S-	P	YRD------ENPLV-E---
	Soboliphyme baturini	VDP38301	---------S-VFVD-	P	-----K---QQPASVE---
	Oesophagostomum dentatum	KHJ91366	---------V-V--N-	P	------Q--RSPLR-----
	Onchocerca flexuosa	OZC04861	--------------S-	A	-----S----NPLR-E---
	Trichinella patagoniensis	KRY22666	--------NT---VSS	S	--D--R---Q-PSGVQ---
	Trichinella papuae	KRZ78304	--------NT---VSS	S	--D--R---Q-PSGVQ---
	Trichinella pseudospiralis	KRY00923	--------NT---VSS	S	--D--R---Q-PSGVQ---
	Trichinella spiralis	XP_003374528	--------NT---VSS	S	--D--R---Q-PSGVQ---
	Trichinella murrelli	KRX50132	--------NT---VSS	S	--D--R---Q-PSGVQ---
	Trichinella nativa	KRZ61057	--------NT---VSS	S	--D--R---Q-PSGVQ---
	Trichuris trichiura	CDW52236	--------QTI--I-S	S	--D--R---Q-P-QVE---
	Trichuris suis	KFD54368	--------QT---I-S	S	--D--R---Q-P-QVE---
Nematoda homolog 2 (0/35)	*Dictyocaulus viviparus*	KJH47935	-D--FA---HS-IGES		--K--ER--ENAVRCEM--
	Ancylostoma ceylanicum	EPB74572	-D--FA---HS-IGES		--K--ER--ENAIRCEM--
	Necator americanus	XP_013301751	-D--YA---HS-IGES		--K--ER--ENAVRCEM--
	Haemonchus contortus	CDJ86857	-D--FA---HS-IGES		--K--ER--ENATRCEM--
	Caenorhabditis nigoni	PIC32832	-D--FA---HS-IGEN		--K--ER--ANPNRCEM--
	Caenorhabditis remanei	OZF76366	-D--FA---HS-IGEN		--K--ER--ANPNRCEM--
	Caenorhabditis latens	OZG08787	-D--FA---HS-IGEN		--K--ER--ANPNRCEM--
	Caenorhabditis elegans	NP_001294068	-D--FA---HS-IGEN		--K--ER--ANPNRCEM--
	Caenorhabditis briggsae	CAP38400	-D--FA---HS-IGEN		--K--ER--ANPNRCEM--
	Caenorhabditis brenneri	EGT34238	-D--YA---HS-IGEN		--K--ER--SNPNRCEM--
	Diploscapter pachys	PAV68526	-D--FA----S-VGEN		--K--ER--QNPNRCEM--
	Pristionchus pacificus	PDM75425	-D--FA---VS-QGE-		--K--ER--Q-P-RCE---
	Strongyloides ratti	CEF59908	-D--FA---A-VQGES		--Q-IQR--ENPLQCE---
	Toxocara canis	KHN71588	-D--FA---HS-IGEN		--Q--QR--ENPLQCE---
	Ancylostoma duodenale	KIH63027	-D--FA---HS-IGES		--K--ER--ENAIRCEM--
	Heligmosomoides polygyrus	VDO87852	-D--FA---HS-VGES		--K--ER--ENAVRCEM--
	Ancylostoma caninum	RCN49058	-D--FA---HS-IGES		--K--ER--ENAIRCEM--
	Haemonchus placei	VDO77662	-D--FA---HS-IGES		--K--ER--ENATRCEM--
	Angiostrongylus costaricensis	VDM53717	-D--FA---HN-IGES		--K--ER--ENALRCEM--
	Nippostrongylus brasiliensis	VDL68338	-D--FA---HS-SGES		--K--ER--ENSTRCEM--
	Anisakis simplex	VDK51478	-D--FA---HS-IGEN		--R--ER--ENPLQCE---
	Enterobius vermicularis	VDD95482	-D--YA----N-VGES		-RQ--QK--ENPLRCE---
	Thelazia callipaeda	VDM97278	-D--FA---HS-VGLN		-RQ--QR--ENPLQCE---
	Dracunculus medinensis	VDN52796	-DY-YA---VS-IGEN		--Q--QR--ENPLHCE---
	Litomosoides sigmodontis	VDK77581	-D-VLV---HC-IDEN		--K--QR--ENPLQCE-I-
	Oesophagostomum dentatum	KHJ82768	-DVV-A---FPVLAES		--AL-D-MSSQLNLCLV-L
	Ancylostoma ceylanicum	EYC04066	-D--FA---HS-IGES		--K--ER--ENAIRCEM--
	Trichinella patagoniensis	KRY22666	-D---A---VYVADQP		--D---K--E-PLQCR-T-
	Trichinella papuae	KRZ78304	-D---A---VYVADQP		--D---K--E-PLQCR-T-
	Trichinella pseudospiralis	KRY00923	-D---A---VYVADQP		--D---K--E-PLQCR-T-
	Trichinella spiralis	XP_003374528	-D---A---VYVADQP		--D---K--E-PLQCR-T-
	Trichinella murrelli	KRX50132	-D---A---VYVADQP		--D---K--E-PLQCR-T-
	Trichinella nativa	KRZ61057	-D---A---IYVADQP		--D---K--E-PLQCR-T-
	Trichuris trichiura	CDW52236	-D---A---TSVAELS		------R--E-PL-CR-T-
	Trichuris suis	KFD54368	-D---A---TPVAELS		------R--E-PL-CR-T-

Outgroups (0/>200)	*Acanthochromis polyacanthus*	XP_022058038	-D-L-F---EDVENES	-QQ--SR-RATEGRLE-I-
	Aedes albopictus	KXJ71430	-D--IE---TN-TTE-	--K--E---GVPNE-K---
	Aethina tumida	XP_019865902	-D--LE---ES-ANK-	--Q--E---AQSGE-K---
	Agrilus planipennis	XP_018333727	-D--LE---ES-ADKS	--Q--EA--ALPNE-K---
	Homo sapiens	AAH14513	-D-L-E---VNVEGE-	-HQ--QR--AVEGQ-R---
	Octopus bimaculoides	KOF98109	-D--IE---VN-GNEN	-QQ--QR--AGGEE-R---
	Mus musculus	AAD49224	-D-L-E---ENVEKE-	-QQ--SR-RAALNAVR---
	Rhinolophus sinicus	XP_019568739	-D-L-E---VNVEGE-	-HQ--QR--AVEGQ-Q---
	Salmo salar	XP_014058979	-D-L-F---ENVESES	-QQ--SR-RATVGQLE-I-

Supplementary Figure 51. Partial sequence alignments of an Na(+)/H(+) Exchange Regulatory Factor protein with a CSI consisting of a 1 aa insertion (shown in black box) that is specific for the entire Nematoda phylum. The CSI is not present in any non-nematode outgroup. There are two homologs of this protein in all Nematoda species, one of which contains the CSI.
